# Synthesis of the Proposed Structure of Celacarfurine and Analogues Using Sequential Cascade Ring Expansion Reactions

**DOI:** 10.1021/acs.orglett.5c05328

**Published:** 2026-01-21

**Authors:** Jerry K. F. Tam, Lachlan J. N. Waddell, Kleopas Y. Palate, Adrian C. Whitwood, Alexandra Longcake, Michael R. Probert, Gideon Grogan, Benjamin R. Lichman, William P. Unsworth

**Affiliations:** † 8748 University of York, Department of Chemistry, Heslington, York YO10 5DD, U.K.; ‡ University of York, Department of Biology, Heslington, York YO10 5DD, U.K.; § School of Natural and Environmental Sciences, 5994 Newcastle University, Newcastle Upon Tyne NE1 7RU, U.K.

## Abstract

The first synthesis of the proposed structure of spermidine derived macrocyclic alkaloid celacarfurine is described. A versatile synthetic strategy has been developed based on sequential cascade ring expansion reactions, with high dilution conditions not needed for any of the steps. The same general strategy was also used to generate a series of macrocyclic analogues. The physical properties and spectroscopic data obtained for our synthetic product do not match those reported for the isolated alkaloid.

## Graphical abstract

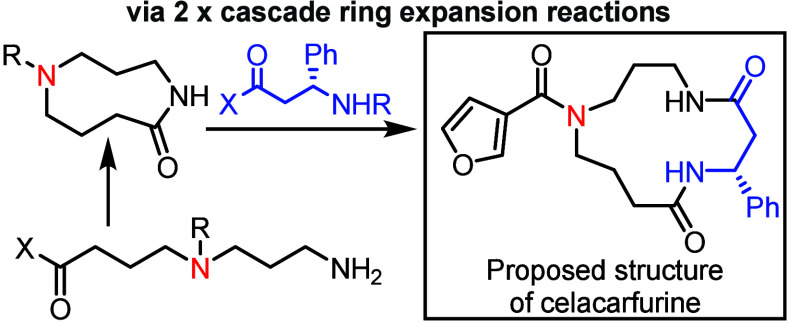

Polyamine alkaloids derived from spermidine are widely prevalent across the natural world, playing crucial roles in multiple organisms spanning animals, plants, bacteria and fungi.^1,2^ Within this class, 13-membered ring macrocyclic alkaloids feature prominently, with >50 natural products of this type reported.^1,3^ The majority possess a macrocyclic skeleton typified by celacinnine **1a**, with variations in the groups R^1^ and R^2^ in compounds of the type **1** accounting for much of the natural diversity (e.g., **1a**–**c**, Figure 1). Several successful total syntheses of alkaloids in this class have been reported,^4^ using both direct end-to-end macrocyclization,^5^ and ring expansion approaches.^6^

**Figure 1:**
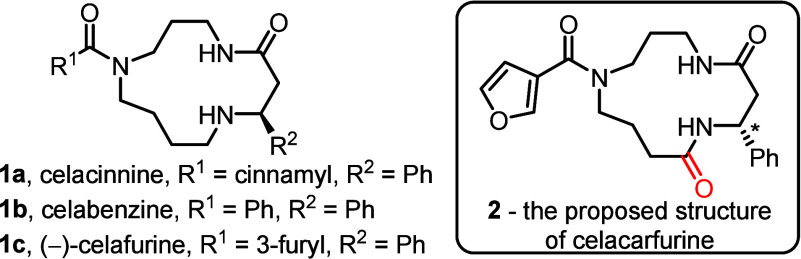
Celacinnine-type spermidine alkaloids.

In 2020, a new 13-membered macrocyclic spermidine alkaloid was reported by Liu and co-workers, named celacarfurine **2**, in view of its similarity to the previously reported alkaloid (−)-celafurine **1c**.^7^ Celacarfurine **2** was isolated from the roots of *Tripterygium wilfordii*, a plant in the Celastraceae family used in Chinese traditional medicine.

In this manuscript, we describe the first synthesis of the proposed structure of celacarfurine, and a series of analogues, using sequential cascade ring expansion reactions. At the onset of this project, the assigned structure of celacarfurine **2** had two unusual features that piqued our interest: (1) the absolute configuration of the sole stereogenic center (Figure 1, highlighted with *) is opposite to that in known celacinnine-type alkaloids;^8^ (2) there is a second carbonyl group in the macrocycle scaffold (Figure 1, highlighted in red), not present in any other reported celacinnine-type alkaloids. Total synthesis represents a useful way to validate the proposed structure of **2**, but to the best of our knowledge, no synthetic studies toward **2** had been reported prior to this study. A general synthetic approach to **2** was therefore devised, utilizing two distinct cascade ring expansion methods,^9^ both developed in our laboratory (CRE **1** and **2**, Scheme 1).^10–12^

**Scheme 1:**
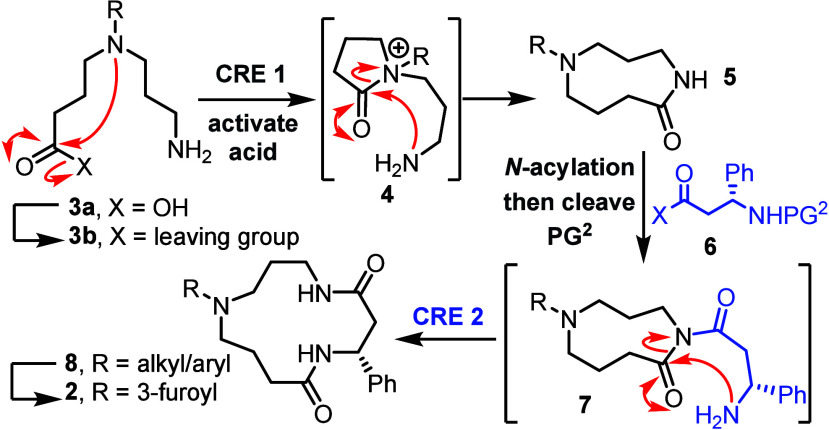
Synthetic Approach to **2**

We envisioned using a relatively simple amino acid derivative of the form **3a** as a key building block. The tertiary amine group in **3a**/**3b** is key in enabling the first cascade ring expansion; following carboxylic acid activation (**3a → 3b**) cyclization to form a 5-membered ring acyl ammonium intermediate (**3b → 4**) and spontaneous ring expansion (**4 → 5**, CRE **1**) was anticipated, to form a 9-membered ring lactam **5**.^10^ Then, *N*-acylation with a suitably protected β-amino acid derivative **6** and protecting group cleavage was planned, to form an imide **7** primed to undergo a second cascade ring expansion (CRE **2**)^11^ and generate the target 13-membered bis-lactam framework **8**. Based on our previous work,^10^ for the first cascade to work well an internal tertiary amine group is required (i.e., R = alkyl); therefore, cleavage of the exocyclic group R of **8** and replacement with a 3-furoyl group would then be needed complete the synthesis of **2**. The successful implantation of this synthetic approach is described herein – demonstrated in the synthesis a series of natural product-like 13-membered ring polyamine macrocycles, and in the first total synthesis of the proposed of structure of celacarfurine.^7^

To start, protected amino acids **11a** and **11b** were synthesized in high yields via sequential reductive amination and *N*-alkylation reactions, while **11c** was made via an Ullmann-type coupling followed by *N*-alkylation (Scheme 2). Substrates bearing different R groups on the tertiary amine (**11a**–**c**, R = Bn, PMB and PMP) were chosen that could potentially be cleaved later in the synthesis. In all three cases, acid-mediated protecting group cleavage revealed the key amino acid building block **12**, which was then used directly in the first cascade ring expansion using our published conditions, affording 9-membered lactams **13a**–**c** in good overall yields in each case. Employing a ring expansion approach within our synthetic strategy (as opposed to direct end-to-end cyclization) permits efficient lactam formation under typical reaction concentrations (0.1 M), thus avoiding the need for high-dilution conditions. This approach also facilitated their synthesis on a gram-scale.

**Scheme 2:**
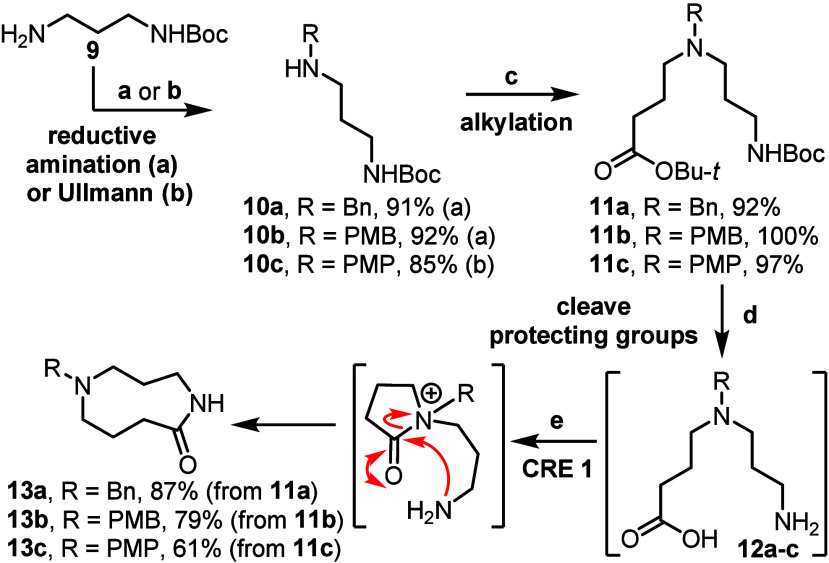
CRE **1** to Form 9-Membered Ring Lactams **13a**–**c** ^a^

a Reaction conditions: (a) amine **9**, aldehyde, MeOH, 70 °C then NaBH_4_, RT; (b) amine **9**, K_2_CO_3_, l-proline, CuI, 4-iodoanisole, DMSO, 70 °C; (c) amine **10**, *i*-Pr_2_NEt, *tert*-butyl-4-bromobutryate, 70 °C; (d) 4 M HCl in 1,4-dioxane, RT; (e) amino acid **12**, *i*-Pr_2_NEt, T3P, CH_2_Cl_2_, RT.

With 9-membered ring lactams **13a**–**c** in hand, the second ring expansion was then tested, using lactam **13a** and our conjugate addition/ring expansion cascade method (Scheme 3).^11a,11d,11e^ First, lactam **13a** was converted into imide **14** by reaction with acryloyl chloride under basic conditions. Then, reaction with seven different primary amines initiated the ring expansion cascade, via a conjugate addition (**14 → 15**) and ring expansion (**15 → 16**), affording 13-memebered ring lactams **16a**–**g**, all in good yields. In this model system, the use of imide **14** means that the macrocycles synthesized in this series all lack the requisite phenyl substituent needed to generate celacarfurine **2**.^13^ Nonetheless, these successful transformations validated our sequential ring expansion cascade concept and led to the facile synthesis of seven celacarfurine analogues. Successful rearrangement was confirmed by full characterization of all macrocycles **16a**–**g** (see the Supporting Information (SI)) and in the case of macrocycles **16b** and **16e**, further supported by X-ray crystallographic data.^14^

**Scheme 3:**
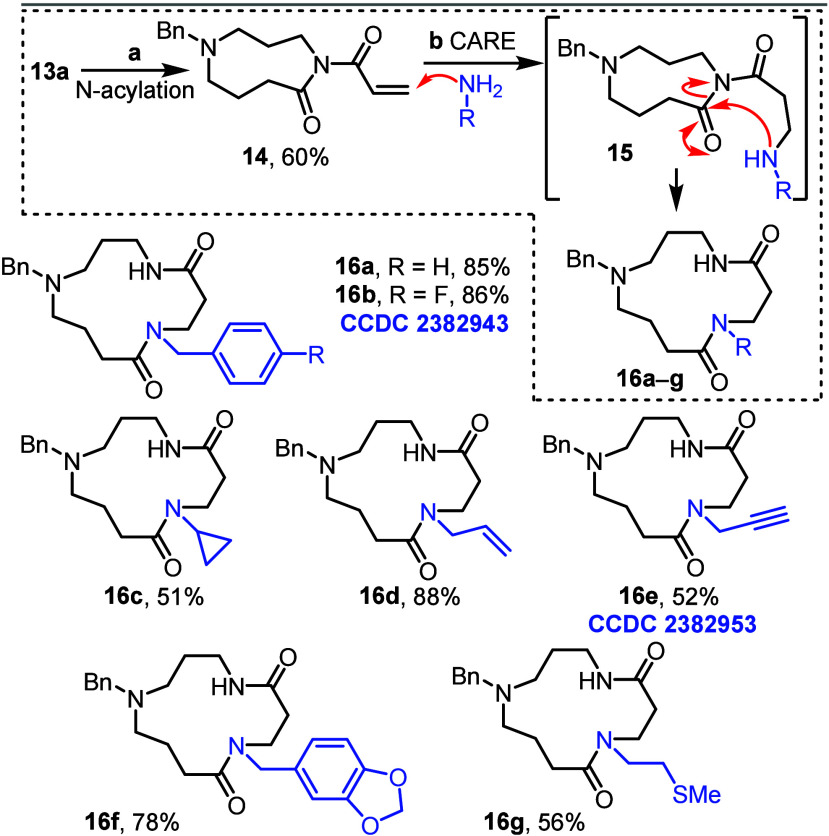
Conjugate Addition/Ring Expansion Cascade Reactions of **13a**: Analogue Synthesis^a^

a Reaction conditions: (a) lactam **13**, LiHMDS, acryloyl chloride, THF, 0 °C to RT; (b) imide **14**, amine, THF, RT.

To form the phenyl substituted 13-membered ring celacarfurine scaffold, we then turned to an alternative lactam ring expansion.^11b^ The ring expansion reactions summarized in Scheme 4 started with lactam *N*-acylation with a carbamate-protected amino acid chloride of the form **17**. Following *N*-acylation, imides (**18a**–**e**) are formed, and cleavage of the carbamate protecting groups reveals the reactive amine group, enabling spontaneous ring expansion (**18 → 19**). In this way, 13-membered ring lactam **19a** was generated from lactam **13a** and Cbz-protected amino acid chloride **17a** (R^2^ = Ph, R^3^ = H, PG = Cbz), with the protecting group cleavage and spontaneous ring expansion promoted via hydrogenolysis. Lactam **19a** was isolated in 36% yield over the *N-*acylation, protecting group cleavage and ring expansion sequence, with structure confirmed by X-ray crystallographic data of its HCl salt.^14^ Alternatively, lactam **19b**, with the same 13-membered ring framework, was formed in a much higher overall yield (84%) starting from lactam **13c**, using an Fmoc-protected amino acid chloride, with the protecting group cleavage and ring expansion cascade promoted by DBU. To highlight the value of the ring expansion approach to generate analogues, macrocyclic lactams **19c**–**e** were also synthesized in good yields, each using Fmoc-protected amino acid chlorides. In addition, a 12-membered ring analogue **19f** was also synthesized via a 3-atom ring expansion of **13c** using a phenyl alanine derived acid chloride **17f**. A lower yield was obtained in this case, as expected based on our previous work using α-amino acids in this type of ring expansion,^11b^ but the reaction worked sufficiently well to afford **17f** and allow us to test a hypothesis discussed later in the manuscript.

**Scheme 4:**
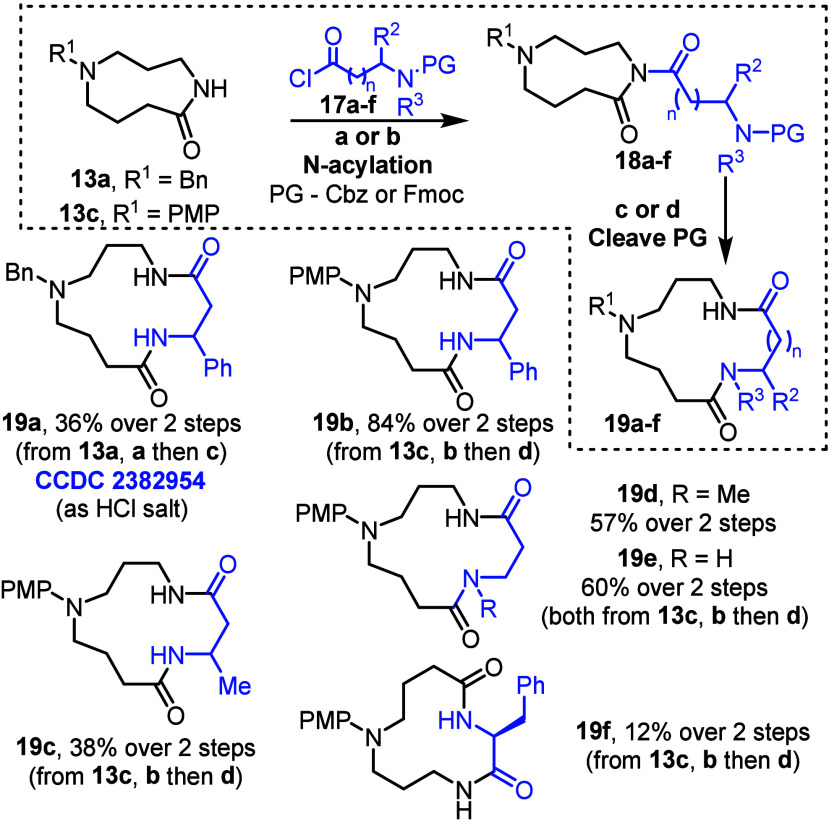
CRE **2** to Form Macrocycles **19a**–**f** ^a^

a Reaction conditions: (a) lactam **13a**, LiHMDS, acid chloride **17a**, THF, 0 °C to RT; (b) lactam **13b**, acid chloride **17b**–**f**, DMAP, pyridine, CH_2_Cl_2_, 50 °C; (c) imide **18a**, H_2_, palladium hydroxide (20 wt % on carbon), ethanol, RT; (d) DBU, CH_2_Cl_2_, RT.

To complete the synthesis, hydrogenolysis of benzylated macrocycle **19a** under acidic conditions enabled *N*-benzyl cleavage, to form secondary amine **21** in 88% yield (Scheme 5). The same product **21** was also obtained in quantitative yield from the analogous PMP-derivative **19b**, following oxidative cleavage using periodic acid. The structure of amine **21** was confirmed by analysis of the X-ray crystallographic data of its sulfuric acid salt,^14^ which importantly showed that the 13-membered ring scaffold remained intact following protecting group cleavage, with no evidence of unwanted ring-opening or ring-contraction.^10b^ The synthesis of **2** was then completed via a straightforward *N*-acylation of **21** using 3-furoic acid, activated by T3P.

**Scheme 5:**
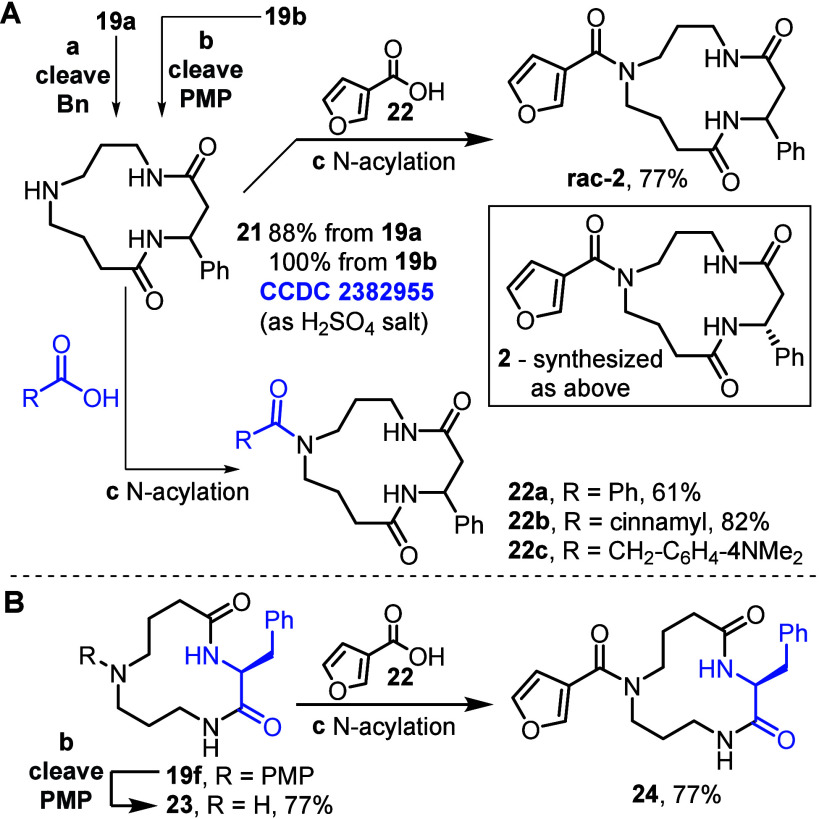
(A) Synthesis of **rac-2** and **2**; (B) Synthesis of 12-Membered Ring Isomer **24** ^a^

a Reaction conditions: (a) H_2_, palladium hydroxide (20 wt % on carbon), methanol, acetic acid, RT; (b) periodic acid, water CH_3_CN, RT; (c) 3-furoic acid **22**, *i*-Pr_2_NEt, T3P, CH_2_Cl_2_, RT.

The sequence summarized in Scheme 5A resulted in the formation of racemic macrocycle **rac-2**, but isolated celacarfurine was reported to be obtained as its *R*-enantiomer **2** (Scheme 5A box). The same route was therefore used to synthesize **2** in enantiopure form, using an enantiopure β-phenylalanine derivative. The spectroscopic data for the *R*-derivatives [**(** *R* **)-19b**, **(** *R* **)-21** and **2**] were identical to those the racemic analogues, with full synthetic details in the SI. As a simple demonstration of how this method could also be used to generate analogues, amine **21** was also converted into macrocyclic amides **22a–c**, of which **22a** and **22b** have an *N*-acyl substituents commonly found in celacinnine-type spermidine alkaloids.

Differences between our synthetic samples and the isolated natural product^7^ quickly became apparent. The isolated celacarfurine was originally reported to be characterized by ^1^H and ^13^C NMR in *d*_4_-methanol.^7^ However, in d_*4*_-methanol our synthetic samples (both **rac-2** and **2**) were only sparingly soluble, with the solubility too low to obtain ^13^C NMR data of sufficient quality to enable comparison with the isolation data. The solubility of **rac-2** and **2** was also too low to allow us to observe all signals in the ^1^H NMR spectrum in *d*_4_-methanol, although enough material dissolved to allow comparison of the phenyl and furan regions of the spectra, and significant differences were seen in all signals (see SI section 4 for full details).

We think that the ^1^H and ^13^C NMR were reported in *d*_4_-methanol in error.^7^ The same NMR data were subsequently described in a patent by the same team,^15^ with all signals being identical to those in the isolation paper.^7^ But crucially, the patent includes images of the ^1^H and ^13^C NMR spectra in which residual solvent signals consistent with the data being collected in *d*_6_-DMSO, not *d*_4_-methanol, are clearly visible. We therefore characterized **rac-2** and **2** in d*_6_*-DMSO instead. The synthetic samples dissolved well in *d*_6_-DMSO, and their NMR data support the assigned 13-membered macrocyclic structure. But unfortunately, major differences were evident when comparing the synthetic and isolated materials (see SI sections 3 and 4). Comparing the optical rotation of our synthetic compound **2** ([α]_D_ = +42.42) to the reported optical rotation value for the isolated material ([α]_D_ = +5.78)^7^ also showed a significant difference. The isolation team published a subsequent study on the effects of spermidine macrocyclic alkaloids, including celacarfurine, on the expression of amyloid β-peptide in SH-SY5Y cells.^16^ In this study, ^1^H and ^13^C NMR data for celacarfurine were reported in CDCl_3_. However, our synthetic materials were insoluble in CDCl_3_, representing another point of difference. We can therefore conclude beyond reasonable doubt that the celacarfurine isolated by Liu and co-workers^7^ and our synthetic material **2** are not the same.

One explanation for this difference is that we may have made a mistake during our synthesis. However, having prepared and fully characterized multiple 13-membered lactams in this study, including 4 compounds with supporting X-ray crystallographic data, we are confident in the assignment of our synthetic material. This notably includes X-ray data for macrocycle **21**, the direct precursor to **2**. Regrettably, we were unable to obtain X-ray data for **2**, as this would have provided even greater confidence; this is despite extensive efforts to crystallize our sample of **2**, including using the Encapsulated nanodroplet crystallization (ENaCt) method (see SI section 5).^17^

Therefore, we must also consider the possibility that the isolated material may have been misassigned.^18^ With this in mind, we considered that isomeric 12-membered macrocycle **24** (Scheme 5B) may also account for the reported data;^7^ the inclusion of a phenyl alanine unit in this alternative structure provides some biosynthetic justification to this alternative proposal. We therefore synthesized macrocycle **24** from **19f**, via sequential PMP-cleavage and *N*-acylation. Unfortunately, clear differences in the ^1^H and ^13^C NMR data for **24** were seen compared with isolated celacarfurine, thus ruling out this possibility.

A third possibility is that both the synthetic and isolated materials are correctly assigned, but they exist in different rotameric forms, thus accounting for their different physical properties and spectroscopic data. Without access to a sample of the isolated material, we cannot categorically rule this possibility out.^18^ Heating our synthetic sample of **2** in *d*_6_-DMSO for 1 h at 190 °C, cooling to RT, and reacquiring its ^1^H NMR spectrum resulted in no change in the appearance of its NMR data; this suggests that our synthetic material **2** was not formed as a higher energy rotamer compared to the natural material.

In conclusion, a strategy of using consecutive cascade ring expansion reactions has been used to generate natural product-like 13-membered ring polyamine macrocycles. High dilution conditions are not needed for any of the cascade ring expansion steps reported, which were able to deliver a series of structural analogues from common precursors. This included the first synthesis of the proposed of structure of celacarfurine, in 32% overall yield from protected diamine **9** (Scheme 6).

**Scheme 6:**
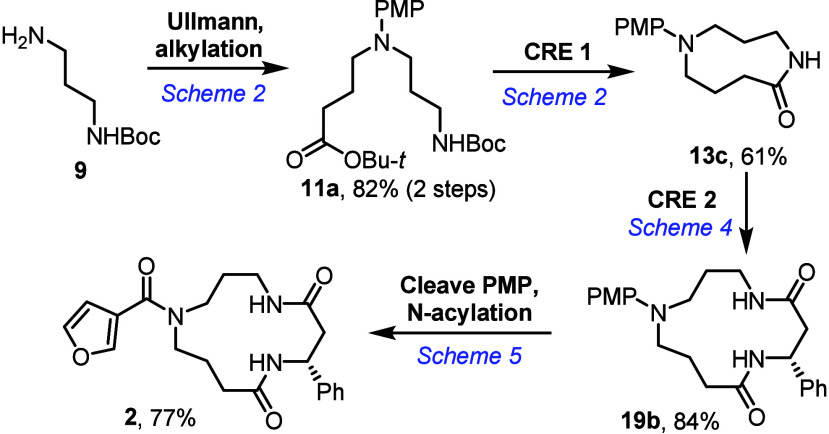
Summary of the Complete Synthetic Route to **2**

Unfortunately, the data obtained for our synthetic product do not match those reported for the isolated alkaloid. The results described herein highlight the value of sequential cascade ring expansion reactions for the efficient synthesis of complex macrocyclic target molecules. Similar approaches are expected to be applicable to other synthetic targets, e.g. other spermidine-derived macrocyclic alkaloids and analogues.^1,3^ It is therefore our hope that this study will inspire the development of related approaches to synthesize bioactive macrocycles,^9^ including other natural products and synthetic macrocycles for applications in medicinal chemistry.^19^

## Supplementary Material



## Data Availability

The data underlying this study are available in the published article and its Supporting Information.

## Notes

Experimental procedures, characterization data, copies of ^1^H and ^13^C NMR spectra, and X-ray data (PDF)

## ∥

Cancer Research Horizons, Babraham Research Campus, Cambridge CB22 3AT, U.K

## ⊥

Materials Innovation Factory, University of Liverpool, 51 Oxford Street, Liverpool L7 3NY, U.K.

## References

[1] Shi Y.-J., Zhang J., Wang Y.-W., Ding K., Yan Y., Xia C.-Y., Li X.-X., He J., Zhang W. K., Xu J.-K. (2022). The untapped
potential of spermidine alkaloids: structures, bioactivities
and syntheses. Eur. J. Med. Chem..

[2] da Silva G., Soengas R. G. (2017). Natural occurrence,
synthesis and
biological applications of spermidine alkaloids. Curr. Org. Chem..

[3] Bienz S., Detterbeck R., Ensch C., Guggisberg A., Häusermann U., Meisterhans C., Wendt B., Werner C., Hesse M. (2002). Putrescine, spermidine, spermine, and related polyamine alkaloids. Alkaloids Chem. Biol..

[4] Kuehne P., Linden A., Hesse M. (1996). Asymmetric Synthesis of the Alkaloids
mayfoline and N(1)-acetyl-N(1)-deoxymayfoline. Helv. Chim. Acta.

[5] Illuminati G., Mandolini L. (1981). Ring closure
reactions of bifunctional
molecules. Acc. Chem. Res..

[6] a Hesse, M. In Ring Enlargement in Organic Chemistry; Wiley-VCH: 1991.

[7] Liu J., Wu Q., Shu J., Zhang R., Liu L. (2020). A novel spermidine
macrocycle alkaloid from the roots of *Tripterygium wilfordii*. Chem. Nat. Compd..

[8] This assignment was made on the basis of celacarfurine **2** having positive optical rotation, opposite to (−)-celafurine **1c**.

[9] Wootton J. M., Tam J. K. F., Unsworth W. P. (2024). Cascade
ring expansion
reactions for the synthesis of medium-sized rings and macrocycles. Chem. Commun..

[10] Lawer A., Rossi-Ashton J. A., Stephens T. C., Challis B. J., Epton R. G., Lynam J. M., Unsworth W. P. (2019). Internal Nucleophilic
Catalyst Mediated Cyclisation/Ring
Expansion Cascades for the Synthesis of Medium-Sized Lactones and
Lactams. Angew. Chem., Int. Ed..

[11] Palate K. Y., Yang Z., Whitwood A. C., Unsworth W. P. (2022). Synthesis of medium-ring lactams and macrocyclic peptide
mimetics via conjugate addition/ring expansion cascade reactions. RSC Chem. Biol..

[12] Kitsiou C., Hindes J. J., I’Anson P., Jackson P., Wilson T. C., Daly E. K., Felstead H. R., Hearnshaw P., Unsworth W. P. (2015). The Synthesis of Structurally Diverse
Macrocycles by Successive Ring Expansion. Angew.
Chem., Int. Ed..

[13] In principle, the phenyl group could be incorporated into an alternative imide precursor derived from **13a** and cinnamoyl chloride. However, this was not attempted, as previous work has shown that cinnamoyl imides do not undergo the necessary aza-Michael reaction.^11a^

[14] CCDC 2382943 (**16b**), 2382953 (**16e**), 2381954 (**19a**·HCl) and 2382955 (**21**·H_2_SO_4_) contain the crystallographic data, see: www.ccdc.cam.ac.uk/data_request/cif.

[15] Liu, J. ; Wu, Q. ; Shu, J. ; Zhang, R. Preparation method of anti-inflammatory macrocyclic polyamine alkaloid celacarfurine. CN111484485B, April 5, 2022.

[16] Zhang L., Chen J., Liu L., Liu J., Liu L. (2024). Effects of
Spermidine Macrocyclic Alkaloids on the Expression of Amyloid β-Peptide
in SH-SY5Y Cells via Autophagy. Rev. Bras. Farmacogn..

[17] Tyler A. R., Ragbirsingh R., McMonagle C. J., Waddell P. G., Heaps S. E., Steed J. W., Thaw P., Hall M. J., Probert M. R. (2020). Encapsulated
Nanodroplet Crystallization of Organic-Soluble Small Molecules. Chem..

[18] We contacted the corresponding authors of the isolation paper^7^ to find out if any of the isolated material remains to allow further analysis but did not receive a response.

[19] Driggers E. M., Hale S. P., Lee J., Terrett N. K. (2008). The exploration
of macrocycles for drug discovery -
an underexploited structural class. Nat. Rev.
Drug Discovery.

