# Supplementary material for: Synthesis of the Proposed Structure of Celacarfurine and Analogues Using Sequential Cascade Ring Expansion Reactions
Source: Org Lett. 2026 Jan 21;28(4):1441–6. doi: 10.1021/acs.orglett.5c05328 (PMC12865764; doi:10.1021/acs.orglett.5c05328)
Supplement: Supplementary file 1 [file ol5c05328_si_001.pdf]

Supporting Information

Synthesis of the proposed structure of celacarfurine and analogues using sequential cascade ring expansion reactions

Jerry K. F. Tam,[a] Lachlan J. N. Waddell,[a] Kleopas Y. Palate,[a†] Adrian C. Whitwood,[a] Alexandra Longcake,[c†] Michael R. Probert,[c] Gideon Grogan,[a] Benjamin R. Lichman,[b] William P. Unsworth*[a]

[a] University of York, Department of Chemistry, Heslington, York, YO10 5DD (UK)

[b] University of York, Department of Biology, Heslington, York, YO10 5DD (UK)

[c] School of Natural and Environmental Sciences, Newcastle University, Newcastle Upon Tyne, NE1 7RU (UK)

Table of Contents

1) General Information	S3
2) General procedures	S4
3) Synthetic methods and compound data	S5–S34
4) Comparison of isolated and synthetic material data	S35–S38
5) Encapsulated nanodroplet crystallisation (ENaCt) attempts on macrocycle **2**	S39–S41
6) ^{1}H and ^{13}C NMR spectra	S42–S80
7) References	S81

1) General Information

All reactions were performed in oven-dried glassware under a nitrogen atmosphere. Unless specified, all reagents and starting materials were purchased from commercial companies and used as received. Anhydrous solvents were freshly obtained from an Innovative Technology Inc. PureSolv® solvent purification system following standard procedures. For all reactions requiring heating, the reaction mixture was heated using metal heating blocks. Analytical thin layer chromatography (TLC) was performed using pre-coated silica gel plates. Visualisation was achieved by UV light (254 nm) or $KMnO_4$ and ninhydrin as stain. Flash chromatography was performed using silica gel and gradient solvent system (eluent: hexane: ethyl aetate/hexane: DCM). ^{1}H and ^{13}C NMR spectra were recorded on 400 MHz Jeol ECS and Bruker AV and AM, or 700 MHz Bruker Avance Neo700 spectrometers. Chemical shifts (ppm) were recorded with tetramethylsilane (TMS) as the internal reference standard. Multiplicities are given as: s (singlet), br s (broad singlet), d (doublet), t (triplet), dd (doublet of doublets), dt (double of triples), ddd (doublet of doublet of doublets), ddt (doublet of doublet of triplets), dtt (doublet of triplet of triplets), ddq (doublet of doublet of quintets), dddd (doublet of doublet of doublet of doublets), dtd (doublet of triplet of doublets), dt (doublet of triplets), dp (doublet of pentents), dq (doublet of quintets), td (triplet of doublets), tdd (triplet of doublet of doublets), tt (triplet of triplets), qd (quintet of doublets), qt (quintet of triplets) or m (multiplet). The number of protons (*n*) for a given resonance is indicated by *n*H and coupling constants are reported as a *J* value in Hz. High resolution mass spectra (HRMS) were obtained on a LC/HRMS TOF spectrometer using simultaneous electrospray (ESI).

2) General procedures

General Procedure A: Conjugate Addition Ring Expansion (CARE)[1]

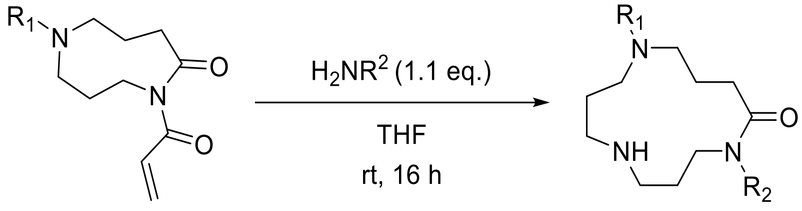

To a stirring solution of acryloyl imide (1 eq.) in THF (0.1 M) was added primary amine (1.1 eq.) at room temperature. The mixture was stirred for 16 hours at room temperature, after which the reaction was diluted with water and dichloromethane. The aqueous layer was extracted with dichloromethane (3×), the combined organic layers were dried with sodium sulfate, filtered and concentrated under vacuum. The crude product was purified via flash column chromatography.

General Procedure B: Fmoc Protection of amino acids[2]

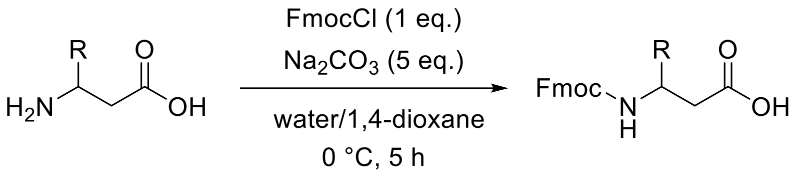

To a stirring solution of amino acid (1 eq.) and sodium carbonate (5 eq.) in water/1,4-dioxane (8:5 v/v, 0.08 M) was added a solution of FmocCl (1 eq.) in dioxane (0.3 M) dropwise at 0 °C. The mixture was stirred for 5 hours, followed by acidification to pH 1 using hydrochloric acid (2 M aqueous solution). The reaction mixture was extracted with ethyl acetate (3 ×), the combined organic layers were dried with sodium sulfate, filtered and concentrated under vacuum. The crude product was purified via flash column chromatography.

General procedure C: Acid Chloride Preparation[2]

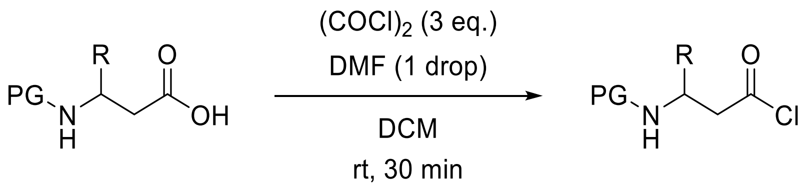

To a stirring suspension of carboxylic acid (1 eq.) in dichloromethane (0.3 M) was added oxalyl chloride (3 eq.) and 1 drop of dimethyl formamide. The reaction was stirred at room temperature for 30 minutes or until the solid suspension is completely dissolved. The solvent was removed under vacuum to yield the desired acid chloride.

3) Synthetic methods and compound data

***tert*-Butyl (3-(benzylamino)propyl)carbamate (10a)**

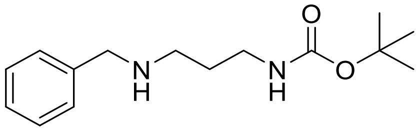

To a stirring solution of *N*-Boc-1,3-diaminopropane (514 mg, 2.9 mmol) in methanol (30 mL), benzaldehyde (0.30 mL, 2.9 mmol) was added and stirred at 70 °C for 30 minutes. The reaction was removed from the heat and sodium borohydride (134 mg, 3.6 mmol) was added to the solution in small portions. The reaction was then stirred at room temperature for 30 minutes, after which was quenched with sat. aqueous ammonium chloride (30 mL), extracted with ethyl acetate (3 × 30 mL), the combined organic phase dried with sodium sulfate, filtered, and concentrated under vacuum to afford the title compound as a colourless oil (705 mg, 2.7 mmol, 91%). R_f 0.41 (19:1 ethyl acetate:triethyl amine). ν_{max} (thin film)/cm^{-1} 3372, 2934, 2763, 2579, 1688, 1526, 1419, 1366, 1282, 1249, 1044, 869, 741, 697, 621. ^{1}H NMR (400 MHz, $CDCl_3$) δ 7.34–7.19 (5H, m, ArH), 5.37 (1H, br s, NH), 3.75 (2H, s, $PhCH_2$), 3.20 (2H, dd, *J* = 6.5, 6.5 Hz, CH_2CH_2NH), 2.69 (2H, t, *J* = 6.5 Hz, CH_2NH), 1.65 (2H, tt, *J* = 6.5, 6.5 Hz, $CH_2CH_2CH_2$), 1.43 (9H, s, CH_3), 1.43 (1H, br s, NH). ^{13}C NMR (101 MHz, $CDCl_3$) δ 156.2 (CO), 140.4 (ArC), 128.5 (ArCH), 128.2 (ArCH), 127.1 (ArCH), 79.0 ($C(CH_3)_3$), 54.1 ($PhCH_2$), 47.5 ($NHCH_2$), 39.6 ($NHCH_2$), 29.8 (CH_2), 28.5 ($C(CH_3)_3$). HRMS (ESI$^+$) $C_{15}H_{25}N_2O_2$ (MH^+) theoretical 265.1911; measured 265.1915 (−1.8 ppm error).

***tert*-Butyl (3-((4-methoxybenzyl)amino)propyl)carbamate (10b)**

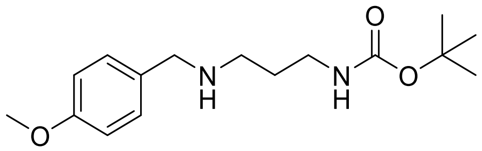

To a stirring solution of *N*-Boc-1,3-diaminopropane (2.00 g, 11.5 mmol) in methanol (115 mL), 4-methoxybenzaldehyde (1.40 mL, 11.5 mmol) was added and stirred at room temperature for 16 hours. Sodium borohydride (521 mg, 13.8 mmol) was added to the solution in small portions. The reaction was then stirred at room temperature for 1 hour. The reaction mixture was concentrated under vacuum, diluted with ethyl acetate (50 mL) and water (50 mL), the aqueous layer was extracted with ethyl acetate (3 × 50 mL). The combined organic phase dried with sodium sulfate, filtered, and concentrated under vacuum. The crude was purified via flash column chromatography (7:3 n-hexane:ethyl acetate→19:1 ethyl acetate:triethylamine) to afford the title compound as a white crystalline solid (3.10 g, 10.5 mmol, 92%). Mp 155 °C. R_f 0.32 (9:1 ethyl acetate:methanol). ν_{max} (thin film)/cm^{-1} 3333, 2975, 2933, 2835, 1692, 1612, 1511, 1454, 1365, 1244, 1168, 1034, 817, 572, 518. ^{1}H

NMR (400 MHz, $CDCl_3$) δ 7.24 (2H, d, *J* = 8.5 Hz, ArH), 6.85 (2H, d, *J* = 8.5 Hz, ArH), 3.80 (3H, s, OCH_3), 3.71 (2H, s, $ArCH_2$), 3.25–3.17 (2H, m, $NHCH_2$), 2.69 (2H, t, *J* = 6.5 Hz, $NHCH_2$), 1.66 (2H, tt, *J* = 6.5, 6.5 Hz, CH_2), 1.44 (9H, s, $C(CH_3)_3$). ^{13}C NMR (101 MHz, $CDCl_3$) δ 158.8 (ArC), 156.2 (CO), 132.5 (ArC), 129.5 (ArC), 113.9 (ArC), 79.1 ($C(CH_3)_3$), 55.4 (OCH_3), 53.5 ($ArCH_2$), 47.5 ($NHCH_2$), 39.7 ($CONHCH_2$), 29.8 ($CH_2CH_2CH_2$), 28.6 ($C(CH_3)_3$). HRMS (ESI^+) $C_{16}H_{27}N_2O_3$ (MH^+) theoretical 295.2016; measured 295.2019 (−0.8 ppm error).

***tert*-Butyl (3-((4-methoxyphenyl)amino)propyl)carbamate (10c)**

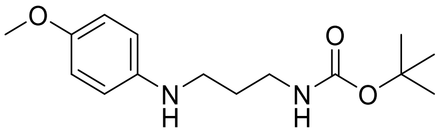

To a stirring solution of *N*-Boc-1,3-diaminopropane (13.5 g, 78 mmol), potassium carbonate (17.7 g, 128 mmol), L-proline (1.48 g, 12.8 mmol) and copper(I) iodide (1.22 g, 6.4 mmol) in DMSO (40 mL), 4-iodoanisole (15.1 g, 64.5 mmol) was added and stirred at 70 °C for 16 hours. The reaction was removed from the heat and diluted with water (100 mL) and dichloromethane (100 mL), the aqueous layer was extracted with dichloromethane (3 × 100 mL), the combined organic phase dried with sodium sulfate, filtered, and concentrated under vacuum and purified via flash column chromatography (1:1 n-hexane:diethyl ether) to afford the title compound as a yellow crystalline solid (15.3 g, 54.9 mmol, 85%). Mp 56 °C. R_f 0.23 (1:1 n-hexane:diethyl ether). ν_{max} (thin film)/cm^{-1} 3362, 2934, 1696, 1513, 1366, 1237, 1170, 1038, 821. ^1H NMR (400 MHz, $CDCl_3$) δ 6.76 (2H, d, *J* = 9.0 Hz, ArH), 6.57 (2H, d, *J* = 9.0 Hz, ArH), 4.86 (1H, br s, NH), 3.72 (2H, s, OCH_3), 3.25–3.16 (2H, m, $NHCH_2$), 3.10 (2H, t, *J* = 6.5 Hz, $NHCH_2$), 1.73 (2H, tt, *J* = 6.5, 6.5 Hz, $NHCH_2CH_2$), 1.44 (9H, s, $C(CH_3)_3$). ^{13}C NMR (101 MHz, $CDCl_3$) δ 156.3 (CO), 152.1 (ArC), 142.5 (ArC), 114.9 (ArCH), 114.3 (ArCH), 79.2 ($C(CH_3)_3$), 55.8 (OCH_3), 42.1 ($NHCH_2$), 38.2 ($NHCH_2$), 29.7 (CH_2), 28.4 ($C(CH_3)_3$). HRMS (ESI^+) $C_{15}H_{25}N_2O_3$ (MH^+) theoretical 281.1860; measured 281.1869 (−3.3 ppm error).

tert-Butyl 4-(benzyl(3-((tert-butoxycarbonyl)amino)propyl)amino)butanoate (**11a**)

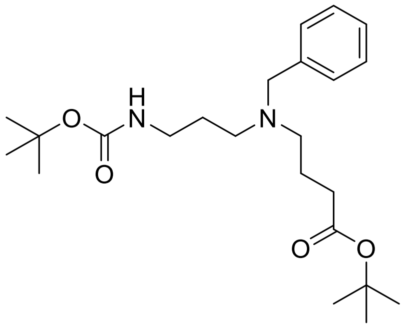

To a stirring solution of amine **10a** (6.45 g, 24.4 mmol) in diisopropyl ethylamine (8.50 mL), *tert*-butyl-4-bromobutryate (6.50 mL, 36.7 mmol) was added and stirred at 70 °C for 72 hours. The reaction was diluted with water (100 mL), extracted with ethyl acetate (3 × 100 mL), the combined organic phase was concentrated under vacuum. The residue was acidified with 20% aqueous acetic acid (140 mL), extracted with diethyl ether (250 mL). The aqueous layer was basified with 5M aqueous sodium hydroxide until pH 14. The solution was extracted with dichloromethane (3 × 150 mL), dried with sodium sulfate, filtered, and concentrated under vacuum to afford the title compound as a yellow oil (9.09 g, 22.4 mmol, 92%). R_f 0.29 (7:3 n-hexane:ethyl acetate). ν_{max} (thin film)/cm^{-1} 3374, 2977, 2933, 2808, 2250, 1703, 1510, 1454, 1366, 1249, 1153, 911, 730, 699. ^{1}H NMR (400 MHz, $CDCl_3$) δ 7.36–7.13 (5H, m, ArH), 5.35 (1H, br s, NH), 3.51 (2H, s, $PhCH_2$), 3.18–3.08 (2H, m, CH_2NH), 2.46 (2H, t, *J* = 6.0 Hz, CH_2N), 2.40 (2H, t, *J* = 7.0 Hz, CH_2N), 2.21 (2H, t, *J* = 7.5 Hz, $COCH_2$), 1.82–1.69 (2H, m, CH_2), 1.69–1.52 (2H, m, CH_2), 1.46–1.38 (18H, m, $C(CH_3)_3$). ^{13}C NMR (101 MHz, $CDCl_3$) δ 173.1 (CO), 156.1 (CO), 139.5 (ArC), 129.0 (ArCH), 128.4 (ArCH), 127.1 (ArCH), 80.2 ($C(CH_3)$), 78.8 ($C(CH_3)$), 58.8 ($PhCH_2$), 53.0 (NCH_2), 52.2 (NCH_2), 39.7 ($NHCH_2$), 33.3 ($COCH_2$), 28.6 (CH_3), 28.2 (CH_3), 26.6 (CH_2), 22.5 (CH_2). HRMS (ESI$^+$) $C_{23}H_{39}N_2O_4$ (MH^+) theoretical 407.2904; measured 407.2910 (−1.5 ppm error).

tert-Butyl 4-((3-((tert-butoxycarbonyl)amino)propyl)(4-methoxybenzyl)amino)butanoate (**11b**)

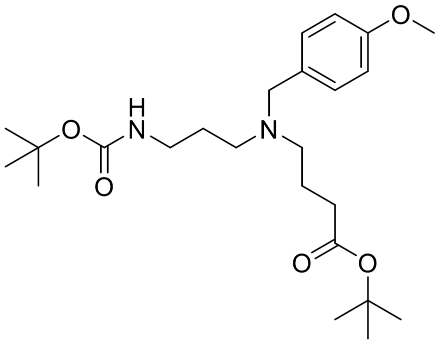

To a stirring solution of amine **10b** (680 mg, 2.3 mmol) in diisopropyl ethylamine (0.80 mL), *tert*-butyl-4-bromobutryate (0.61 mL, 3.4 mmol) was added and stirred at 70 °C for 72 hours. The reaction was diluted with water (20 mL), extracted with ethyl acetate (3 × 20 mL), the combined organic phase was

concentrated under vacuum. The residue was acidified with 20% aqueous acetic acid (30 mL), extracted with diethyl ether (30 mL). The aqueous layer was basified with 5M aqueous sodium hydroxide until pH 14. The solution was extracted with dichloromethane (3 × 30 mL), dried with sodium sulfate, filtered, and concentrated under vacuum to afford the title compound as a yellow oil (1.00 g, 2.3 mmol, 100%). R_f 0.23 (7:3 n-hexane:ethyl acetate). ν_{max} (thin film)/cm^{-1} 3375, 2977, 2935, 2812, 1709, 1511, 1456, 1366, 1246, 1154, 1036, 846, 735, 701. ^{1}H NMR (400 MHz, $CDCl_3$) δ 7.21 (2H, d, *J* = 8.5 Hz, ArH), 6.84 (2H, d, *J* = 8.5 Hz, ArH), 3.79 (3H, s, OCH_3), 3.45 (2H, s, $ArCH_2$), 3.17–3.08 (2H, m, $CONHCH_2$), 2.44 (2H, t, *J* = 6.5 Hz, NCH_2), 2.38 (2H, t, *J* = 7.5 Hz, NCH_2), 2.21 (2H, t, *J* = 7.5 Hz, $COCH_2$), 1.74 (2H, tt, *J* = 7.5, 7.5 Hz, CH_2), 1.61 (2H, tt, *J* = 6.5, 6.5 Hz, CH_2), 1.43 (9H, s, $C(CH_3)_3$), 1.41 (9H, s, $C(CH_3)_3$). ^{13}C NMR (101 MHz, $CDCl_3$) δ 173.2 (CO), 158.7 (ArC), 156.1 (CO), 131.4 (ArC), 130.2 (ArCH), 113.8 (ArCH), 80.2 ($C(CH_3)$), 78.8 ($C(CH_3)$), 58.1 ($ArCH_2$), 55.3 (OCH_3), 52.9 (NCH_2), 52.2 (NCH_2), 39.9 ($NHCH_2$), 33.4 ($COCH_2$), 28.6 ($C(CH_3)_3$), 28.2 ($C(CH_3)_3$), 26.6 (CH_2), 22.5 (NCH_2CH_2). HRMS (ESI$^+$) $C_{24}H_{41}N_2O_5$ (MH^+) theoretical 437.3010; measured 437.3011 (−0.3 ppm error); $C_{24}H_{40}N_2NaO_5$ (MNa^+) theoretical 459.2829; measured 459.2838 (−1.9 ppm error).

tert-Butyl 4-((3-((*tert*-butoxycarbonyl)amino)propyl)(4-methoxyphenyl)amino)butanoate (**11c**)

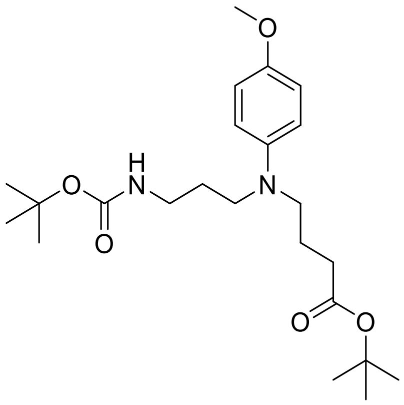

To a stirring solution of amine **10c** (1.00 g, 3.6 mmol) in diisopropyl ethylamine (1.24 mL), *tert*-butyl-4-bromobutryate (0.95 mL, 5.4 mmol) was added and stirred at 70 °C for 72 hours. The reaction was diluted with water (50 mL), the aqueous phase extracted with ethyl acetate (3 × 50 mL), the combined organic phase dried with sodium sulfate, filtered, and concentrated under vacuum and purified via flash column chromatography (7:3 n-hexane:ethyl acetate) to afford the title compound as a yellow oil (1.47 g, 3.48 mmol, 97%). R_f 0.52 (7:3 n-hexane:ethyl acetate). ν_{max} (thin film)/cm^{-1} 3374, 2976, 2934, 1714, 1512, 1457, 1366, 1245, 1153, 1040, 815. ^{1}H NMR (400 MHz, $CDCl_3$) δ 6.78 (2H, d, *J* = 9.0 Hz, ArH), 6.69 (2H, d, *J* = 9.0 Hz, ArH), 4.85 (1H, br s, NH), 3.71 (3H, s, OCH_3), 3.25–3.06 (6H, m, 2×NCH_2/$NHCH_2$), 2.21 (2H, t, *J* = 7.0 Hz, $COCH_2$), 1.76 (2H, tt, *J* = 7.5, 7.5 Hz, CH_2), 1.68 (2H, tt, *J* = 7.0, 7.0 Hz, CH_2), 1.43–1.40 (18H, m, 2×$C(CH_3)_3$). ^{13}C NMR (101 MHz, $CDCl_3$) δ 172.7 (CO), 156.1 (CO), 152.1 (ArC), 142.9 (ArC), 116.1 (ArC), 114.8 (ArC), 80.3 ($C(CH_3)$), 79.0 ($C(CH_3)$), 55.7 (OCH_3), 51.7 (NCH_2), 49.8

(NCH_2), 38.8 ($NHCH_2$), 32.8 ($COCH_2$), 28.4 ($C(CH_3)_3$), 28.1 ($C(CH_3)_3$), 27.6 (CH_2), 22.6 (CH_2). HRMS (ESI$^+$) $C_{23}H_{39}N_2O_5$ (MH^+) theoretical 423.2853; measured 423.2859 (−1.3 ppm error); $C_{23}H_{38}N_2NaO_5$ (MNa^+) theoretical 445.2673; measured 445.2677 (−0.9 ppm error).

1-Benzyl-1,5-diazonan-6-one (**13a**)

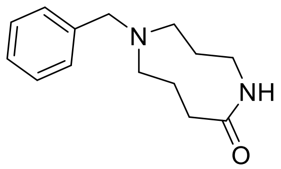

To a stirring solution of carbamate **11a** (999 mg, 2.6 mmol) in dichloromethane (20 mL), hydrochloric acid (12.30 mL, 49.2 mmol, 4N in 1,4-dioxane) was added and stirred at room temperature for 24 hours. The solvent was removed under vacuum via diethyl ether azeotrope. The residue was basified with diisopropyl ethylamine (2.14 mL, 12.3 mmol), redissolved in dichloromethane (49 mL). T3P (1.76 mL, 3.0 mmol, 50 wt% in ethyl acetate) was added to the solution and stirred for 16 hours under argon. The reaction was diluted with water (100 mL), extracted with dichloromethane (3 × 100 mL), the combined organic layers dried with sodium sulfate, filtered, and concentrated under vacuum. The crude was purified via flash column chromatography (dichloromethane 3:2 diethyl ether → ethyl acetate 9:1 methanol) to afford the title compound as a white crystalline solid (494 mg, 2.1 mmol, 87%). Mp 63 °C. R_f 0.32 (19:1 ethyl acetate:triethyl amine). ν_{max} (thin film)/cm^{-1} 3284, 3206, 2931, 2796, 2229, 1646, 1452, 1373, 1340, 1291, 1134, 908, 723, 698. ^{1}H NMR (400 MHz, $CDCl_3$) δ 7.30–7.14 (5H, m, ArH), 6.78–6.68 (1H, m, NH), 3.54 (2H, s, $PhCH_2$), 3.58–3.43 (2H, m, $NHCH_2$), 2.56–2.38 (6H, m, 2×NCH_2/$COCH_2$), 1.79 (2H, tt, J = 6.5, 6.5 Hz, CH_2), 1.44 (2H, tt, J = 5.5, 5.5 Hz, CH_2). ^{13}C NMR (101 MHz, $CDCl_3$) δ 178.3 (CO), 139.5 (ArC), 128.8 (ArCH), 128.2 (ArCH), 126.8 (ArCH), 59.4 ($PhCH_2$), 51.6 (NCH_2), 47.9 (NCH_2), 40.1 ($NHCH_2$), 28.6 (CH_2), 27.6 ($COCH_2$), 24.5 (CH_2). HRMS (ESI$^+$) $C_{14}H_{21}N_2O$ (MH^+) theoretical 233.1648; measured 233.1647 (+0.7 ppm error); $C_{14}H_{20}N_2NaO$ (MNa^+) theoretical 255.1468; measured 255.1464 (+1.6 ppm error); $C_{14}H_{20}KN_2O$ (MK^+) theoretical 271.1207; measured 271.1198 (+3.3 ppm error).

1-(4-Methoxybenzyl)-1,5-diazonan-6-one (**13b**)

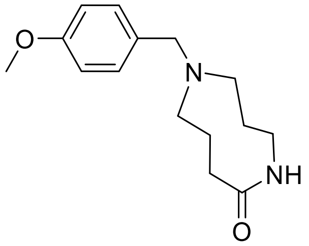

To a stirring solution of carbamate **11b** (1.20 g, 2.7 mmol) in dichloromethane (30 mL), hydrochloric acid (13.7 mL, 55.0 mmol, 4N in 1,4-dioxane) was added and stirred at room temperature for 24 hours. The solvent was removed under vacuum via diethyl ether azeotrope. The residue was basified with diisopropyl ethylamine (2.39 mL, 13.7 mmol), redissolved in dichloromethane (55 mL). T3P (1.96 mL, 3.3 mmol, 50 wt% in ethyl acetate) was added to the solution and stirred for 16 hours under argon. The reaction was diluted with water (50 mL), extracted with dichloromethane (3 × 50 mL), the combined organic layers dried with sodium sulfate, filtered, and concentrated under vacuum. The crude was purified via flash column chromatography (3:2 dichloromethane:diethyl ether → 9:1 ethyl acetate:methanol) to afford the title compound as a colourless oil (567 mg, 2.2 mmol, 79%). R_f 0.54 (9:1 ethyl acetate:methanol). ν_{max} (thin film)/cm^{-1} 3286, 2933, 2834, 1649, 1611, 1511, 1458, 1373, 1340, 1301, 1244, 1178, 1135, 1034, 834, 816. ^{1}H NMR (400 MHz, $CDCl_3$) δ 7.17 (2H, d, *J* = 8.5 Hz, ArH), 6.82 (2H, d, *J* = 8.5 Hz, ArH), 6.34–6.25 (1H, m, NH), 3.76 (3H, s, OCH_3), 3.50 (2H, s, $ArCH_2$), 2.58–2.38 (6H, m, $NHCH_2$), 2.56–2.38 (6H, m, 2×NCH_2/$COCH_2$), 1.81 (2H, tt, *J* = 6.5, 6.5 Hz, CH_2), 1.44 (2H, tt, *J* = 5.5, 5.5 Hz, CH_2). ^{13}C NMR (101 MHz, $CDCl_3$) δ 178.4 (CO), 158.6 (ArC), 131.7 (ArC), 130.1 (ArCH), 113.6 (ArCH), 58.9 ($ArCH_2$), 55.2 (OCH_3), 51.8 (NCH_2), 48.1 (NCH_2), 40.3 ($NHCH_2$), 28.7 (CH_2), 27.7 ($COCH_2$), 24.7 (CH_2). HRMS (ESI$^+$) $C_{15}H_{23}N_2O_2$ (MH^+) theoretical 263.1754; measured 263.1751 (+1.0 ppm error); $C_{15}H_{22}N_2NaO_2$ (MNa^+) theoretical 285.1573; measured 285.1571 (+1.0 ppm error); $C_{15}H_{22}KN_2O_2$ (MK^+) theoretical 301.1313; measured 301.1307 (+1.8 ppm error).

1-(4-Methoxyphenyl)-1,5-diazonan-6-one (**13c**)

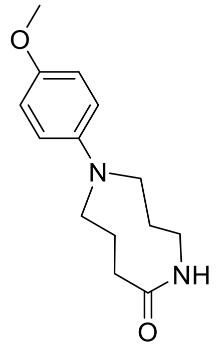

To a stirring solution of carbamate **11c** (1.16 g, 2.8 mmol) in dichloromethane (25 mL), hydrochloric acid (13.8 mL, 55.2 mmol, 4N in 1,4-dioxane) was added and stirred at room temperature for 24 hours. The solvent was removed under vacuum via diethyl ether azeotrope. The residue was basified with diisopropyl ethylamine (2.40 mL, 13.8 mmol), redissolved in dichloromethane (55 mL). T3P (2.00 mL, 3.36 mmol, 50 wt% in ethyl acetate) was added to the solution and stirred for 16 hours under argon. The reaction was diluted with water (50 mL), extracted with dichloromethane (3 × 50 mL), the combined organic layers dried with sodium sulfate, filtered, and concentrated under vacuum. The crude was purified via flash column chromatography (ethyl acetate → 9:1 ethyl acetate:methanol) to afford the title compound as a white crystalline solid (417 mg, 1.68 mmol, 61%). Mp 133 °C. R_f 0.54 (9:1 ethyl acetate:methanol). ν_{max} (thin film)/cm^{-1} 3315, 2915, 2865, 1651, 1510, 1441, 1337, 1239, 1153, 1041, 804, 767, 666, 520. ^{1}H NMR (400 MHz, $CDCl_3$) δ 6.86–6.79 (2H, m, ArH), 6.68–6.61 (2H, m, ArH), 5.59–5.41 (1H, m, NH), 3.75 (3H, s, OCH_3), 3.52–3.44 (2H, m, $NHCH_2$), 3.42 (2H, t, *J* = 6.0 Hz, NCH_2), 3.35 (2H, t, *J* = 6.5 Hz, NCH_2), 2.47–2.40 (2H, m, $COCH_2$), 2.15 (2H, tt, *J* = 6.5, 6.5 Hz, CH_2), 1.85 (2H, tt, *J* = 6.0, 6.0 Hz, CH_2). ^{13}C NMR (101 MHz, $CDCl_3$) δ 176.4 (CO), 152.5 (ArCO), 143.5 (ArC), 115.0 (ArCH), 113.3 (ArCH), 55.9 (OCH_3), 55.5 (NCH_2), 53.4 (NCH_2), 40.5 ($NHCH_2$), 29.0 (CH_2), 27.1 ($COCH_2$), 26.4 (CH_2). HRMS (ESI$^+$) $C_{14}H_{21}N_2O_2$ (MH^+) theoretical 249.1598; measured 249.1574 (+9.6 ppm error); $C_{14}H_{20}N_2NaO_2$ (MNa^+) theoretical 271.1417; measured 271.1415 (+0.9 ppm error); $C_{14}H_{20}KN_2O_2$ (MK^+) theoretical 287.1156; measured 287.1132 (+8.6 ppm error).

5-Acryloyl-1-benzyl-1,5-diazonan-6-one (**14**)

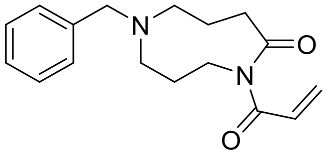

To a stirring solution of lactam **13a** (200 mg, 0.86 mmol) in dry THF (8.50 mL), lithium hexamethyldisilylazane (0.95 mL, 0.95 mmol, 1M solution in THF) was added and stirred at 0 °C for 1 hour. Acryloyl chloride (90 µL, 1.1 mmol) was added to the reaction mixture at 0 °C, warmed to room

temperature and stirred for 1 hour. The reaction was quenched with water (20 mL), the aqueous phase was extracted with dichloromethane (3 × 20 mL). The combined organic layers dried with sodium sulfate, filtered, and concentrated under vacuum. The crude was purified via flash column chromatography (4:1 n-hexane:ethyl acetate) to afford the title compound as a yellow oil (147 mg, 0.51 mmol, 60%). R_f 0.29 (4:1 n-hexane:ethyl acetate). ν_{max} (thin film)/cm^{-1} 2927, 2816, 2249, 1665, 1617, 1453, 1408, 1372, 1229, 1174, 1139, 1102, 1066, 958, 911, 794, 726, 700. ^{1}H NMR (400 MHz, $CDCl_3$) δ 7.37–7.16 (5H, m, PhH), 6.63 (1H, dd, *J* = 16.5, 10.0 Hz, CH), 6.39 (1H, dd, *J* = 16.5, 2.0 Hz, $CHCH_2$), 5.73 (1H, dd, *J* = 10.0, 2.0 Hz, $CHCH_2$), 3.89 (2H, t, *J* = 6.0 Hz, NCH_2), 3.48 (2H, s, $PhCH_2$), 2.78–2.68 (2H, m, $COCH_2$), 2.60 (2H, t, *J* = 6.0 Hz, NCH_2), 2.55 (2H, t, *J* = 6.0 Hz, NCH_2), 1.88 (2H, tt, *J* = 6.0, 6.0 Hz, CH_2), 1.67 (2H, tt, *J* = 6.0, 6.0 Hz, CH_2). ^{13}C NMR (101 MHz, $CDCl_3$) δ 179.7 (CO), 167.8 (CO), 138.1 (PhC), 130.5 (CH), 129.6 (PhC), 128.4 ($CHCH_2$), 128.3 (PhC), 127.2 (PhC), 57.8 ($PhCH_2$), 53.8 (NCH_2), 50.1 (NCH_2), 43.3 (NCH_2), 37.1 ($COCH_2$), 25.8 (CH_2), 25.3 (CH_2). HRMS (ESI^+) $C_{17}H_{23}N_2O_2$ (MH^+) theoretical 287.1754; measured 287.1764 (−3.5 ppm error); $C_{17}H_{22}N_2NaO_2$ (MNa^+) theoretical 309.1573; measured 309.1567 (+2.1 ppm error); $C_{17}H_{22}KN_2O_2$ (MK^+) theoretical 325.1313; measured 325.1307 (+2.0 ppm error).

1,9-Dibenzyl-1,5,9-triazacyclotridecane-4,13-dione (**16a**)

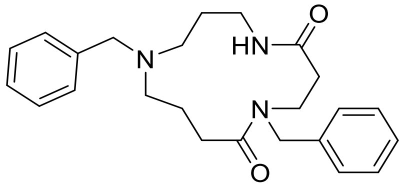

Synthesised as described in general procedure A using imide **14** (52.2 mg, 0.18 mmol) and benzylamine (21 µL, 0.19 mmol). Purified via flash column chromatography (9:1 ethyl acetate:methanol) to afford colourless oil (60.7 mg, 0.15 mmol, 85%). R_f 0.28 (4:1 n-hexane:ethyl acetate). ν_{max} (thin film)/cm^{-1} 3299, 2926, 2854, 1626, 1452, 1359, 1145, 1029, 861, 730, 699. Note: both the ^{1}H and ^{13}C NMR spectra obtained for this product showed severe broadening of their NMR signals due to rotamers. ^{1}H NMR (400 MHz, d_6-DMSO) δ 7.96 (1H, s (br), NH), 7.41–7.36 (2H, m, PhH), 7.34–7.29 (4H, m, PhH), 7.26–7.22 (2H, m, PhH), 7.22–7.15 (2H, m, PhH), 4.52 (2H, s, $PhCH_2$), 3.55–3.47 (2H, m, NCH_2), 3.45 (2H, s, $PhCH_2$), 3.25–3.14 (2H, m, NCH_2), 2.46–2.33 (6H, m, CH_2), 2.32–2.26 (2H, m, CH_2), 1.70–1.63 (2H, m, CH_2), 1.49–1.42 (2H, m, CH_2). ^{13}C NMR (101 MHz, $CDCl_3$) δ 172.7 (CO), 171.4 (CO), 140.1 (PhC), 138.5 (PhC), 129.3 (PhCH), 128.8 (PhCH), 128.5 (PhCH), 128.1 (PhCH), 127.2 (PhCH), 126.9 (PhCH), 58.3 ($PhCH_2$), 53.1 (NCH_2), 51.4 (NCH_2), 47.2 ($PhCH_2$), 43.6 (NCH_2), 36.0 (NCH_2), 35.7 (CH_2), 28.9 (CH_2), 25.9 (CH_2), 23.9 (CH_2). HRMS (ESI^+) $C_{24}H_{32}N_3O_2$ (MH^+) theoretical 394.2489; measured 394.2496 (−1.8 ppm error).

9-Benzyl-1-(4-fluorobenzyl)-1,5,9-triazacyclotridecane-4,13-dione (**16b**)

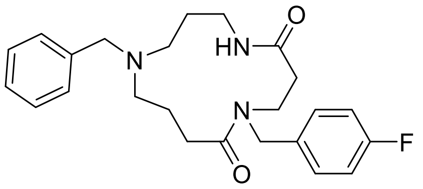

Synthesised as described in general procedure A using imide **14** (64.2 mg, 0.22 mmol) and 4-fluorobenzylamine (27 µL, 0.24 mmol). Purified via flash column chromatography (1:1 n-hexane:ethyl acetate) to afford colourless crystalline solid (78.9 mg, 0.19 mmol, 86%). Mp 83 °C. R_f 0.26 (1:1 n-hexane:ethyl acetate). ν_{max} (thin film)/cm^{-1} 3286, 2932, 1639, 1509, 1452, 1222, 700. Note: both the ^{1}H and ^{13}C NMR spectra obtained for this product showed severe broadening of their NMR signals due to rotamers (2:1 ratio). Only the 13C NMR of the major rotamer is reported. ^{1}H NMR (700 MHz, d_6-DMSO) δ 7.98–7.93 (1H, m, NH, major rotamer), 7.92–7.87 (1H, m, NH, minor rotamer), 7.39–7.36 (2H, m, ArH, major rotamer), 7.35–7.29 (2H, m, ArH, both rotamers), 7.28–7.19 (4H, m, ArH, both rotamers), 7.16–7.11 (2H, m, ArH, major rotamer), 4.49 (2H, s, $ArCH_2$, both rotamers), 3.53–3.47 (2H, m, CH_2, both rotamers), 3.45 (2H, s, $PhCH_2$, both rotamers), 3.29–3.15 (2H, m, CH_2, both rotamers), 2.43–2.39 (2H, m, CH_2, both rotamers), 2.39–2.34 (2H, m, CH_2, both rotamers), 2.31–2.26 (2H, m, CH_2, both rotamers), 1.72–1.59 (2H, m, CH_2, both rotamers), 1.51–1.38 (2H, m, CH_2, both rotamers). ^{13}C NMR (176 MHz, d_6-DMSO) δ 172.8 (CO), 171.4 (CO), 162.4 (d, *J* = 242.5 Hz, ArCF), 140.1 (ArC), 134.7 (d, *J* = 3.0 Hz, ArCH), 130.1 (d, *J* = 8.0 Hz, ArCH), 129.3 (PhCH), 128.5 (PhCH), 127.2 (PhCH), 115.6 (d, *J* = 21.0 Hz, ArC), 58.4 ($ArCH_2$), 53.1 (NCH_2), 51.4 (NCH_2), 46.6 ($PhCH_2$), 43.6 (NCH_2), 36.0 (NCH_2), 35.8 (CH_2), 28.9 (CH_2), 25.9 (CH_2), 23.9 (CH_2). ^{19}F NMR (656 MHz, d_6-DMSO) δ −115.7 (CF, minor rotamer), −116.0 (CF, major rotamer). HRMS (ESI$^+$) $C_{24}H_{31}FN_3O_2$ (MH^+) theoretical 412.2395; measured 412.2398 (−0.9 ppm error).

9-Benzyl-1-cyclopropyl-1,5,9-triazacyclotridecane-4,13-dione (**16c**)

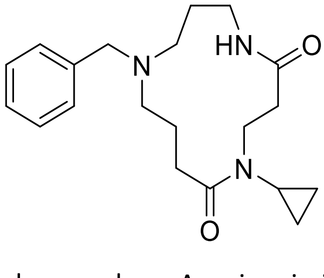

Synthesised as described in general procedure A using imide **14** (53.9 mg, 0.19 mmol) and cyclopropylamine (15 µL, 0.22 mmol). Purified via flash column chromatography (19:1 ethyl acetate:methanol) to afford colourless oil (30.4 mg, 95 µmol, 51%). R_f 0.28 (4:1 n-hexane:ethyl acetate). ν_{max} (thin film)/cm^{-1} 3297, 2926, 1648, 1543, 1452, 1370, 1273, 737, 701. Note: the ^{1}H NMR spectra obtained for this product showed severe broadening of their NMR signals due to rotamers. ^{1}H

NMR (400 MHz, $CDCl_3$) δ 7.60–7.03 (5H, m, PhH), 4.43–3.62 (1H, m, CH_2), 3.62–3.09 (3H, m, CH_2), 3.05–2.73 (2H, m, CH_2), 2.73–1.91 (9H, m, CH/CH_2), 1.89–1.45 (3H, m, CH_2), 1.39–1.14 (1H, m, CH_2), 1.06–0.80 (2H, m, CH_2), 0.78–0.56 (2H, m, CH_2). ^{13}C NMR (101 MHz, $CDCl_3$) δ 176.0 (CO), 171.4 (CO), 137.4 (PhC), 130.4 (PhCH), 128.4 (PhCH), 127.5 (PhCH), 57.9 (NCH_2), 55.9 (NCH_2), 54.1 (NCH_2), 45.4 (NCH_2), 40.8 (NCH_2), 35.0 (CH_2), 33.8 (CH_2), 31.7 (CH), 29.8 (CH_2), 24.5 (CH_2), 21.3 (CH_2). HRMS (ESI^+) $C_{20}H_{30}N_3O_2$ (MH^+) theoretical 344.2333; measured 344.2336 (−1.0 ppm error); $C_{20}H_{29}N_3NaO_2$ (MNa^+) theoretical 366.2152; measured 366.2187 (−9.5 ppm error).

1-Allyl-9-benzyl-1,5,9-triazacyclotridecane-4,13-dione (**16d**)

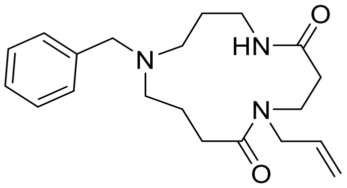

Synthesised as described in general procedure A using imide **14** (49.0 mg, 0.17 mmol) and allylamine (15 µL, 0.20 mmol). Purified via flash column chromatography (9:1 ethyl acetate:methanol) to afford colourless oil (51.7 mg, 0.15 mmol, 88%). R_f 0.17 (4:1 n-hexane:ethyl acetate). ν_{max} (thin film)/cm^{-1} 3293, 2929, 1637, 1548, 1451, 1358, 1234, 732, 700. Note: both the ^1H and ^{13}C NMR spectra obtained for this product showed severe broadening of their NMR signals due to rotamers (1:4 ratio). ^1H NMR (400 MHz, $CDCl_3$) δ 7.37–7.17 (5H, m, PhH, both rotamers), 5.78–5.56 (1H, m, CH, both rotamers), 5.14 (1H, d, *J* = 11.0 Hz, $CHCH_2$, both rotamers), 5.07 (1H, d, *J* = 17.5 Hz, $CHCH_2$, both rotamers), 4.11–3.87 (1H, m, CH_2, both rotamers), 3.87–3.66 (1H, m, CH_2, both rotamers), 3.63–3.51 (1H, m, CH_2, both rotamers), 3.49–3.40 (1H, m, CH_2, both rotamers), 3.40–3.24 (1H, m, CH_2, both rotamers), 3.24–3.11 (1H, m, CH_2, both rotamers), 3.11–2.70 (2H, m, CH_2, both rotamers), 2.66–2.06 (8H, m, CH_2, both rotamers), 1.95–1.72 (1H, m, CH_2, both rotamers), 1.72–1.46 (3H, m, CH_2, both rotamers). ^{13}C NMR (101 MHz, $CDCl_3$) δ 174.2 (CO, major rotamer), 172.1 (CO, minor rotamer), 171.1 (CO, major rotamer), 169.8 (CO, minor rotamer), 138.5 (PhC, minor rotamer), 137.5 (PhC, major rotamer), 133.2 (CH, minor rotamer), 132.7 (CH, major rotamer), 130.2 (PhCH, major rotamer), 129.5 (PhCH, minor rotamer), 128.5 (PhCH, minor rotamer), 128.4 (PhCH, major rotamer), 127.4 (PhCH, both rotamers), 117.5 ($CHCH_2$, minor rotamer), 116.8 ($CHCH_2$, major rotamer), 59.1 (CH_2, minor rotamer), 58.1 (CH_2, major rotamer), 55.6 (CH_2, major rotamer), 54.9 (CH_2, minor rotamer), 53.7 (CH_2, both rotamers), 52.3 (CH_2, both rotamers), 44.6 (CH_2, both rotamers), 40.5 (CH_2, both rotamers), 36.2 (CH_2, minor rotamer), 35.2 (CH_2, major rotamer), 32.1 (CH_2, both rotamers), 24.9 (CH_2, minor rotamer), 24.7 (CH_2, major rotamer), 22.2 (CH_2, major rotamer), 21.9 (CH_2, minor rotamer). HRMS (ESI^+) $C_{20}H_{30}N_3O_2$ (MH^+) theoretical 344.2333; measured 344.2331 (+0.4 ppm error).

9-Benzyl-1-(prop-2-yn-1-yl)-1,5,9-triazacyclotridecane-4,13-dione (**16e**)

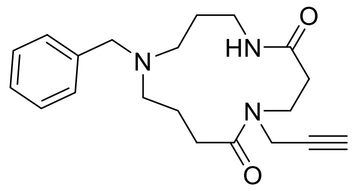

Synthesised as described in general procedure A using imide **14** (43.6 mg, 0.15 mmol) and propagylamine (11 µL, 0.17 mmol). Purified via flash column chromatography (19:1 ethyl acetate:methanol to afford colourless crystalline solid (31.0 mg, 91 µmol, 60%). Mp 72 °C. R_f 0.40 (9:1 ethyl acetate:methanol). ν_{max} (thin film)/cm^{-1} 3289, 2930, 1642, 1550, 1449, 1358, 1233, 700. ^{1}H NMR (400 MHz, d_6-DMSO) δ 7.89 (1H, s (br), NH), 7.39–7.28 (4H, m, PhH), 7.27–7.20 (1H, m, PhH), 4.16 (2H, s, $PhCH_2$), 3.73–3.63 (2H, m, CH_2), 3.44 (2H, s, CCH_2), 3.22–3.16 (2H, m, CH_2), 3.16–3.12 (1H, t, *J* = 2.0 Hz, CCH), 2.40–2.25 (8H, m, CH_2), 1.69–1.58 (2H, m, CH_2), 1.46–1.37 (2H, m, CH_2). ^{13}C NMR (176 MHz, d_6-DMSO) δ 172.2 (CO), 171.3 (CO), 140.1 (PhC), 129.3 (PhCH), 128.5 (PhCH), 127.2 (PhCH), 80.6 (CCH), 74.3 (NCH_2), 58.4 ($PhCH_2$), 53.0 (NCH_2), 51.6 (NCH_2), 43.6 (NCH), 36.0 (NCH_2), 35.7 (CH_2), 33.3 (CH_2), 28.8 (CH_2), 26.0 (CH_2), 23.7 (CH_2). HRMS (ESI$^+$) $C_{20}H_{28}N_3O_2$ (MH^+) theoretical 342.2176; measured 342.2171 (+1.4 ppm error); $C_{20}H_{27}N_3NaO_2$ (MNa^+) theoretical 364.1995; measured 364.1991 (+1.2 ppm error).

1-(Benzo[d][1,3]dioxol-5-ylmethyl)-9-benzyl-1,5,9-triazacyclotridecane-4,13-dione (**16f**)

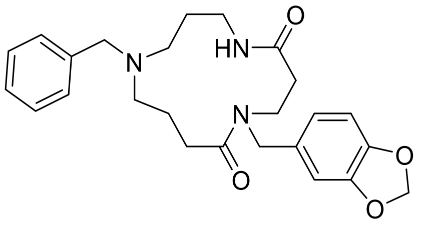

Synthesised as described in general procedure A using imide **14** (51.0 mg, 0.18 mmol) and piperonylamine (24 µL, 0.19 mmol). Purified via flash column chromatography (ethyl acetate → 19:1 ethyl acetate:methanol) to afford colourless oil (60.7 mg, 0.14 mmol, 78%). R_f 0.28 (4:1 n-hexane:ethyl acetate). ν_{max} (thin film)/cm^{-1} 3295, 2932, 1638, 1490, 1443, 1243, 1038, 924, 731, 700. Note: both the ^{1}H and ^{13}C NMR spectra obtained for this product showed severe broadening of their NMR signals due to rotamers (1:3 ratio). Only the 13C NMR of the major rotamer is reported. ^{1}H NMR (700 MHz, d_6-DMSO) δ 7.97–7.91 (1H, m, NH, major rotamer), 7.91–7.84 (1H, m, NH, minor rotamer), 7.40–7.27 (4H, m, PhCH, both rotamers), 7.26–7.20 (1H, m, PhCH, both rotamers), 6.90 (1H, d, *J* = 8.0 Hz, ArCH, minor rotamer), 6.84 (1H, d, *J* = 8.0 Hz, ArCH, major rotamer), 6.78 (1H, s, ArCH, major rotamer), 6.73–6.70 (1H, m, ArCH, both rotamers), 6.64–6.62 (1H, d, *J* = 8.0 Hz, ArCH, minor rotamer), 6.01 (2H, s, OCH_2, minor rotamer), 5.98 (2H, s, OCH_2, major rotamer), 4.41 (2H, s, $ArCH_2$, major rotamer), 4.16–

4.11 (2H, m, $ArCH_2$, minor rotamer), 3.50 (2H, s, $ArCH_2$, major rotamer), 3.50–3.46 (2H, m, CH_2, major rotamer), 3.45 (2H, s, $ArCH_2$, major rotamer), 3.24–3.18 (2H, m, CH_2, major rotamer), 3.14–3.09 (2H, m, CH_2, minor rotamer), 2.45–2.31 (8H, m, CH_2, both rotamers), 2.31–2.25 (2H, m, CH_2, both rotamers), 1.69–1.61 (2H, m, CH_2, major rotamer), 1.60–1.53 (4H, m, CH_2, minor rotamer), 1.50–1.38 (2H, m, CH_2, major rotamer). ^{13}C NMR (176 MHz, d_6-DMSO, major rotamer) δ 172.7 (CO), 171.4 (CO), 147.8 (ArC), 146.7 (ArC), 140.1 (ArC), 132.4 (ArC), 129.3 (PhCH), 128.5 (PhCH), 127.2 ($PHCH_2$), 121.6 (ArCH), 108.8 (ArCH), 108.5 (ArCH), 101.3 (OCH_2), 58.4 (CH_2), 53.1 (CH_2), 51.4 (CH_2), 46.9 (CH_2), 43.3 (CH_2), 36.0 (CH_2), 35.7 (CH_2), 28.9 (CH_2), 25.9 (CH_2), 23.9 (CH_2). HRMS (ESI^+) $C_{25}H_{32}N_3O_4$ (MH^+) theoretical 438.2387; measured 438.2393 (−1.4 ppm error).

9-Benzyl-1-(2-(methylthio)ethyl)-1,5,9-triazacyclotridecane-4,13-dione (**16g**)

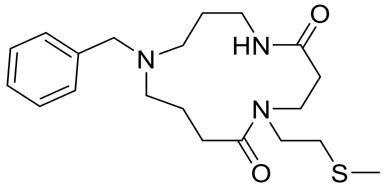

Synthesised as described in general procedure A using imide **14** (50.1 mg, 0.17 mmol) and 2-(methylthio)ethylamine (18 µL, 0.19 mmol). Purified via flash column chromatography (9:1 ethyl acetate:methanol) to afford yellow oil (37.3 mg, 99 µmol, 56%). R_f 0.21 (4:1 n-hexane:ethyl acetate). ν_{max} (thin film)/cm^{-1} 3295, 2927, 1641, 1548, 1452. Note: both the ^1H and ^{13}C NMR spectra obtained for this product showed severe broadening of their NMR signals due to rotamers (1:2 ratio). Only the 13C NMR of the major rotamer is reported. ^1H NMR (700 MHz, d_6-DMSO) δ 7.94–7.89 (1H, s (br), NH, major rotamer), 7.83–7.75 (1H, s (br), NH, minor rotamer), 7.39–7.29 (4H, m, PhCH, both rotamers), 7.25–7.20 (1H, m, PhCH, both rotamers), 3.66–3.56 (2H, m, $PhCH_2$, both rotamers), 3.47–3.41 (1H, m, NCH_2, major rotamer), 3.41–3.37 (1H, m, NCH_2, minor rotamer), 3.22–3.15 (2H, m, CH_2, both rotamers), 2.65–2.60 (2H, m, CH_2, minor rotamer), 2.60–2.55 (2H, m, CH_2, major rotamer), 2.42–2.23 (9H, m, CH_2, both rotamers), 2.12–2.06 (3H, m, CH_3, both rotamers), 1.69–1.58 (2H, m, CH_2, both rotamers), 1.44–1.34 (2H, m, CH_2, both rotamers). ^{13}C NMR (176 MHz, d_6-DMSO, major rotamer) δ 172.4 (CO), 171.4 (CO), 129.3 (PhCH), 128.6 (PHC), 128.5 (PhCH), 127.2 (PhCH), 58.4 ($PhCH_2$), 53.1 (NCH_2), 45.1 (NCH_2), 44.8 (NCH_2), 40.5 (NCH_2), 36.4 (NCH_2), 36.0 (CH_2), 30.8 (CH_2), 28.8 (CH_2), 26.0 (CH_2), 23.8 (CH_2), 15.1 (CH_3). HRMS (ESI^+) $C_{20}H_{32}N_3O_2S$ (MH^+) theoretical 378.2210; measured 378.2210 (+0.0 ppm error); $C_{20}H_{31}N_3NaO_2S$ (MNa^+) theoretical 400.2029; measured 400.2028 (+0.3 ppm error).

3-(((Benzyloxy)carbonyl)amino)-3-phenylpropanoic acid (S1)

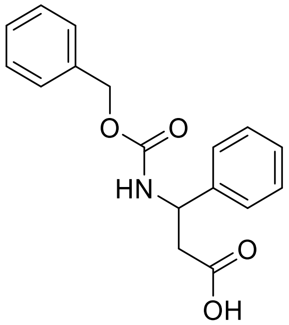

To a stirring solution of β-phenylalanine (10.0 g, 61 mmol) and sodium hydroxide (7.21 g, 180 mmol) in water (100 mL) was added a solution of CbzCl (17.3 mL, 121 mmol) in acetone (100 mL) dropwise at 0 °C and stirred for 2 hours. The reaction was acidified to pH 1 with 2M HCl (aq), extracted with ethyl acetate (3 × 100 mL). The combined organic layers dried with sodium sulfate, filtered and concentrated under vacuum. The crude was purified via flash column chromatography (3:2 n-hexane:ethyl acetate) to afford the title compound as a white solid (12.9 g, 43.3 mmol, 71%). Mp 97 °C. R_f 0.14 (3:2 n-hexane:ethyl acetate). ν_{max} (thin film)/cm^{-1} 3360, 3037, 1690, 1526, 1433, 1287, 1251, 1228, 1086, 1026, 971, 913, 754, 967, 614, 582, 488. ^{1}H NMR (400 MHz, $CDCl_3$) δ 7.90 (1H, d, *J* = 8.5 Hz, NH), 7.39–7.13 (10H, m, PhH), 4.99–4.92 (2H, m, $PhCH_2$), 4.92–4.86 (1H, m, PhCH), 2.73–2.52 (2H, m, $COCH_2$). ^{13}C NMR (101 MHz, $CDCl_3$) δ 172.2 (COOH), 155.9 (CO), 143.4 (PhC), 137.5 (PhC), 128.9 (PhCH), 128.8 (PhCH), 128.3 (PhCH), 128.3 (PhCH), 127.6 (PhCH), 126.9 (PhCH), 65.8 ($PhCH_2$), 52.2 (PhCH), 41.7 ($COCH_2$). HRMS (ESI$^-$) $C_{17}H_{16}NO_4$ (M−H$^+$) theoretical 298.1085; measured 298.1101 (−5.5 ppm error).

3-((((9H-Fluoren-9-yl)methoxy)carbonyl)amino)-3-phenylpropanoic acid (S2) – racemic and *R*-enantiomer

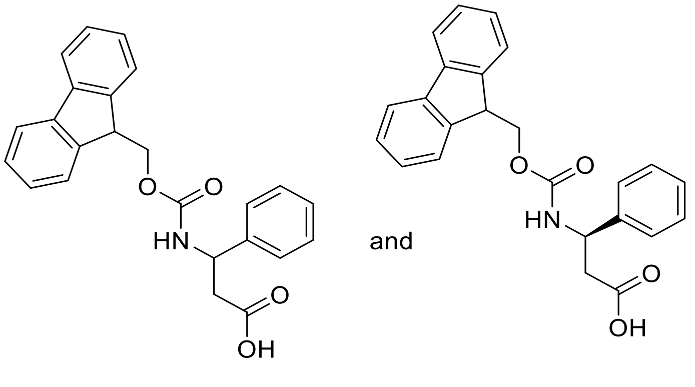

Racemic S2

Synthesised as described in general procedure B using β-phenylalanine (5.00 g, 30.3 mmol). Purified via flash column chromatography (9:1 n-hexane:ethyl acetate) to afford the title compound as a white solid (10.5 g, 27.0 mmol, 89%). Mp 167 °C. R_f 0.29 (4:1 n-hexane:ethyl acetate). ν_{max} (thin film)/cm^{-1}

3363, 3034, 1703, 1529, 1432, 1412, 1285, 1259, 1228, 1190, 1138, 1026, 958, 757, 739, 700, 622, 546, 492. ^{1}H NMR (400 MHz, $CDCl_3$) δ 7.92 (1H, d, *J* = 8.5 Hz, NH), 7.84 (2H, d, *J* = 7.5 Hz, ArH), 7.63 (2H, d, *J* = 7.5 Hz, ArH), 7.40–7.32 (2H, m, ArH), 7.32–7.14 (7H, m, ArH), 4.89 (1H, td, *J* = 8.5, 6.0 Hz, PhCH), 4.30–4.09 (3H, m, OCH_2/OCH_2CH), 2.73–2.54 (2H, m, $COCH_2$). ^{13}C NMR (101 MHz, $CDCl_3$) δ 172.3 (COOH), 155.9 (CO), 144.5 (ArC), 144.3 (ArC), 143.4 (ArC), 141.2 (ArCH), 128.8 (ArCH), 128.1 (ArCH), 127.6 (ArCH), 126.9 (ArCH), 125.7 (ArCH), 120.7 (ArCH), 65.9 (OCH_2), 52.2 (PhCH), 47.2 (CH_2CH), 41.6 ($COCH_2$). HRMS (APCI$^+$) $C_{24}H_{22}NO_4$ (MH^+) theoretical 388.1543; measured 388.1530 (−3.4 ppm error).

(*R*)-S2

Synthesised as above, starting from (*R*)-β-phenylalanine. All spectroscopic data were the same as above, with the exception of: $[\alpha]_D^{20}$ +18.63 (c = 1.0).

3-((((9H-Fluoren-9-yl)methoxy)carbonyl)amino)butanoic acid (**S3**)

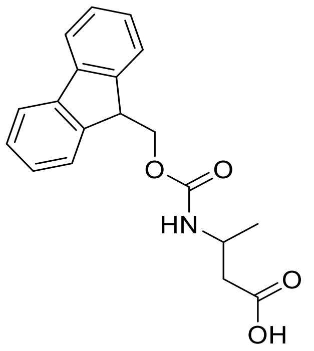

Synthesised as described in general procedure B using 3-aminobutanoic acid (2.00 g, 19.4 mmol). Purified via flash column chromatography (ethyl acetate → 9:1 ethyl acetate:methanol) to afford the title compound as a white solid (5.85 g, 18.0 mmol, 93%). Mp 107 °C. R_f 0.57 (9:1 ethyl acetate:methanol). ν_{max} (thin film)/cm^{-1} 3317, 2977, 1688, 1535, 1449, 1261, 1102, 1054, 937, 759, 737, 621. ^{1}H NMR (400 MHz, $CDCl_3$) δ 7.88 (2H, d, *J* = 7.5 Hz, ArH), 7.69–7.60 (2H, m, ArH), 7.37 (2H, t, *J* = 7.5 Hz, ArH), 7.33–7.21 (2H, m, ArH), 4.34–4.19 (2H, m, OCH_2), 4.16 (1H, t, *J* = 7.0 Hz, CH_2CH), 3.90–3.74 (1H, m, NCH), 2.45–2.32 (1H, m, $COCH_2$), 2.29–2.14 (1H, m, $COCH_2$), 1.04 (3H, d, *J* = 6.5 Hz, CH_3). ^{13}C NMR (101 MHz, $CDCl_3$) δ 173.0 (COOH), 155.8 (CO), 144.5 (PhC), 141.3 (PhC), 128.1 (ArCH), 127.6 (ArCH), 125.7 (ArCH), 120.6 (ArCH), 65.7 (OCH_2), 47.3 (CH_2CH), 44.3 (NCH), 41.4 ($COCH_2$), 21.0 (CH_3). HRMS (ESI$^+$) $C_{19}H_{19}NNaO_4$ (MNa^+) theoretical 348.1206; measured 348.1214 (−2.1 ppm error).

3-((((9H-Fluoren-9-yl)methoxy)carbonyl)(methyl)amino)propanoic acid (**S4**)

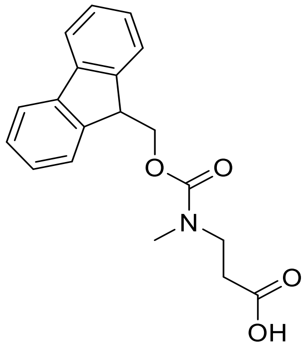

Synthesised as described in general procedure B using *N*-methyl-β-alanine (1.05 g, 10.2 mmol). Purified via flash column chromatography (ethyl acetate → 9:1 ethyl acetate:methanol) to afford the title compound as a white solid (1.56 g, 4.79 mmol, 47%). Mp 85 °C. R_f 0.54 (9:1 ethyl acetate:methanol). ν_{max} (thin film)/cm^{-1} 3059, 1725, 1655, 1493, 1446, 1415,1364, 1299, 1228, 1180, 1134, 1071, 1057, 1035, 966, 857, 756, 736, 611, 532. Note: both the ^{1}H and ^{13}C NMR spectra obtained for this product showed severe broadening of their NMR signals due to rotamers. ^{1}H NMR (400 MHz, $CDCl_3$) δ 7.90–7.75 (2H, m, ArH), 7.59 (2H, d, *J* = 7.5 Hz, ArH), 7.36 (2H, t, *J* = 7.5 Hz, ArH), 7.28 (2H, t, *J* = 7.5 Hz, ArH), 4.36–4.25 (2H, m, OCH_2), 4.25–4.19 (1H, m, CH), 3.35 (1H, t, *J* = 7.5 Hz, NCH_2), 3.25 (1H, t, *J* = 7.5 Hz, NCH_2), 2.75 (3H, s, NCH_3), 2.37 (1H, t, *J* = 7.0 Hz, $COCH_2$), 2.19 (1H, t, *J* = 7.0 Hz, $COCH_2$). ^{13}C NMR (101 MHz, $CDCl_3$) δ 173.4 (COOH), 155.7 (CO), 144.4 (ArC), 141.3 (ArC), 128.2 (ArC), 127.6 (ArC), 125.5 (ArC), 120.6 (ArC), 67.1 (OCH_2), 47.3 (CH), 45.4/44.8 (NCH_2), 35.0/34.5 (CH_3), 33.1/32.8 ($COCH_2$). HRMS (ESI$^+$) $C_{19}H_{19}NNaO_4$ (MNa^+) theoretical 348.1206; measured 348.1209 (−0.7 ppm error).

Benzyl (3-(5-benzyl-9-oxo-1,5-diazonan-1-yl)-3-oxo-1-phenylpropyl)carbamate (**18a**)

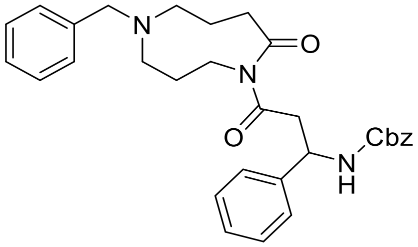

To a stirring solution of lactam **13a** (3.00 g, 12.9 mmol) in dry THF (129 mL), lithium hexamethyldisilylazane (25.85 mL, 25.9 mmol, 1M solution in THF) was added and stirred at 0 °C for 1 hour. A solution of acid chloride **17a** (19.4 mmol, 1.5 eq. freshly prepared from carboxylic acid **S1** using general procedure C) in THF (20 mL) was added to the reaction mixture at 0 °C and stirred for 1 hour. The reaction was quenched with saturated ammonium chloride (aq) (100 mL), the aqueous phase was extracted with ethyl acetate (3 × 100 mL). The combined organic layers dried with sodium sulfate, filtered, and concentrated under vacuum. The crude was purified via flash column chromatography (4:1 n-hexane:ethyl acetate) to afford the title compound as a yellow oil (3.28 g, 6.4 mmol, 49%). R_f

0.29 (7:3 n-hexane:ethyl acetate). ν_{max} (thin film)/cm^{-1} 3335, 3030, 2931, 1718, 1496, 1454, 1371, 1247, 1182, 1027, 960, 912, 732, 699. Note: both the ^{1}H and ^{13}C NMR spectra obtained for this product showed severe broadening of their NMR signals due to rotamers. ^{1}H NMR (400 MHz, $CDCl_3$) δ 7.50–7.07 (16H, m, ArH/NH), 5.30–5.23 (1H, m, CH), 5.14–4.94 (2H, m, $PhCH_2$), 3.93–3.77 (1H, m, CH_2), 3.75–3.58 (1H, m, CH_2), 3.49–3.35 (2H, m, CH_2), 3.35–3.24 (1H, m, CH_2), 3.12–2.95 (1H, m, CH_2), 2.73–2.34 (5H, m, CH_2), 1.98–1.73 (2H, m, CH_2), 1.67–1.39 (2H, m, CH_2). ^{13}C NMR (101 MHz, $CDCl_3$) δ 178.9 (CO), 173.0 (CO), 155.8 (CO), 142.0 (PhC), 137.7 (PhC), 136.6 (PhC), 129.5 (PhCH), 128.6 (PhCH), 128.5 (PhCH), 128.3 (PhCH), 128.1 (PhCH), 128.0 (PhCH), 127.4 (PhCH), 127.2 (PhCH), 126.3 (PhCH), 66.7 (OCH_2), 56.3 (NCH_2), 53.4 (CH_2), 52.8 (CH), 50.9 (CH_2), 43.4 (CH_2), 42.9 (CH_2), 35.6 (CH_2), 26.0 (CH_2), 25.5 (CH_2). HRMS (ESI$^+$) $C_{31}H_{36}N_3O_4$ (MH^+) theoretical 514.2700; measured 514.2709 (−1.7 ppm error); $C_{31}H_{35}N_3NaO_4$ (MNa^+) theoretical 536.2520; measured 236.2529 (−1.7 ppm error); $C_{31}H_{35}KN_3O_4$ (MK^+) theoretical 552.2259; measured 552.2254 (+0.9 ppm error).

9-Benzyl-2-phenyl-1,5,9-triazacyclotridecane-4,13-dione (**19a**)

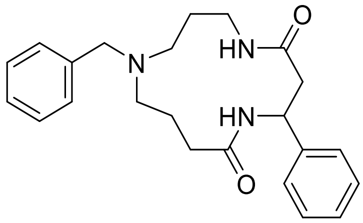

A solution of imide **18a** (884 mg, 1.72 mmol) and palladium hydroxide (103 mg, 0.19 mmol, 20 wt% on carbon) in ethanol (17.2 mL) was prepared and purged under vacuum and charged with argon. The flask was purged under vacuum and charged with hydrogen gas and stirred at room temperature for 16 hours. The reaction was acidified with conc. sulfuric acid until the white precipitate redissolved. The solution was filtered through Celite™, washed with methanol. The filtrate was concentrated under vacuum, the residue was diluted with dichloromethane (20 mL) and basified with DIPEA to precipitate the product as a white amorphous solid. The solid was collected via vacuum filtration and washed sequentially with ethyl acetate and diethyl ether to afford the title compound as a white amorphous solid (480 mg, 1.3 mmol, 74%). Mp (decomposition) 225 °C. R_f 0.55 (13:6:1 ethyl acetate:methanol:triethylamine). ν_{max} (thin film)/cm^{-1} 3304, 3062, 2944, 1634, 1539, 1438, 1353, 1285, 1212, 1130, 1070, 1028, 963, 731, 697, 640, 540, 526, 461. ^{1}H NMR (400 MHz, d_6-DMSO) δ 8.07 (1H, d, J = 8.5 Hz, NH), 7.66 (1H, dd, J = 6.5, 4.5 Hz, NH), 7.35–7.14 (10H, m, PhH), 5.20–5.07 (1H, m, CH), 3.64–3.42 (2H, m, $PhCH_2$), 2.74–2.63 (1H, m, CH_2), 2.60–2.53 (2H, m, CH_2), 2.46–2.23 (6H, m, CH_2), 1.93–1.82 (1H, m, CH_2), 1.77–1.54 (3H, m, CH_2), 1.51–1.36 (1H, m, CH_2). ^{13}C NMR (101 MHz, d_6-DMSO) δ 171.8 (CO), 170.1 (CO), 144.0 (PhC), 140.4 (PhC), 129.1 (PhCH), 128.9 (PhCH), 128.6 (PhCH), 127.3 (PhCH), 127.2 (PhCH), 126.5 (PhCH), 58.5 (NCH_2), 51.2 (NCH_2), 51.0 (NCH_2), 50.9 (CH), 44.0 (NCH_2), 37.5

(CH_2), 34.0 (CH_2), 26.0 (CH_2), 23.4 (CH_2). HRMS (ESI$^+$) $C_{23}H_{30}N_3O_2$ (MH^+) theoretical 380.2333; measured 380.2335 (−0.5 ppm error); $C_{23}H_{29}N_3NaO_2$ (MNa^+) theoretical 402.2152; measured 402.2149 (+0.9 ppm error).

(9H-Fluoren-9-yl)methyl (3-(5-(4-methoxyphenyl)-9-oxo-1,5-diazonan-1-yl)-3-oxo-1-phenylpropyl)carbamate (18b) – racemic and *R*-enantiomer

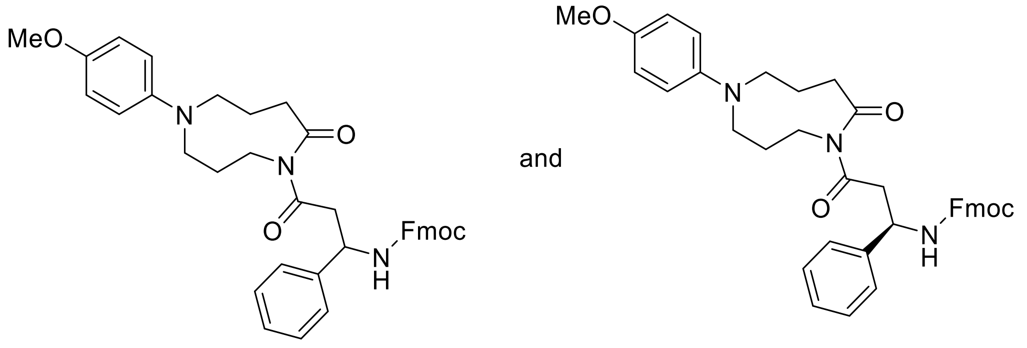

To a stirring solution of lactam **13c** (301 mg, 1.2 mmol), DMAP (14.7 mg, 0.12 mmol) and pyridine (0.58 mL, 7.2 mmol) in dry dichloromethane (12.0 mL) was added a solution of acid chloride **18a** (1.46 mmol, 1.5 eq. freshly prepared from carboxylic acid **S2** using general procedure C) in dichloromethane (1 mL) and stirred at 50 °C for 16 hours. The reaction was diluted with water (20 mL), the aqueous phase was extracted with dichloromethane (3 × 20 mL). The combined organic layers dried with sodium sulfate, filtered, and concentrated under vacuum. The crude was purified via flash column chromatography (1:1 n-hexane:ethyl acetate) to afford the title compound as a yellow oil (631 mg, 1.02 mmol, 85%). R_f 0.30 (1:1 n-hexane:ethyl acetate). ν_{max} (thin film)/cm^{-1} 3375, 2935, 1693, 1513, 1451, 1376, 1329, 1244, 1169, 1086, 1038, 910, 817, 738. Note: both the ^{1}H and ^{13}C NMR spectra obtained for this product showed severe broadening of their NMR signals due to rotamers. ^{1}H NMR (400 MHz, $CDCl_3$) δ 7.88–7.68 (2H, m, ArH), 7.68–7.51 (2H, m, ArH), 7.49–7.09 (10H, m, ArH), 6.87–6.65 (2H, m, ArH), 6.65–6.45 (2H, m, ArH), 5.67 (1H, d, *J* = 8.5 Hz, NH), 5.21–5.11 (1H, m, CH), 4.56–4.31 (2H, m, OCH_2), 4.31–4.10 (1H, m, CH_2), 4.07–3.91 (1H, m, CH_2), 3.90–3.74 (1H, m, CH_2), 3.72–3.54 (3H, m, CH_3), 3.41–3.14 (4H, m, NCH_2), 3.10–2.91 (2H, m, $COCH_2$), 2.83–2.68 (1H, m, CH_2), 2.68–2.53 (1H, m, $COCH_2$), 2.31–2.09 (2H, m, CH_2), 1.98–1.63 (2H, m, CH_2). ^{13}C NMR (101 MHz, $CDCl_3$) δ 177.1 (CO), 173.8 (CO), 155.8 (CO), 152.3 (ArC), 144.2 (ArC), 142.6 (ArC), 141.9 (ArC), 141.4 (ArC), 128.7 (ArCH), 127.8 (ArCH), 127.4 (PhCH), 127.2 (ArCH), 126.3 (ArCH), 125.2 (ArCH), 120.1 (ArCH), 114.8 (ArCH), 114.7 (ArCH), 66.4 (OCH_2), 56.5 (NCH_2), 55.6 (CH_3), 54.0 (NCH_2), 52.1 (NCH), 47.4 (CH_2CH), 44.8 (CH_2), 44.0 (CH_2), 33.3 (CH_2), 27.9 (CH_2), 26.0 ($COCH_2$). HRMS (ESI$^+$) $C_{38}H_{39}N_3NaO_5$ (MNa^+) theoretical 640.2782; measured 640.2818 (−5.6 ppm error).

(*R*)-18b - Synthesised as above, starting from **(*R*)-18b**. All spectroscopic data were the same as above.

9-(4-Methoxyphenyl)-2-phenyl-1,5,9-triazacyclotridecane-4,13-dione (19b) – racemic and *R*-enantiomer

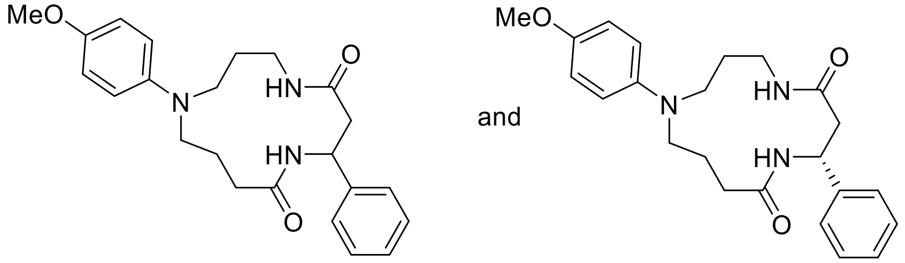

Racemic 19b

To a solution of imide **18b** (631 mg, 1.02 mmol) in dichloromethane (12 mL) was added DBU (1.80 mL, 12.1 mmol) and stirred at room temperature for 16 hours. The reaction was diluted with dichloromethane (20 mL) and water (20 mL) to precipitate product as a white solid. The solid was collected via vacuum filtration, the solid was washed with dichloromethane to afford the title compound as an off white amorphous solid (399 mg, 1.01 mmol, 99%). Mp 314 °C (decomposition). R_f 0.32 (1:9 methanol:ethyl acetate). ν_{max} (thin film)/cm^{-1} 3290, 2933, 1639, 1547, 1512, 1432, 1353, 1238, 1045, 815, 697, 529. ^{1}H NMR (400 MHz, d_6-DMSO) δ 8.35 (1H, d, *J* = 8.5 Hz, NH), 7.79 (1H, t, *J* = 6.0 Hz, NH), 7.34–7.15 (5H, m, PhH), 6.71 (2H, d, *J* = 9.0 Hz, ArH), 6.56 (2H, d, *J* = 9.0 Hz, ArH), 5.32–5.21 (1H, m, CH), 3.60 (3H, s, CH_3), 3.34–3.05 (4H, m, NCH_2), 2.95–2.83 (2H, m, NCH_2), 2.59–2.48 (1H, m, $COCH_2$), 2.33–2.22 (1H, m, $COCH_2$), 2.08–1.97 (1H, m, $COCH_2$), 1.87–1.60 (3H, m, CH_2), 1.58–1.44 (1H, m, CH_2). ^{13}C NMR (101 MHz, d_6-DMSO) δ 171.7 (CO), 170.3 (CO), 150.5 (ArCO), 143.9 (ArC), 143.8 (ArC), 128.9 (PhCH), 127.3 (PhCH), 126.6 (PhCH), 115.4 (ArCH), 112.7 (ArCH), 55.9 (CH_3), 50.9 (CH), 47.7 (NCH_2), 46.5 (NCH_2), 43.9 ($COCH_2$), 36.6 ($NHCH_2$), 33.8 ($COCH_2$), 25.9 (CH_2), 24.1 (CH_2). HRMS (ESI$^+$) $C_{23}H_{30}N_3O_3$ (MH^+) theoretical 396.2282; measured 396.2267 (+3.8 ppm error); $C_{23}H_{29}N_3NaO_3$ (MNa^+) theoretical 418.2101; measured 418.2106 (−1.1 ppm error); $C_{23}H_{29}KN_3O_3$ (MK^+) theoretical 434.1840; measured 434.1847 (−1.4 ppm error).

(*R*)-19b

Synthesised as above, starting from **(*R*)-18b**. All spectroscopic data were the same as above, with the exception of: $[\alpha]_D^{20}$ +57.01 (c = 1.0).

Chiral HPLC for rac-19b and (*R*)-19b

Chiral HPLC results were obtained using a ChiralPak-ID column with a solvent flow rate of 1 mL/min (20:80 *n*-hexane:IPA) over 60 minutes to confirm no epimerisation took place during the synthesis of **(*R*)-19b**.

rac-19b

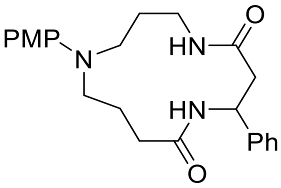

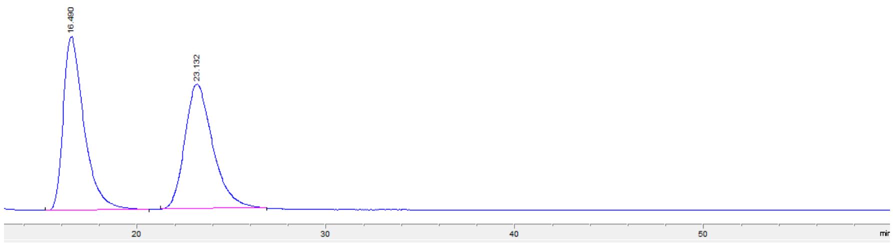

#	Time	Area	Height	Width	Area%	Symmetry
1	16.49	1171.2	15.3	1.0498	50.826	0.587
2	23.132	1133.2	11	1.2178	49.174	0.716

(*R*)-19b

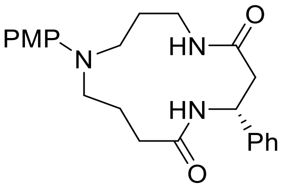

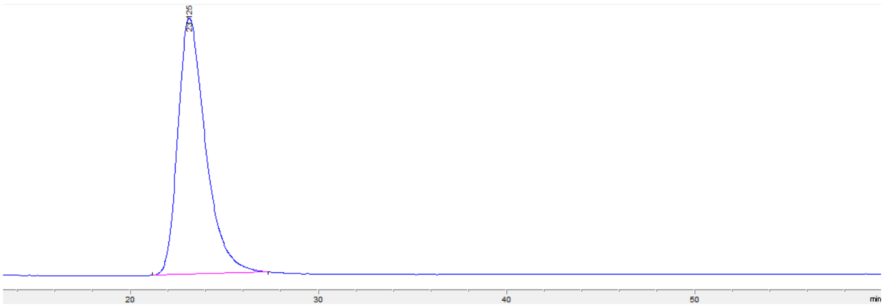

#	Time	Area	Height	Width	Area%	Symmetry
1	23.125	3119.4	30.5	1.3909	100.000	0.655

9-(4-Methoxyphenyl)-2-methyl-1,5,9-triazacyclotridecane-4,13-dione (**19c**)

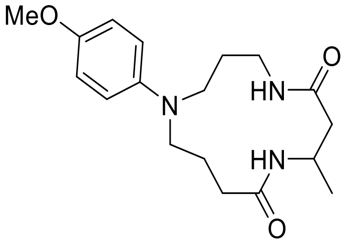

To a stirring solution of lactam **13c** (100 mg, 0.40 mmol), DMAP (4.8 mg, 0.039 mmol) and pyridine (0.20 mL, 2.48 mmol) in dry dichloromethane (4 mL) was added a solution of acid chloride **17c** (0.60 mmol, 1.5 eq. freshly prepared from carboxylic acid **S3** using general procedure C) in dichloromethane (0.5 mL) and stirred at 50 °C for 16 hours. The reaction was diluted with water (20 mL), the aqueous phase was extracted with dichloromethane (3 × 20 mL). The combined organic layers dried with sodium sulfate, filtered, and concentrated under vacuum. The crude was redissolved in dry dichloromethane (4 mL), followed by the addition of DBU (0.60 mL, 4.0 mmol) and stirred at room temperature for 16 hours. The reaction was diluted with dichloromethane (20 mL) and water (20 mL), the aqueous phase was extracted with dichloromethane (3 × 20 mL). The combined organic layers dried with sodium sulfate, filtered, concentrated under vacuum and purified via flash column chromatography (ethyl acetate) to afford the title compound as an off white amorphous solid (50.7 mg, 0.152 mmol, 38%). Mp 257 °C. R_f 0.45 (1:9 methanol:ethyl acetate). ν_{max} (thin film)/cm^{-1} 3282, 2927, 1641, 1551, 1516, 1244, 1210, 1163, 1038, 803. ^{1}H NMR (400 MHz, $CDCl_3$) δ 6.80 (2H, d, J = 9.0 Hz, ArH), 6.68 (2H, d, J = 9.0 Hz, ArH), 4.16 (1H, qtd, J = 7.0, 7.0, 5.5 Hz, CH), 3.74 (3H, s, OCH_3), 3.52–3.29 (2H, m, CH_2), 3.29–3.05 (4H, m, 2×CH_2), 2.76–2.49 (2H, m, $COCH_2$), 2.34–2.21 (2H, m, CH_2), 2.20–2.11 (1H, m, CH_2), 2.00–1.56 (4H, m, 2×CH_2), 1.37 (3H, d, J = 7.0 Hz, CH_3). ^{13}C NMR (101 MHz, $CDCl_3$) δ 172.9 (CO), 171.2 (CO), 152.1 (ArCO), 143.5 (ArC), 115.1 (ArCH), 114.0 (ArCH), 55.9 (OCH_3), 50.0 (NCH_2), 48.8 (NCH_2), 44.3 (CH), 42.3 (NCH_2), 37.4 ($COCH_2$), 34.3 ($COCH_2$), 26.5 (CH_2), 23.3 (CH_2), 20.9 (CH_3). HRMS (ESI$^+$) $C_{18}H_{28}N_3O_3$ (MH^+) theoretical 334.2125; measured 334.2148 (−6.7 ppm error).

9-(4-Methoxyphenyl)-1-methyl-1,5,9-triazacyclotridecane-4,13-dione (**19d**)

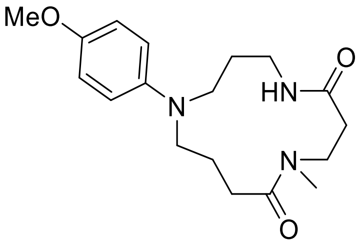

To a stirring solution of lactam **13c** (100 mg, 0.40 mmol), DMAP (4.9 mg, 0.04 mmol) and pyridine (0.20 mL, 2.48 mmol) in dry dichloromethane (4 mL) was added a solution of acid chloride **17d** (0.60 mmol, 1.5 eq. freshly prepared from carboxylic acid **S4** using general procedure C) in dichloromethane (0.5

mL) and stirred at 50 °C for 16 hours. The reaction was diluted with water (20 mL), the aqueous phase was extracted with dichloromethane (3 × 20 mL). The combined organic layers dried with sodium sulfate, filtered, and concentrated under vacuum. The crude was redissolved in dry dichloromethane (4 mL), followed by the addition of DBU (0.60 mL, 4.0 mmol) and stirred at room temperature for 16 hours. The reaction was diluted with dichloromethane (20 mL) and water (20 mL), the aqueous phase was extracted with dichloromethane (3 × 20 mL). The combined organic layers dried with sodium sulfate, filtered, concentrated under vacuum and purified via flash column chromatography (ethyl acetate →19:1 ethyl acetate:methanol) to afford the title compound as an off white amorphous solid (76.3 mg, 0.23 mmol, 57%). Mp 116 °C. R_f 0.29 (9:1 ethyl acetate:methanol). ν_{max} (thin film)/cm^{-1} 3297, 2932, 1628, 1511, 1444, 1362, 1242, 1205, 1037, 813, 730, 700, 574, 519. Note: The ^{1}H NMR spectra obtained for this product showed severe broadening of their NMR signals due to rotamers. ^{1}H NMR (400 MHz, $CDCl_3$) δ 6.79 (2H, d, *J* = 9.0 Hz, ArH), 6.68 (2H, d, *J* = 9.0 Hz, ArH), 3.72 (3H, s, OCH_3), 3.69–3.22 (3H, m, CH_2), 3.14–3.05 (1H, m, CH_2), 3.04 (3H, s, NCH_3), 2.99–2.57 (4H, m, CH_2), 2.57–2.21 (3H, m, CH_2), 1.99–1.83 (2H, m, CH_2), 1.83–1.35 (3H, m, CH_2). ^{13}C NMR (101 MHz, $CDCl_3$) δ 173.6 (CO), 171.0 (CO), 152.0 (ArCO), 143.7 (ArC), 115.1 (ArCH), 115.0 (ArCH), 55.9 (OCH_3), 49.3 (NCH_2), 48.6 (NCH_2), 45.6 (NCH_2), 38.0 (NCH_3), 37.3 (NCH_2), 35.1 ($COCH_2$), 30.4 ($COCH_2$), 26.5 (CH_2), 23.3 (CH_2). HRMS (ESI$^+$) $C_{18}H_{28}N_3O_3$ (MH^+) theoretical 334.2125; measured 334.2124 (+0.2 ppm error); $C_{18}H_{27}N_3NaO_3$ (MNa^+) theoretical 356.1945; measured 356.1957 (−3.6 ppm error).

9-(4-Methoxyphenyl)-1,5,9-triazacyclotridecane-4,13-dione (**19e**)

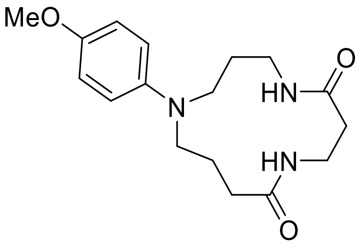

To a stirring solution of lactam **13c** (100.6 mg, 0.41 mmol), DMAP (5.2 mg, 0.043 mmol) and pyridine (0.20 mL, 2.48 mmol) in dry dichloromethane (4 mL) was added a solution of acid chloride **17e** (0.60 mmol, 1.5 eq. freshly prepared from Fmoc-β-alanine using general procedure C) in dichloromethane (0.5 mL) and stirred at 50 °C for 16 hours. The reaction was diluted with water (20 mL), the aqueous phase was extracted with dichloromethane (3 × 20 mL). The combined organic layers dried with sodium sulfate, filtered, and concentrated under vacuum. The crude was redissolved in dry dichloromethane (4 mL), followed by the addition of DBU (0.60 mL, 4.0 mmol) and stirred at room temperature for 16 hours. The reaction was diluted with dichloromethane (20 mL) and water (20 mL), the aqueous phase was extracted with dichloromethane (3 × 20 mL). The combined organic layers dried with sodium sulfate, filtered, concentrated under vacuum and purified via flash column

chromatography (ethyl acetate) to afford the title compound as an off white amorphous solid (72.1 mg, 0.23 mmol, 56%). Mp 236 °C. R_f 0.42 (1:9 methanol:ethyl acetate). ν_{max} (thin film)/cm^{-1} 3291, 2929, 1639, 1550, 1516, 1440, 1383, 1256, 1200, 1041, 810, 731. ^{1}H NMR (400 MHz, $CDCl_3$) δ 6.80 (2H, d, *J* = 9.0 Hz, ArH), 6.64 (2H, d, *J* = 9.0 Hz, ArH), 3.73 (3H, s, OCH_3), 3.53 (2H, td, *J* = 6.0, 6.0 Hz, $NHCH_2$), 3.32 (2H, td, *J* = 6.0, 6.0 Hz, $NHCH_2$), 3.18 (2H, t, *J* = 7.0 Hz, NCH_2), 3.18 (2H, t, *J* = 6.5 Hz, NCH_2), 2.61 (2H, t, *J* = 6.0 Hz, $COCH_2$), 2.28–2.20 (2H, m, $COCH_2$), 1.94–1.81 (2H, m, CH_2), 1.76–1.66 (2H, m, CH_2). ^{13}C NMR (101 MHz, $CDCl_3$) δ 173.5 (CO), 171.6 (CO), 151.6 (ArCO), 143.1 (ArC), 115.2 (ArCH), 113.8 (ArCH), 56.0 (OCH_3), 49.6 (NCH_2), 48.0 (NCH_2), 37.0 (NCH_2), 36.6 (NCH_3), 35.9 ($COCH_2$), 33.9 ($COCH_2$), 27.2 (CH_2), 23.6 (CH_2). HRMS (ESI$^+$) $C_{17}H_{26}N_3O_3$ (MH^+) theoretical 320.1969; measured 320.1972 (−1.0 ppm error); $C_{17}H_{25}N_3NaO_3$ (MNa^+) theoretical 342.1788; measured 342.1795 (−2.0 ppm error).

2-Phenyl-1,5,9-triazacyclotridecane-4,13-dione (21) – racemic and *R*-enantiomer

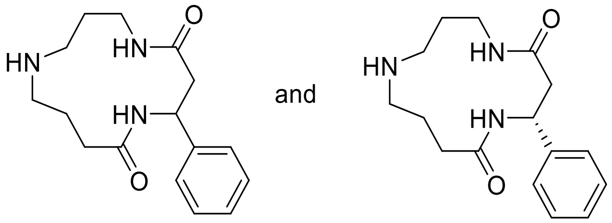

Method 1

A suspension of lactam **19a** (100 mg, 0.26 mmol) and palladium hydroxide (14.8 mg, 0.028 mmol, 20 wt% on carbon) in methanol (2.6 mL) was prepared and acidified with acetic acid dropwise until the white suspension dissolves. The solution was purged under vacuum and charged with argon. The flask was purged under vacuum and charged with hydrogen gas and stirred at room temperature for 16 hours. The reaction was filtered through Celite™, washed with methanol. The filtrate was concentrated under vacuum and purified via flash column chromatography (1:6:13 NEt_3:methanol:ethyl acetate) to afford the title compound as a white amorphous solid (67.3 mg, 0.23 mmol, 88%).

Method 2

A solution of lactam **19a** (499 mg, 1.31 mmol) and palladium hydroxide (70.0 mg, 0.132 mmol, 20 wt% on carbon) in methanol (13 mL) was prepared and acidified with sulfuric acid until white suspension dissolves. The solution was purged under vacuum and charged with argon. The flask was purged under vacuum and charged with hydrogen gas and stirred at room temperature for 16 hours. The reaction was filtered through Celite™, washed with methanol. The filtrate was concentrated under vacuum and purified via flash column chromatography (1:6:13 NEt_3:methanol:ethyl acetate) to afford the title compound as a white amorphous solid (287.4 mg, 0.99 mmol, 76%).

Method 3

To a stirring suspension of lactam **19b** (26.0 mg, 0.0657 mmol) in water (0.75 mL) and acetonitrile (0.75 mL), periodic acid (15.7 mg, 0.0690 mmol) was added and stirred at room temperature for 24 hours. The reaction mixture was acidified to pH 1 with 0.2 M aqueous hydrochloric acid and washed with dichloromethane (2 × 10 mL). The aqueous layer was then adjusted to pH 11 with 1 M aqueous NaOH and extracted with a 3:1 mixture of chloroform:isopropanol (3 × 10 mL). The combined organic layers were dried with magnesium sulfate, filtered and concentrated under vacuum to afford the title compound as an off white amorphous solid (19.0 mg, 0.0657 mmol, 100%).

Racemic 21

Mp 146 °C. R_f 0.10 (1:6:13 NEt_3:methanol:ethyl acetate). ν_{max} (thin film)/cm^{-1} 3300, 2942, 2437, 1718, 1637, 1547, 1368, 1284, 1026, 758, 705, 666, 616, 543, 500. ^1H NMR (400 MHz, d_4-MeOD) δ 7.37–7.21 (5H, m, PhH), 5.31 (1H, dd, *J* = 12.2, 2.9 Hz, CH), 3.52 (1H, dt, *J* = 13.8, 4.7 Hz, NCH_2), 3.15 (1H, dt, *J* = 13.5, 6.7 Hz, NCH_2), 2.93–2.85 (1H, m, NCH_2), 2.83–2.54 (4H, m, $COCH_2$ and NCH_2), 2.40–2.24 (2H, m, $COCH_2$), 2.07–1.96 (1H, m, CH_2) , 1.89–1.70 (3H, m, CH_2). ^{13}C NMR (101 MHz, d_4-MeOD) δ 175.7 (C), 173.2 (C), 143.5 (C), 129.7 (2 × CH), 128.5 (CH), 127.2 (2 × CH), 51.7 (CH), 50.5 (CH_2), 50.2 (CH_2), 44.1 (CH_2), 41.4 (CH_2), 35.9 (CH_2), 28.2 (CH_2), 25.4 (CH_2). HRMS (ESI^+) $C_{16}H_{24}N_3O_2$ (MH^+) theoretical 290.1863; measured 290.1870 (−2.5 ppm error); $C_{16}H_{23}N_3NaO_2$ (MNa^+) theoretical 312.1682; measured 312.1687 (−1.3 ppm error); $C_{16}H_{23}KN_3O_2$ (MK^+) theoretical 328.1422; measured 328.1424 (−0.5 ppm error).

(*R*)-21

Synthesised as above using Method 3 (56.6 mg, 0.196 mmol, 70%), starting from **(*R*)-19b**. All spectroscopic data were the same as above, with the exception of: $[\alpha]_D^{20}$ +5.83 (c = 1.0).

9-(Furan-3-carbonyl)-2-phenyl-1,5,9-triazacyclotridecane-4,13-dione (2) – racemic and *R*-enantiomer

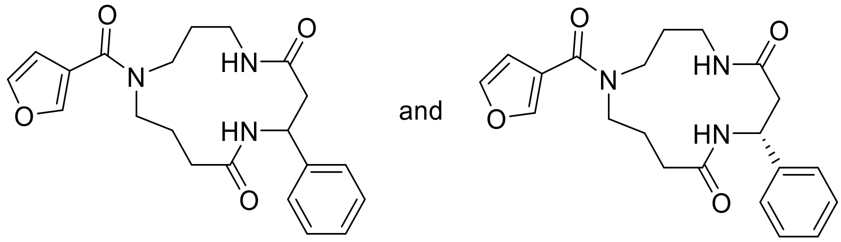

rac-2

To a stirring suspension of lactam **21** (19.0 mg, 0.0657 mmol), 3-furoic acid (8.8 mg, 0.0789 mmol) and diisopropyl ethylamine (0.0687 mL, 0.394 mmol) in dry dichloromethane (2.0 mL) was added T3P (0.0587 mL, 0.0986 mmol, 50 wt% in ethyl acetate) and stirred at room temperature for 24 hours. Saturated aqueous sodium hydrogen carbonate (10 mL) was then added to the reaction mixture and extracted with a 3:1 mixture of chloroform:isopropanol (3 × 10 mL). The combined organic layers were dried with magnesium sulfate, filtered, concentrated under vacuum and purified via flash column chromatography (10:1 ethyl acetate:methanol) to afford the title compound as a white amorphous solid (19.4 mg, 0.0506 mmol, 77%). M.p. (decomposition) 260 °C. R_f 0.15 (9:1 ethyl acetate:methanol). ν_{max} (thin film)/cm^{-1} 3286, 1636, 1547, 1503, 1428, 1356, 1283, 1156, 875, 734, 699, 602, 528. Note: both the ^{1}H and ^{13}C NMR spectra obtained for this product showed severe broadening of their NMR signals due to rotamers (1:1 ratio). ^{1}H NMR (700 MHz, d_6-DMSO) δ 8.54–8.39 (1H, m, NH), 8.02 (1H, d, *J* = 10.1 Hz, ArH), 7.94 (1H, t, *J* = 5.7 Hz, NH), 7.73 (1H, d, *J* = 11.1 Hz, ArH), 7.35–7.31 (4H, m, PhH), 7.25–7.21 (1H, m, PhH), 6.66 (1H, d, *J* = 6.8 Hz, ArH), 5.30 (1H, t, *J* = 9.7 Hz, CH), 3.68–3.53 (1H, m, CH_2), 3.50–3.42 (1H, m, CH_2), 3.30–3.20 (2H, m, CH_2), 3.18–3.08 (1H, m, CH_2), 3.01–2.83 (2H, m, CH_2), 2.80–2.69 (1H, m, CH_2), 2.63–2.53 (1H, m, CH_2), 2.49–2.44 (1H, m, CH_2), 2.38–2.25 (1H, m, CH_2), 2.08–1.68 (4H, m, CH_2), 1.67–1.57 (1H, m, CH_2). ^{13}C NMR (176 MHz, d_6-DMSO) δ 170.8 (CO, rotamer 1), 170.7 (CO, rotamer 2), 169.9 (CO, rotamer 1), 169.9 (CO, rotamer 2), 163.5 (CO, rotamer 1), 163.4 (CO, rotamer 2), 143.4 (ArCH/PhC, both rotamers/rotamer 1), 143.3 (PhC, rotamer 2), 143.0 (ArCH, rotamer 1), 142.9 (ArCH, rotamer 2), 128.4 (2 × PhCH, both rotamers), 126.8 (PhCH, both rotamers), 126.1 (2 × PhCH, both rotamers), 121.2 (ArC, both rotamers), 110.5 (ArCH, both rotamers), 50.4 (CH, both rotamers), 45.6 (CH_2, rotamer 1), 45.3 (CH_2, rotamer 2), 43.2 (CH_2, both rotamers), 42.4 (CH_2, rotamer 1), 41.8 (CH_2, rotamer 2), 35.9 (CH_2, both rotamers), 33.4 (CH_2, rotamer 1), 33.1 (CH_2, rotamer 2), 27.1 (CH_2, rotamer 1), 25.7 (CH_2, rotamer 2), 25.1 (CH_2, rotamer 1), 23.3 (CH_2, rotamer 2). HRMS (ESI$^+$) $C_{21}H_{26}N_3O_4$ (MH^+) theoretical 384.1918; measured 384.1917 (+0.2 ppm error); $C_{21}H_{25}N_3NaO_4$ (MNa^+) theoretical 406.1737; measured 406.1742 (−1.1 ppm error); $C_{21}H_{25}KN_3O_4$ (MK^+) theoretical 422.1477; measured 422.1488 (−2.8 ppm error).

2

Synthesised as above (44.6 mg, 0.116 mmol, 66%), starting from **(*R*)-21**. All spectroscopic data were the same as above, with the exception of: $[\alpha]_D^{20}$ +42.42 (c = 1.0).

9-Benzoyl-2-phenyl-1,5,9-triazacyclotridecane-4,13-dione (**22a**)

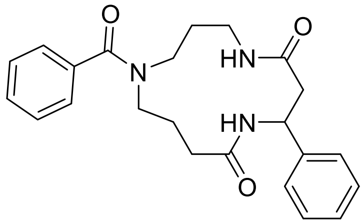

To a stirring suspension of lactam **21** (50.1 mg, 0.173 mmol), benzoic acid (27.1 mg, 0.222 mmol) and diisopropyl ethylamine (0.18 mL, 1.03 mmol) in dry dichloromethane (1.73 mL) was added T3P (0.15 mL, 0.252 mmol, 50 wt% in ethyl acetate) and stirred at room temperature for 24 hours. Water (5 mL) was added to the reaction and stirred for 1 hour to form a solid suspension. The reaction mixture was filtered and the solid was washed with diethyl ether to afford the title compound as a white amorphous solid (40.5 mg, 0.106 mmol, 61%). Mp (decomposition) 253 °C. R_f 0.18 (9:1 ethyl acetate:methanol). ν_{max} (thin film)/cm^{-1} 3288, 1636, 1544, 1496, 1467, 1424, 1357, 1283, 1227, 1211, 1138, 1028, 701, 535. Note: both the ^{1}H and ^{13}C NMR spectra obtained for this product showed severe broadening of their NMR signals due to rotamers (1:1 ratio). ^{1}H NMR (400 MHz, d_6-DMSO) δ 8.45 (1H, d, *J* = 9.0 Hz, NH, rotamer 1), 8.37 (1H, d, *J* = 9.0 Hz, NH, rotamer 2), 7.92 (1H, s (br), NH, rotamer 1) , 7.84 (1H, s (br), NH, rotamer 2), 7.53–7.04 (10H, m, PhH; both rotamers), 5.25 (1H, s (br), CH, both rotamers), 3.68–3.50 (1H, m, NCH_2, both rotamers), 3.18–3.02 (2H, m, NCH_2, both rotamers), 3.02–2.88 (1H, m, CH_2, both rotamers), 2.88–2.60 (3H, m, CH_2, both rotamers), 2.37–2.14 (1H, m, CH_2, both rotamers), 2.14–1.99 (1H, m, CH_2, both rotamers), 1.99–1.52 (4H, m, CH_2, both rotamers), 1.52–1.32 (1H, m, CH_2, both rotamers). ^{13}C NMR (176 MHz, d_6-DMSO) δ 171.2 (CO, rotamer 1), 171.0 (CO, rotamer 2), 170.9 (CO, rotamer 1), 170.8 (CO, rotamer 2), 170.3 (CO, both rotamers), 143.7 (PhC, rotamer 1), 143.6 (PhC, rotamer 2), 137.6 (PhC, rotamer 1), 137.5 (PhC, rotamer 2), 129.5 (PhCH, both rotamers), 128.9 (PhCH, both rotamers), 128.8 (PhCH, both rotamers), 127.3 (PhCH, both rotamers), 126.6 (PhCH, both rotamers), 126.5 (PhCH, both rotamers), 50.7 (CH, both rotamers), 46.7 (NCH_2, rotamer 1), 46.3 (NCH_2, rotamer 2), 43.6 (CH_2, both rotamers), 42.0 (CH_2, rotamer 1), 41.2 (CH_2, rotamer 2), 36.2 (NCH_2, both rotamers), 33.9 (NCH_2, rotamer 1), 33.3 (CH_2, rotamer 2), 27.4 (CH_2, rotamer 1), 26.3 (CH_2, rotamer 2), 25.2 (CH_2, rotamer 1), 23.7 (CH_2, rotamer2). HRMS (ESI$^+$) $C_{23}H_{27}N_3NaO_3$ (MNa^+) theoretical 416.1945; measured 416.1944 (+0.1 ppm error); $C_{23}H_{27}KN_3O_3$ (MK^+) theoretical 432.1684; measured 432.1694 (−2.3 ppm error).

9-Cinnamoyl-2-phenyl-1,5,9-triazacyclotridecane-4,13-dione (22b)

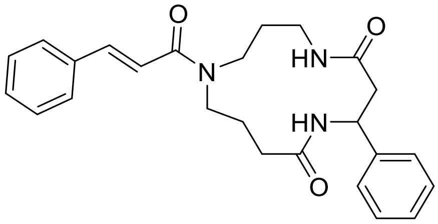

To a stirring suspension of lactam **21** (49.8 mg, 0.172 mmol), cinnamic acid (31.9 mg, 0.215 mmol) and diisopropyl ethylamine (0.18 mL, 1.03 mmol) in dry dichloromethane (1.73 mL) was added T3P (0.15 mL, 0.252 mmol, 50 wt% in ethyl acetate) and stirred at room temperature for 24 hours. Water (5 mL) was added to the reaction and stirred for 1 hour to form a solid suspension. The reaction mixture was filtered and the solid was washed with diethyl ether to afford the title compound as a white amorphous solid (54.2 mg, 0.141 mmol, 82%). Mp (decomposition) 240 °C. R_f 0.18 (9:1 ethyl acetate:methanol). ν_{max} (thin film)/cm^{-1} 3299, 1643, 1590, 1543, 1496, 1429, 1357, 1256, 1197, 1114, 980, 767, 712, 686, 617, 522. Note: both the ^{1}H and ^{13}C NMR spectra obtained for this product showed severe broadening of their NMR signals due to rotamers (1:1 ratio). Several ^{1}H NMR signals were overlapped with H_2O peak. ^{1}H NMR (700 MHz, d_6-DMSO) δ 8.51–8.45 (1H, m, NH, both rotamers), 7.99–7.88 (1H, m, NH, both rotamers), 7.76–7.69 (2H, t, *J* = 7.5 Hz, PhH), 7.50 (1H, d, *J* = 15.5 Hz, CH, both rotamers), 7.44–7.37 (3H, m, PhH, both rotamers), 7.37–7.31 (4H, m, PhH, both rotamers), 7.27–7.22 (1H, m, PhH, both rotamers), 7.16 (1H, d, *J* = 15.5 Hz, rotamer 1), 7.12 (1H, d, *J* = 15.5 Hz, rotamer 2), 5.41–5.52 (1H, m, CH, both rotamers), 3.79–3.56 (2H, m, NCH_2, both rotamers), 3.32–3.22 (1H, m, CH_2, both rotamers), 3.14–3.05 (1H, m, CH_2, rotamer 1), 3.05–2.98 (1H, m, CH_2, both rotamers), 2.98–2.89 (1H, m, CH_2, rotamer 2), 2.78–2.68 (1H, m, CH_2, rotamer 1), 2.64–2.55 (1H, m, CH_2, both rotamers), 2.39–2.26 (1H, m, CH_2, both rotamers), 2.19–2.09 (1H, m, CH_2, rotamer 1), 2.07–1.99 (1H, m, CH_2, rotamer 2), 1.97–1.84 (2H, m, CH_2, both rotamers), 1.84–1.76 (1H, m, CH_2, both rotamers), 1.75–1.60 (1H, m, CH_2, both rotamers), 1.60–1.44 (1H, m, CH_2, both rotamers). ^{13}C NMR (176 MHz, d_6-DMSO) δ 171.4 (CO, rotamer 1), 171.2 (CO, rotamer 2), 170.3 (CO, rotamer 1), 170.3 (CO, rotamer 2), 165.6 (CO, rotamer 1), 165.5 (CO, rotamer 2), 143.7 (CH, rotamer 1), 143.7 (CH, rotamer 2), 141.9 (PhC, both rotamers), 135.6 (PhC, both rotamers), 130.0 (PhCH, both rotamers), 129.2 (PhCH, both rotamers), 128.8 (PhCH, both rotamers), 128.5 (PhCH, both rotamers), 127.3 (PhCH, both rotamers), 126.6 (PhCH, both rotamers), 118.9 (CH, rotamer 1), 118.8 (CH, rotamer 2), 50.8 (CH, both rotamers), 44.5 (CH_2, rotamer 1), 43.9 (CH_2, rotamer 2), 43.7 (CH_2, rotamer 1), 43.3 (CH_2, rotamer 2), 42.4 (CH_2, rotamer 1), 40.4 (CH_2, rotamer 2), 36.5 (CH_2, rotamer 1) 36.3 (CH_2, rotamer 2), 33.9 (CH_2, rotamer 1), 33.6 (CH_2, rotamer 2), 28.2 (CH_2, rotamer 1), 26.4 (CH_2, rotamer 2), 26.2 (CH_2, rotamer 1), 24.3 (CH_2, rotamer 2). HRMS (ESI$^+$) $C_{25}H_{29}N_3NaO_3$ (MNa^+) theoretical 442.2101; measured 442.2108 (−1.5 ppm error); $C_{25}H_{29}KN_3O_3$ (MK^+) theoretical 458.1840; measured 458.1836 (+0.9 ppm error).

9-(2-(4-(Dimethylamino)phenyl)acetyl)-2-phenyl-1,5,9-triazacyclotridecane-4,13-dione (**22c**)

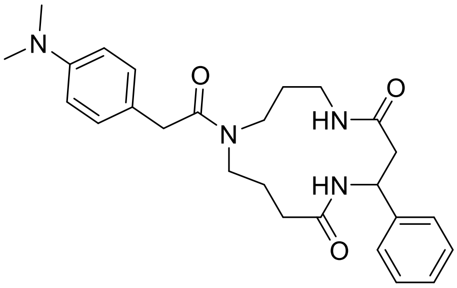

To a stirring suspension of lactam **21** (49.7 mg, 0.172 mmol), 2-(4-(dimethylamino)phenyl)acetic acid (37.5 mg, 0.209 mmol) and diisopropyl ethylamine (0.18 mL, 1.03 mmol) in dry dichloromethane (1.73 mL) was added T3P (0.15 mL, 0.252 mmol, 50 wt% in ethyl acetate) and stirred at room temperature for 24 hours. Water (5 mL) was added to the reaction and stirred for 1 hour to form a solid suspension. The reaction mixture was filtered and the solid was washed with diethyl ether to afford the title compound as a white amorphous solid (36.9 mg, 0.096 mmol, 56%). Mp 197 °C. R_f 0.27 (9:1 ethyl acetate:methanol). ν_{max} (thin film)/cm^{-1} 3288, 2939, 1637, 1523, 1430, 1355, 1281, 1208, 1162, 945, 701, 530. Note: both the ^{1}H and ^{13}C NMR spectra obtained for this product showed severe broadening of their NMR signals due to rotamers (1:1 ratio).^{1}H NMR (700 MHz, d_6-DMSO) δ 8.46–8.36 (1H, m, NH, both rotamers), 7.91–7.82 (1H, m, NH, both rotamers), 7.38–7.28 (4H, m, PhH, both rotamers), 7.26–7.19 (1H, m, PhH, both rotamers), 7.08–7.00 (2H, m, ArH, both rotamers), 6.72–6.64 (2H, m, ArH, both rotamers), 5.34–5.21 (1H, m, CH, both rotamers), 3.62–3.47 (1H, m, NCH_2, both rotamers), 3.28–3.21 (1H, m, CH_2, both rotamers), 3.17 (2H, s, $ArCH_2$, both rotamers), 3.01–2.94 (1H, m, CH_2, both rotamers), 2.91–2.80 (7H, m, CH_3/CH_2, both rotamers), 2.61–2.52 (1H, m, CH_2, both rotamers), 2.49–2.42 (1H, m, CH_2, both rotamers), 2.31–2.20 (1H, m, CH_2, both rotamers), 2.04–1.94 (1H, m, CH_2, both rotamers), 1.89–1.81 (1H, m, CH_2, rotamer 1), 1.81–1.74 (1H, m, CH_2, both rotamers), 1.75–1.68 (1H, m, CH_2, rotamer 2), 1.68–1.61 (1H, m, CH_2, rotamer 1), 1.61–1.55 (1H, m, CH_2, rotamer 2), 1.55–1.48 (1H, m, CH_2, both rotamers), 1.48–1.39 (1H, m, CH_2, both rotamers). ^{13}C NMR (176 MHz, d_6-DMSO) δ 171.2 (CO, rotamer 1), 171.2 (CO, rotamer 2), 170.9 (CO, rotamer 1), 170.8 (CO, rotamer 2), 170.2 (CO, rotamer 1), 170.2 (CO, rotamer 2), 149.6 (ArC, rotamer 1), 149.6 (ArC, rotamer 2), 143.7 (ArC, rotamer 1), 143.7 (ArC, rotamer 2), 129.6 (ArCH, rotamer 1), 129.5 (ArCH, rotamer 2), 128.8 (ArCH, both rotamers), 127.2 (ArCH, both rotamers), 126.6 (ArCH, rotamer 1), 126.5 (ArCH, rotamer 2), 123.9 (ArCH, rotamer 1), 123.8 (ArCH, rotamer 2), 113.1 (ArCH, rotamer 1), 113.0 (ArCH, rotamer 2), 50.8 (CH_2, rotamer 1), 50.7 (CH_2, rotamer 2), 49.1 (CH_2, both rotamers), 45.3 (CH_2, rotamer 1), 44.9 (CH_2, rotamer 2), 43.7 (CH_2, rotamer 1), 43.6 (CH_2, rotamer 2), 42.4 (CH_2, rotamer 1), 41.6 (CH_2, rotamer 2), 40.7 (CH_3, rotamer 1), 40.5 (CH_3, rotamer 2), 39.2 (CH_2, rotamer 1), 39.2 (CH_2, rotamer 2), 36.4 (CH_2, rotamer 1), 36.4 (CH_2, rotamer 2), 33.8 (CH_2, rotamer 1), 33.5 (CH_2, rotamer 2), 27.1 (CH_2, rotamer 1), 26.3 (CH_2, rotamer 2), 25.2 (CH_2, rotamer 1), 24.1 (CH_2, rotamer 2). HRMS (ESI$^+$) $C_{26}H_{35}N_4O_3$ (MH^+)

theoretical 451.2704; measured 451.2715 (−2.5 ppm error); $C_{26}H_{34}N_4NaO_3$ (MNa^+) theoretical 473.2523; measured 473.2527 (−0.9 ppm error); $C_{26}H_{34}KN_4O_3$ (MK^+) theoretical 489.2262; measured 489.2264 (−0.2 ppm error).

(*S*)-2-Benzyl-8-(4-methoxyphenyl)-1,4,8-triazacyclododecane-3,12-dione (19f)

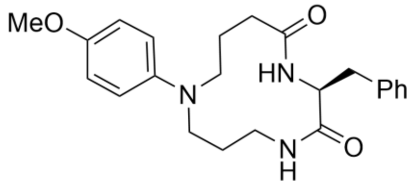

To a stirring solution of lactam **13c** (82.8 mg, 0.333 mmol), DMAP (6.1 mg, 0.05 mmol) and pyridine (0.162 mL, 2 mmol) in dry dichloromethane (3.3 mL) was added a solution of acid chloride **17f** (203 mg, 0.5 mmol, freshly prepared from N-Fmoc phenylalanine using general procedure C) in dichloromethane (0.7 mL) and stirred at 50 °C for 20 hours. The reaction was diluted with water (20 mL) and the aqueous phase was extracted with dichloromethane (3 × 20 mL). The combined organic layers were dried with magnesium sulfate, filtered, and concentrated under vacuum. The crude mixture was purified via flash column chromatography (1:1 n-hexane:ethyl acetate) to 12afford (9H-fluoren-9-yl)methyl-(*S*)-(1-(5-(4-methoxyphenyl)-9-oxo-1,5-diazonan-1-yl)-1-oxo-3-phenylpropan-2-yl)carbamate as a yellow oil (106 mg, 0.172 mmol, 52%). This compound was dissolved in dry dichloromethane (2 mL), followed by the addition of DBU (0.305 mL, 2.04 mmol) and stirred at room temperature under argon for 18 hours. The reaction mixture was concentrated under vacuum and purified via flash column chromatography (4:1 → 3:2 hexane:ethyl acetate then 19:1 dichloromethane:methanol) followed by preparative TLC (3:1 ethyl acetate:hexane) to afford the title compound as a white solid (15.7 mg, 0.0397 mmol, 12% over two steps). R_f 0.32 (ethyl acetate). Mp 208–211 °C. $[\alpha]_D^{21}$ −37.86 (c = 0.05, $CHCl_3$). ν_{max} (thin film)/cm^{-1} 3284, 2931, 1648, 1508, 1244, 1033, 752, 701. ^1H NMR (400 MHz, $CDCl_3$) δ 9.13 (1H, s, NH), 7.47–7.33 (3H, m, PhH) 7.29–7.22 (2H, m, PhH), 6.76 (2H, d, *J* = 8.8 Hz, ArH), 6.68 (2H, d, *J* = 8.8 Hz, ArH), 5.52 (1H, d, *J* = 8.5 Hz, NH), 4.73 (1H, dt, *J* = 8.5, 5.5 Hz), 3.79 (3H, s, OCH_3), 3.62–3.52 (1H, m, CH_2), 3.42 (1H, dd, *J* = 14.1, 5.5 Hz, CH_2), 3.25–3.15 (1H, m, CH_2), 3.07 (1H, dd, *J* = 14.1, 5.5 Hz, CH_2), 2.92–2.78 (1H, m, CH_2), 2.17 (1H, ddd, *J* = 14.2, 6.0, 4.0 Hz), 1.99–1.87 (1H, m, CH_2), 1.72–1.40 (4H, m CH_2). ^{13}C NMR (101 MHz, $CDCl_3$) δ 172.0 (C), 171.0 (C), 157.1 (C), 142.8 (C), 136.6 (C), 129.6 (2 × CH), 129.0 (2 × CH), 127.2 (CH), 125.5 (2 × CH), 114.7 (2 × CH), 61.0 (CH_2), 55.6 (CH_3), 53.6 (CH), 50.7 (CH_2), 41.4 (CH_2), 36.2 (CH_2), 33.2 (CH_2), 23.6 (CH_2), 21.9 (CH_2). HRMS (ESI^+) $C_{23}H_{30}N_3O_3$ (MH^+) theoretical 396.2282; measured 396.2293 (−2.7 ppm error); $C_{23}H_{29}N_3NaO_3$ (MNa^+) theoretical 418.2101; measured 418.2106 (−1.1 ppm error); $C_{23}H_{29}KN_3O_3$ (MK^+) theoretical 434.1840; measured 434.1844 (−0.7 ppm error).

(*S*)-2-Benzyl-1,4,8-triazacyclododecane-3,12-dione (23)

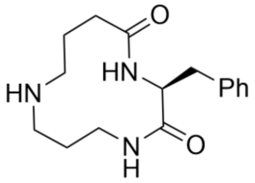

To a stirring suspension of lactam **19f** (11.5 mg, 0.0291 mmol) in water (0.5 mL) and acetonitrile (0.5 mL), periodic acid (7.0 mg, 0.0305 mmol) was added and stirred at room temperature for 18 hours. The reaction mixture was acidified to pH 1 with 0.2 M aqueous hydrochloric acid and washed with dichloromethane (2 × 10 mL). The aqueous layer was then adjusted to pH 11 with 1 M aqueous NaOH and extracted with a 3:1 mixture of chloroform:isopropanol (3 × 10 mL). The combined organic layers were dried with magnesium sulfate, filtered, concentrated under vacuum and purified via flash column chromatography (19:1 dichloromethane:methanol) to afford the title compound as a colourless oil (6.5 mg, 0.0225 mmol, 77%). R_f 0.28 (9:1 dichloromethane:methanol). ν_{max} (thin film)/cm^{-1} 3282, 3029, 2926, 1645, 1553, 1443, 1279, 1121, 700. ^{1}H NMR (400 MHz, d_4-MeOD) δ 7.33–7.14 (5H, m, PhH), 4.56 (1H, dd, *J* = 11.7, 3.4 Hz, CH), 3.60 (1H, dt, *J* = 13.6, 4.7 Hz, CH_2), 3.45 (1H, dd, *J* = 14.4, 3.4 Hz, CH_2), 3.09 (1H, ddd, *J* = 13.6, 10.2, 4.2 Hz, CH_2), 2.91 (1H, dd, *J* = 11.9, 5.6 Hz), 2.82–2.60 (3H, m, CH_2), 2.50 (1H, td, *J* = 11.0, 2.8 Hz, CH_2), 2.26–2.12 (2H, m, CH_2), 2.00–1.85 (1H, m, CH_2), 1.79–1.53 (3H, m, CH_2). ^{13}C NMR (101 MHz, d_4-MeOD) δ 176.8 (C), 173.3 (C), 139.6 (C), 129.9 (2 × CH), 129.4 (2 × CH), 127.5 (CH), 56.2 (CH), 50.8 (CH_2), 50.7 (CH_2), 42.0 (CH_2), 37.1 (CH_2), 36.3 (CH_2), 27.6 (CH_2), 26.6 (CH_2). HRMS (ESI$^+$) $C_{16}H_{24}N_3O_2$ (MH^+) theoretical 290.1863; measured 290.1865 (−0.6 ppm error); $C_{16}H_{23}N_3NaO_2$ (MNa^+); theoretical 312.1682; measured 312.1687 (−1.3 ppm error).

(*S*)-2-Benzyl-8-(furan-3-carbonyl)-1,4,8-triazacyclododecane-3,12-dione (24)

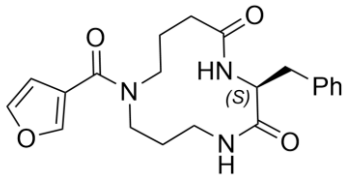

To a stirring suspension of lactam **23** (5.0 mg, 0.0173 mmol), 3-furoic acid (2.3 mg, 0.0208 mmol) and diisopropyl ethylamine (0.0181 mL, 0.104 mmol) in dry dichloromethane (1.0 mL) was added T3P (0.0154 mL, 0.0260 mmol, 50 wt% in ethyl acetate) and stirred at room temperature for 24 hours. Saturated aqueous sodium hydrogen carbonate (10 mL) was then added to the reaction mixture and extracted with a 3:1 mixture of chloroform:isopropanol (3 × 10 mL). The combined organic layers were dried with magnesium sulfate, filtered, concentrated under vacuum and purified via flash column chromatography (97:3 →77% 20:1 methanol:dichloromethane) to afford the title compound as a white solid (5.1 mg, 0.0133 mmol, 77%). Mp 259–261 °C. R_f 0.54 (9:1 dichloromethane:methanol). ν_{max}

(thin film)/cm^{-1} 3289, 2926, 1636, 1510, 1439, 1222, 1181, 701. Note: both the ^{1}H and ^{13}C NMR spectra obtained for this product showed severe broadening of their NMR signals due to rotamers (1:1:1:1 ratio). ^{1}H NMR (700 MHz, CD_3OD) δ 7.88 (1H, t, *J* = 1.0 Hz, ArH), 7.58 (1H, t, *J* = 1.8 Hz, ArH), 7.29–7.24 (4H, m, PhH), 7.23–7.16 (1H, m, PhH), 6.63 (1H, d, *J* = 1.8 Hz, ArH), 4.74 (1H, t, *J* = 7.8 Hz, CH), 3.63–2.97 (4H, m, CH_2), 2.91 (1H, dd, *J* = 14.0, 7.8 Hz, CH_2), 2.80–2.64 (1H, m, CH_2), 2.37–1.50 (8H, m, CH_2). (400 MHz, d_6-DMSO) 8.81 (1H, d, *J* = 9.1 Hz, NH), 8.04–7.99 (1H, m, ArH), 7.79–7.65 (2H, m, ArH, NH), 7.31–7.15 (5H, m, PhH), 6.64 (1H, d, *J* = 2.0 Hz, ArH), 4.53 (1H, q, *J* = 8.3 Hz, CH), 3.43–3.15 (1H, m, CH_2), 3.13–2.78 (4H, m, CH_2), 2.73–2.59 (1H, m, CH_2), 2.23–2.08 (1H, m, CH_2), 2.06–1.38 (7H, m, CH_2). ^{13}C NMR (176 MHz, CD_3OD) δ 173.9 (CO, all rotamers), 166.8 (CO, all rotamers), 144.8 (ArH, all rotamers), 144.6 (ArH, all rotamers), 138.8 (PhC, all rotamers), 130.2 (2 × PhCH, all rotamers), 129.5 (2 × PhCH, all rotamers), 127.6 (PhCH, all rotamers), 122.0 (ArC, all rotamers), 110.9 (ArH, all rotamers), 55.9 (CH, all rotamers), 46.4 (CH_2), 45.4 (CH_2), 43.2 (CH_2), 42.1 (CH_2), 37.1 (CH_2, all rotamers), 35.8 (CH_2, all rotamers), 34.7, (CH_2, all rotamers), 33.1 (CH_2), 30.5 (CH_2), 29.0 (CH_2), 28.1 (CH_2), 27.3 (CH_2), 26.9 (CH_2), 25.4 (CH_2), 23.7 (CH_2), HRMS (ESI$^+$) $C_{21}H_{25}N_3NaO_4$ (MNa^+) theoretical 406.1737; measured 406.1748 (–2.5 ppm error); $C_{21}H_{25}KN_3O_4$ (MK^+), theoretical 422.1477; measured 422.1485 (–2.1 ppm error).

4) Comparison of isolated and synthetic material data

Comparison of the NMR data for our synthesised sample of **2** with the data reported in the publication in which celacarfurine was isolated showed clear differences. The NMR data in the isolation study was reported to be in d_4-MeOD. However, we found the synthesised compound **2** to have very low solubility in methanol and as a result, the d_4-MeOD NMR data obtained was very weak. It is possible, however, to compare the aromatic region of the ^{1}H NMR spectra. We were unable to obtain ^{13}C NMR data in d_4-MeOH of reasonable strength for comparison. Unfortunately, we discovered that the ^{1}H NMR data in the publication reporting the isolation celacarfurine[3] does not match the experimental data obtained. We observed up to a 0.14 ppm difference in furoyl signal (proton 3), as well as up to 0.17 ppm difference for the exocyclic phenyl ring (proton 4). We also observed a significant difference in splitting pattern for the phenyl ring protons.

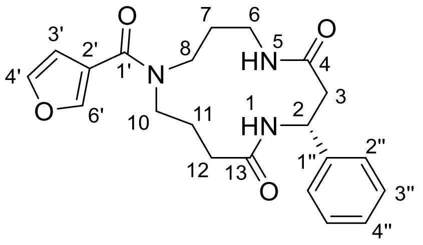

*Table S1: ^{1}H NMR comparison of **2** and isolated celacarfurine in d_4-MeOD.*

Atom	δ_H (isolated) / ppm literature data[3]	δ_H (synthetic **2**) / ppm	Difference / ppm
3’	6.66 (s (br))	6.65 (s)	−0.01
4’	7.73 (s (br))	7.59 (s)	−0.14
6’	8.02 (s)	7.89 (s)	−0.13
2’’	7.52 (dd, *J* = 8.0, 1.8 Hz)	7.42–7.30 (m)	~−0.17
3’’	7.36–7.32 (m)	7.42–7.30 (m)	~+0.01
4’’	7.36–7.32 (m)	7.29–7.21 (m)	~−0.09

We later discovered that the same compound was subsequently described in a patent.[4] As we described in the manuscript, analysis of the data in the patent led us to the conclusion that the ^{1}H and ^{13}C NMR in the isolation paper[3] were like reported as being in d_4- methanol in error. All NMR signals described in the patent are identical to those in the isolation paper, but crucially, the patent includes images of the ^{1}H and ^{13}C NMR spectra in which residual solvent signals for d_6-DMSO, not d_4-methanol, are clearly visible. We therefore characterised **2** in d_6-DMSO instead to compare their data. Our synthetic sample dissolved well in d_6-DMSO, and the NMR data supports the assigned 13-membered

macrocyclic structure. But unfortunately, major differences were evident when comparing the synthetic and isolated materials – see Tables S2 and S3 for selected signals showing clear differences. The ^{13}C and ^{1}H NMR spectra also show splitting of some signals due to rotamers (4, 6–8, 10–13 and 1'), which is often seen from macrocycles which contain amide bonds. The presence of rotamers is not reported in the isolated material, which represents another important difference.

*Table S2: ^{1}H NMR comparison of **2** and isolated celacarfurine in d_6-DMSO.*

Atom	δ_H (isolated) / ppm literature data[4]	δ_H (synthetic **2**) / ppm	Difference / ppm
1	-	8.54–8.39	-
2	5.57 (m)	5.30 (t, *J* = 9.7 Hz)	−0.27
3	3.45 (dd, *J* = 12.5, 8.5 Hz), 2.41 (dd, *J* = 12.5, 7.8 Hz)	2.63–2.53 (m), 2.49–2.44 (m)	~−0.87, ~−0.06
5	-	7.94 (1H, t, *J* = 5.7 Hz)	-
6	3.46 (m), 3.05 (m)	3.30–3.20 (m), 3.01–2.83	~−0.21, ~−0.13
7	3.49 (m), 2.67 (m)	2.08–1.68 (m), 1.67–1.57 (m),	~−1.61, ~−1.05
8	3.51 (m), 2.82 (m)	3.68–3.53 (m), 3.50–3.42 (m), 3.30–3.20 (m), 3.18–3.08 (m)	-
10	3.68 (m), 2.90 (m)	3.68–3.53 (m), 3.50–3.42 (m), 3.01–2.83 (m), 2.80–2.69 (m)	-
11	2.09 (m), 1.55 (m)	2.08–1.68 (m)	-
12	3.73 (m), 2.67 (m)	2.38–2.25 (m), 2.08–1.68 (m)	~−1.42, ~−0.79
3'	6.66 (s (br))	6.66 (d, *J* = 6.8 Hz)	0.00
4'	7.73 (s (br))	7.73 (d, *J* = 11.1 Hz)	0.00
6'	8.02 (s)	8.02 (d, *J* = 10.1 Hz)	0.00
2''	7.52 (dd, *J* = 8.0, 1.8 Hz)	7.35–7.31 (m)	~−0.19
3''	7.36–7.32 (m)	7.35–7.31 (m)	~−0.01
4''	7.36–7.32 (m)	7.25–7.21 (m)	~−0.11

*Table S3: ^{13}C NMR comparison of **2** and isolated celacarfurine in d_6-DMSO.*

Atom	δ_H (isolated) / ppm literature data[4]	δ_H (synthetic **2**) / ppm	Difference / ppm
2	73.7	50.4	−23.3
3	38.8	43.2	+4.4
4	169.3	169.9, 169.9	−0.6
6	44.4	35.9	−8.5
7	36.1	27.1, 25.7	-
8	46.5	45.3, 41.2	-
10	41.1	45.6, 42.4	-
11	26.5	25.1, 23.3	-
12	43.3	33.4, 33.1	-
13	169.3	170.8, 170.7	-
1’	163.4	163.4, 163.5	-
2’	121.1	121.2	+0.1
3’	110.5	110.5	0.0
4’	143.3	143.3	0.0
6’	142.9	143.0	0.1
1’’	137.3	143.4	+6.1
2’’	127.7	126.1	−1.6
3’’	128.2	128.4	+0.2
4’’	128.4	126.8	−1.6

The ^{1}H NMR signals are all relatively broad, which is commonly seen in lactam-containing medium-sized rings and small macrocycles of this type. We carried out COSY, HSQC and HMBC experiments to assign **2** (see section 6 for images of all NMR spectra and 2D NMR data). The observed HMBC correlations are depicted in Figure S1. All observed COSY correlations were also consistent with the depicted structure of **2**.

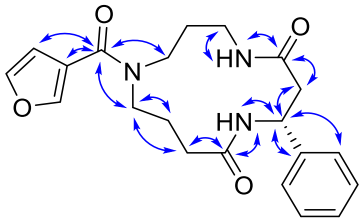

*Figure S1: HMBC correlations of **2**.*

We carried out a comparison of the optical rotation of our synthesised **2** ($[\alpha]_d$ = +42.42) to the reported optical rotation value for the isolated material ($[\alpha]_d$ = +5.78) and found a large difference in the measured rotation, although notably both show a positive optical rotation value which is the opposite to that of celafurine ($[\alpha]_d$ = −10.1) as measured by Hesse and co-workers.[5]

5) Encapsulated nanodroplet crystallisation (ENaCt) attempts on macrocycle 2

Chemical formula: $C_{21}H_{25}N_3O_4$

Proposed structure:

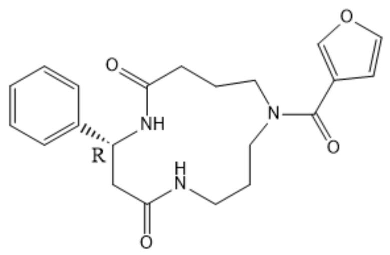

Procedure:

The supplied compound, **2**, was suspended in 1.6 mL of methanol. The resulting pale cloudy solution was dispensed evenly across 16 vials (100 µL each) and the solutions were allowed to evaporate to dryness overnight. Each vial was assumed to contain approximately 1 mg of **2** and the stock solutions for the following solvents were prepared using the volumes outlined below. Asterisks denote where the compound was not fully soluble, so the supernatant was used in the ENaCt experiments.

Solvent	Volume added / µL	Approximate concentration / mg mL^{-1}
THTP-1-oxide*	200	5.0
1,4-dioxane*	200	5.0
Chlorobenzene (PhCl)*	200	5.0
Diethylformamide (DEF)*	200	5.0
N-methyl pyrrolidinone (NMP)*	200	5.0
Diethylsulfoxide (DESO)*	200	5.0
Tetrahydrofuran (THF)*	200	5.0
Ethyl acetate (EtOAc)*	200	5.0
Dimethylformamide (DMF)*	200	5.0
1:1 DMF : THF*	200	5.0
1:1 DMF : PhCl*	200	5.0
1:1 DMF : DMSO*	200	5.0

Plate layout:

Plate number														
			**Volume of Oil (200 nL)**											
	**Volume of Solvent (50 nL)**		**1**	**2**	**3**	**4**	**5**	**6**	**7**	**8**	**9**	**10**	**11**	**12**
1	Solvent A	**A**	No oil	PDMSO					No oil	Fomblin-Y				
		**B**	No oil	FC-40					No oil	Mineral oil				
2	Solvent B	**C**	No oil	PDMSO					No oil	Fomblin-Y				
		**D**	No oil	FC-40					No oil	Mineral oil				
3	Solvent C	**E**	No oil	PDMSO					No oil	Fomblin-Y				
		**F**	No oil	FC-40					No oil	Mineral oil				
4	Solvent D	**G**	No oil	PDMSO					No oil	Fomblin-Y				
		**H**	No oil	FC-40					No oil	Mineral oil				

ENaCt Classifications:
F – robotic failure
1 – still solvated / no solid material
2 – amorphous / non-crystalline material
3 – microcrystalline material / small single crystals
4 – single crystals likely suitable for in-house SCXRD

Plate readouts after 14 days:

**Plate 1**														
	**Solvent**		**1**	**2**	**3**	**4**	**5**	**6**	**7**	**8**	**9**	**10**	**11**	**12**
**1**	THTP-1-oxide	**A**	2	2	2	2	2	2	2	2	2	2	2	2
	THTP-1-oxide	**B**	2	2	2	2	2	2	2	2	2	2	2	2
**2**	1,4-dioxane	**C**	2	2	2	2	2	2	2	2	2	2	2	2
	1,4-dioxane	**D**	2	2	2	2	2	2	2	2	2	2	2	2
**3**	PhCl	**E**	2	2	2	2	2	2	2	2	2	2	2	2
	PhCl	**F**	2	2	2	2	2	2	2	2	2	2	2	2
**4**	DEF	**G**	2	2	2	2	2	2	2	2	2	2	2	2
	DEF	**H**	2	2	2	2	2	2	2	2	2	2	2	2

**Plate 2**														
	**Solvent**		**1**	**2**	**3**	**4**	**5**	**6**	**7**	**8**	**9**	**10**	**11**	**12**
**1**	NMP	**A**	2	2	2	2	2	2	2	2	2	2	2	2
	NMP	**B**	2	2	2	2	2	2	2	2	2	2	2	2
**2**	DESO	**C**	2	2	2	2	2	2	2	2	2	2	2	2
	DESO	**D**	2	2	2	2	2	2	2	2	2	2	2	2
**3**	THF	**E**	2	2	2	2	2	2	2	2	2	2	2	2
	THF	**F**	2	2	2	2	2	2	2	2	2	2	2	2
**4**	EtOAc	**G**	2	2	2	2	2	2	2	2	2	2	2	2
	EtOAc	**H**	2	2	2	2	2	2	2	2	2	2	2	2

Plate 3														
	**Solvent**		**1**	**2**	**3**	**4**	**5**	**6**	**7**	**8**	**9**	**10**	**11**	**12**
**1**	DMF	**A**	2	2	2	2	2	2	2	2	2	2	2	2
	DMF	**B**	2	2	2	2	2	2	2	2	2	2	2	2
**2**	1:1 DMF : THF	**C**	2	2	2	2	2	2	2	2	2	2	2	2
	1:1 DMF : THF	**D**	2	2	2	2	2	2	2	2	2	2	2	2
**3**	1:1 DMF : PhCl	**E**	2	2	2	2	2	2	2	2	2	2	2	2
	1:1 DMF : PhCl	**F**	2	2	2	2	2	2	2	2	2	2	2	2
**4**	1:1 DMF : DMSO	**G**	2	2	2	2	2	2	2	2	2	2	2	2
	1:1 DMF : DMSO	**H**	2	2	2	2	2	2	2	2	2	2	2	2

Conclusions:

No crystals suitable for SCXRD studies were obtained from the ENaCt screen of **2**, which has a tendency to produce amorphous material from the crystallisation conditions tested by ENaCt outlined above.

6) ^{1}H and ^{13}C spectra

***tert*-Butyl (3-(benzylamino)propyl)carbamate** (**10a**) – in $CDCl_3$; 400 MHz for ^{1}H, 101 MHz for ^{13}C

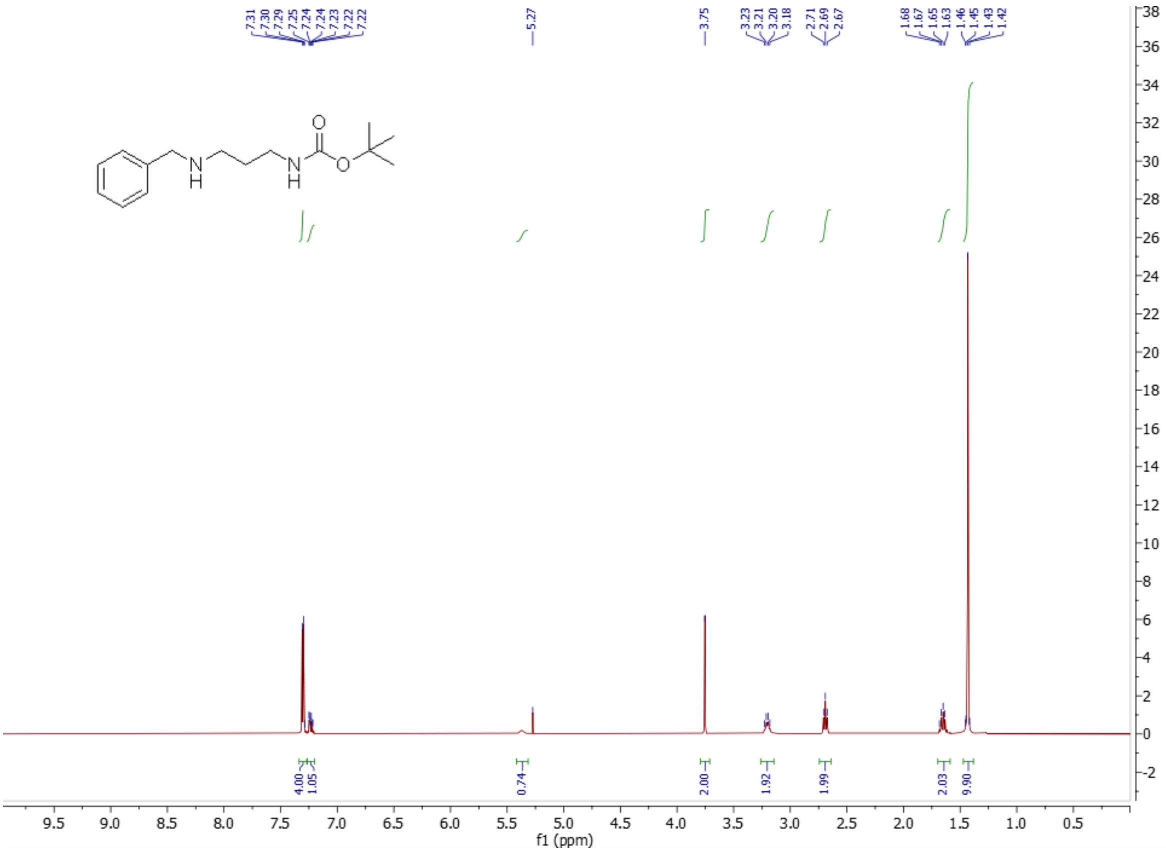

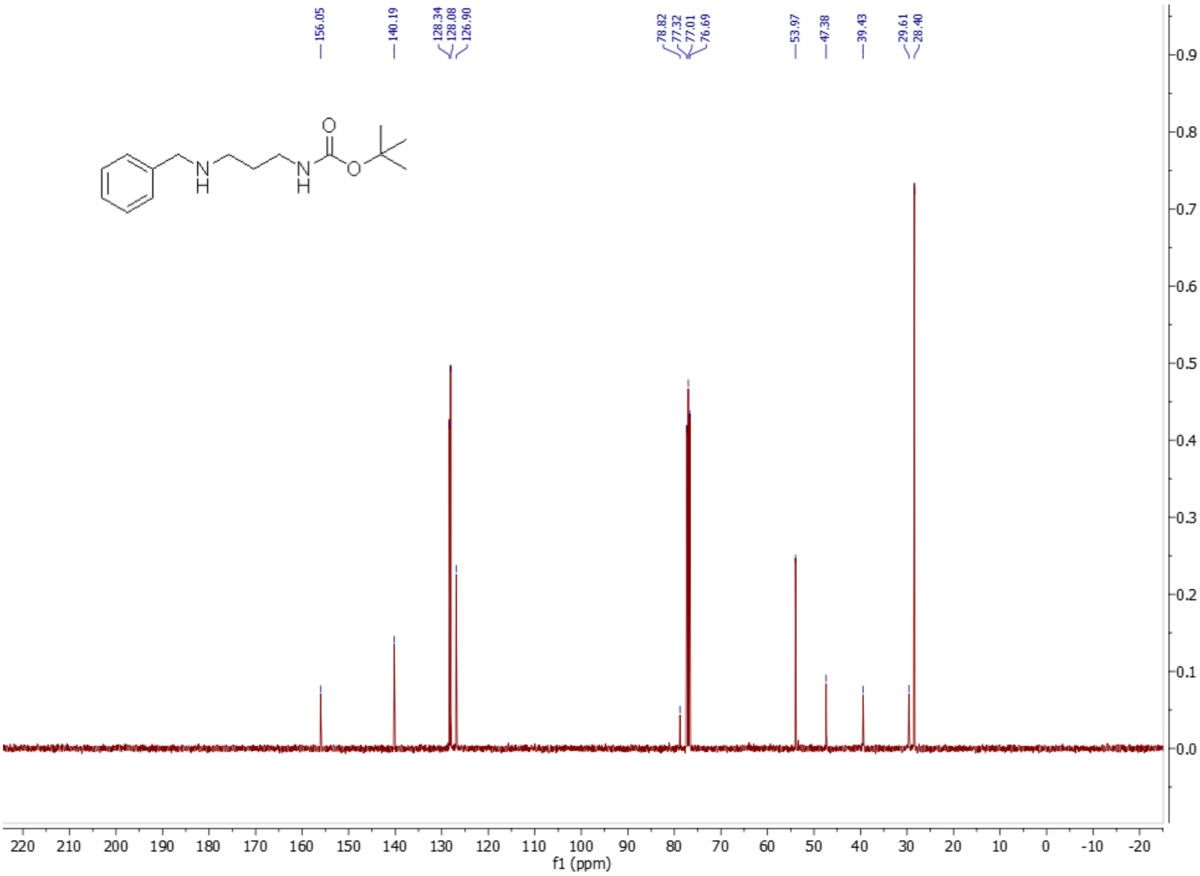

***tert*-Butyl (3-((4-methoxybenzyl)amino)propyl)carbamate** (**10b**) – in $CDCl_3$; 400 MHz for ^1H, 101 MHz for ^{13}C

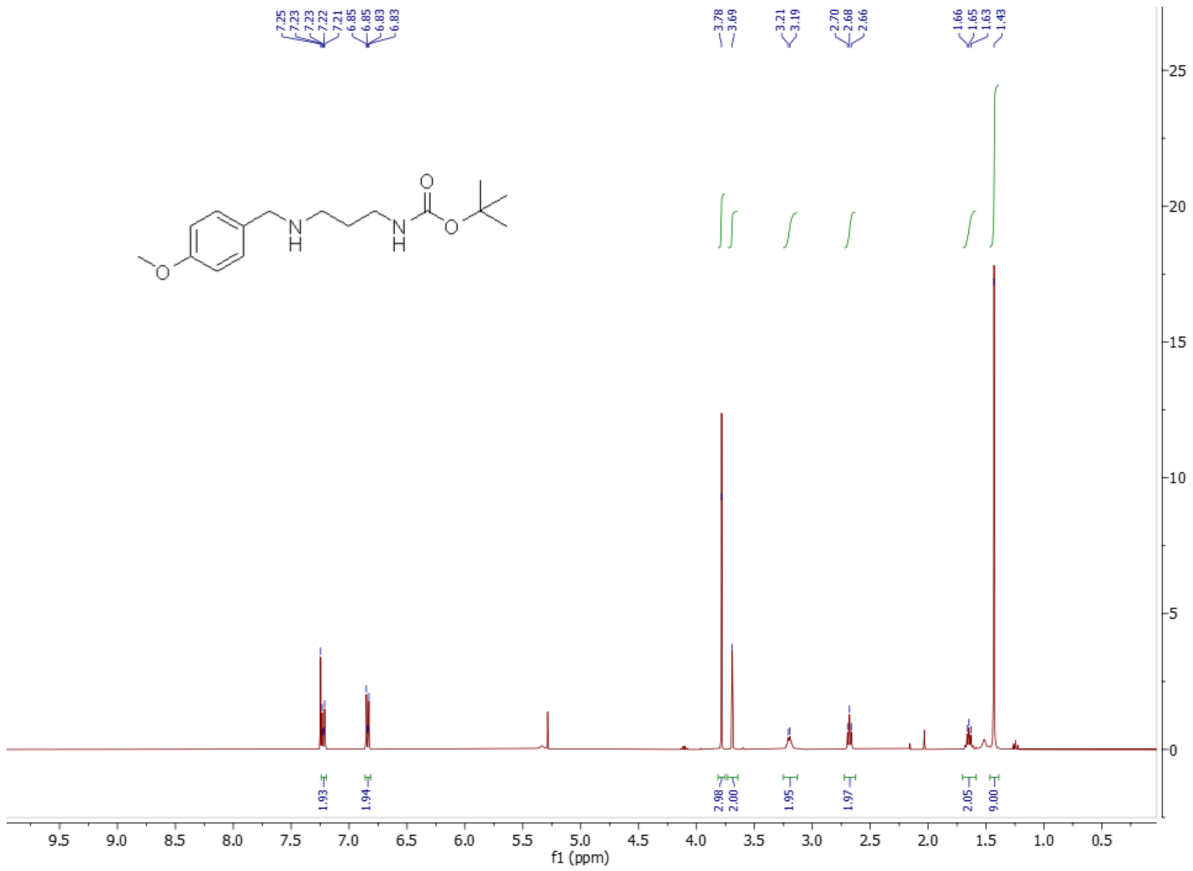

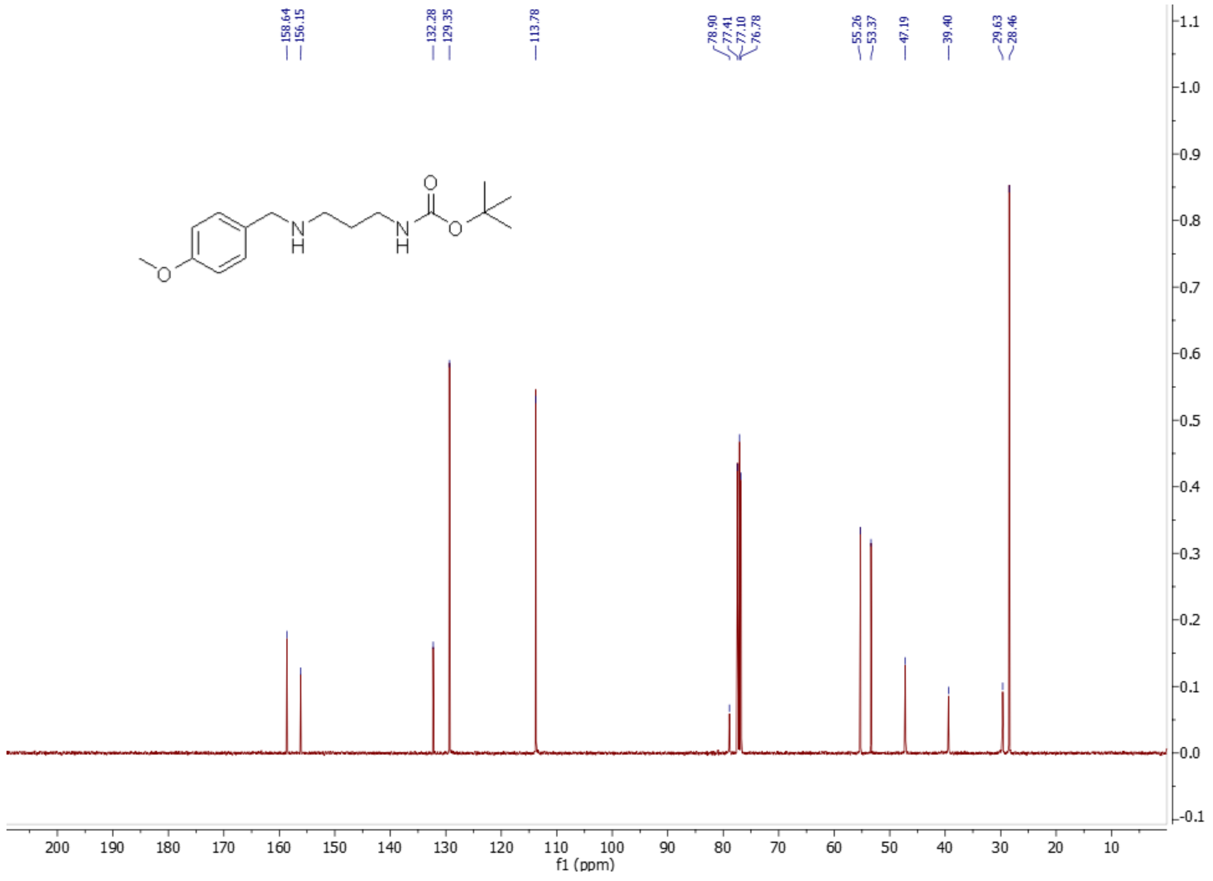

***tert*-Butyl (3-((4-methoxyphenyl)amino)propyl)carbamate (10c)** – in $CDCl_3$; 400 MHz for ^1H, 101 MHz for ^{13}C

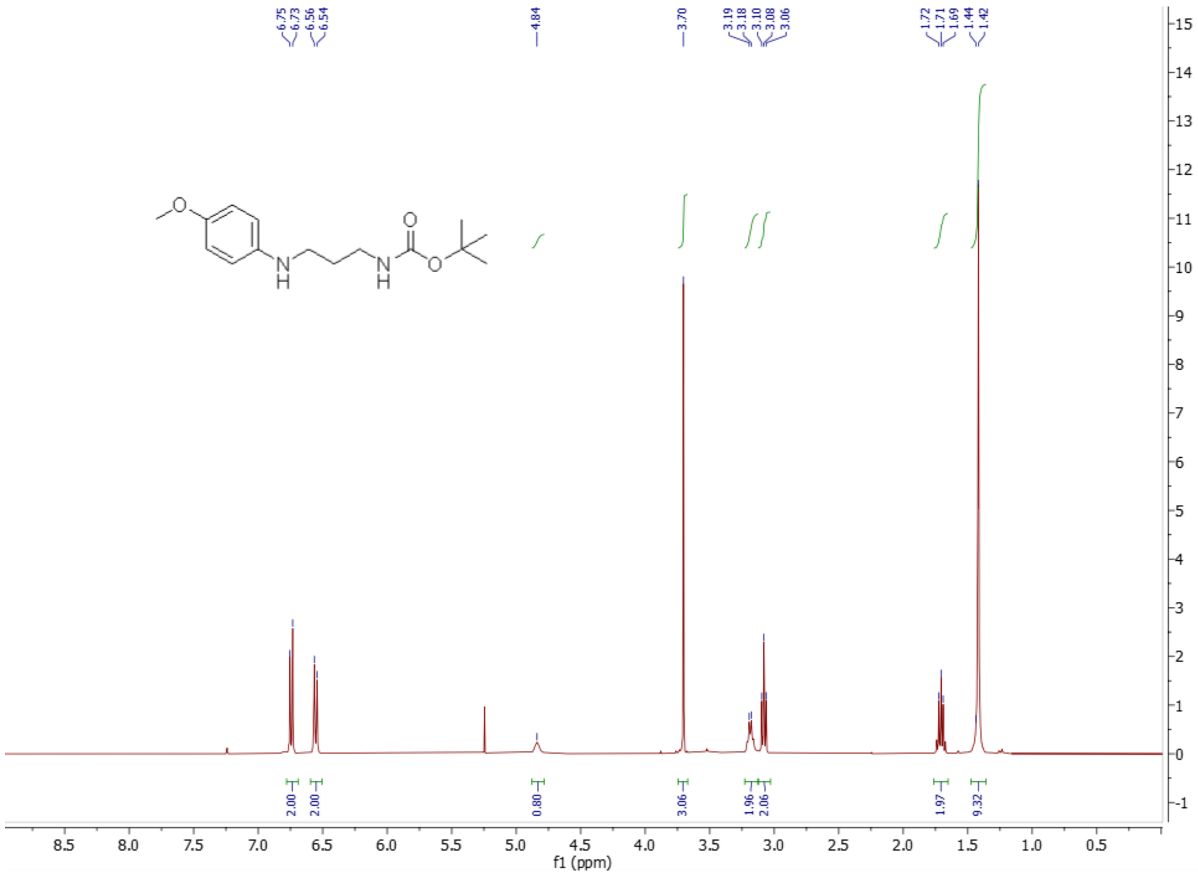

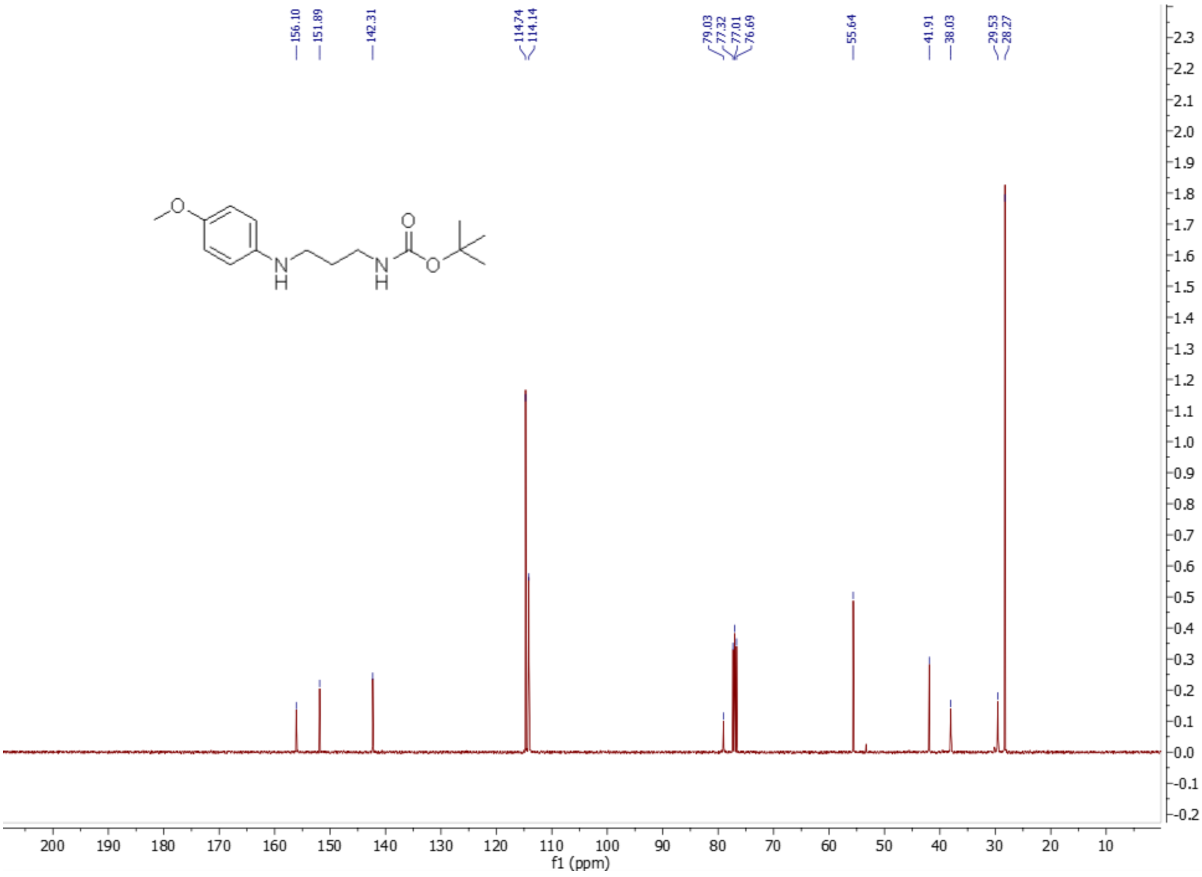

***tert*-Butyl 4-(benzyl(3-((*tert*-butoxycarbonyl)amino)propyl)amino)butanoate (11a)** – in $CDCl_3$; 400 MHz for ^1H, 101 MHz for ^{13}C

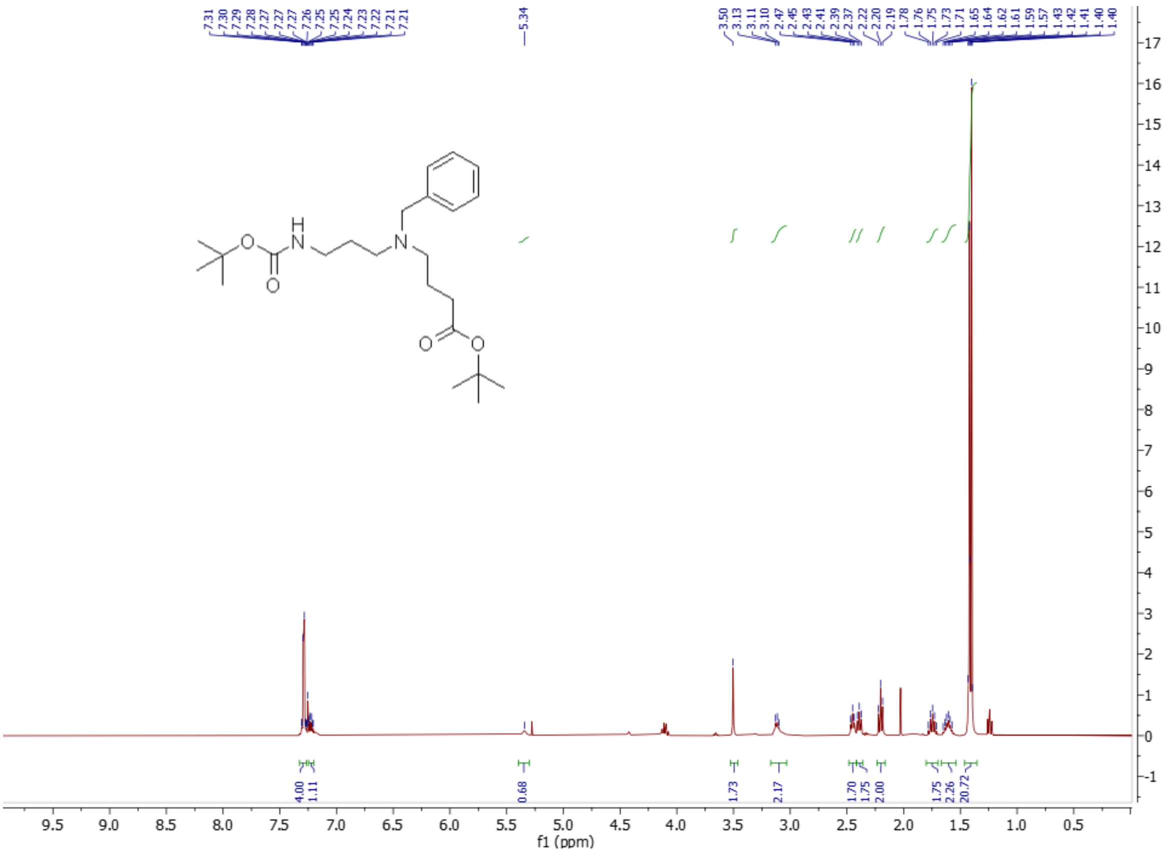

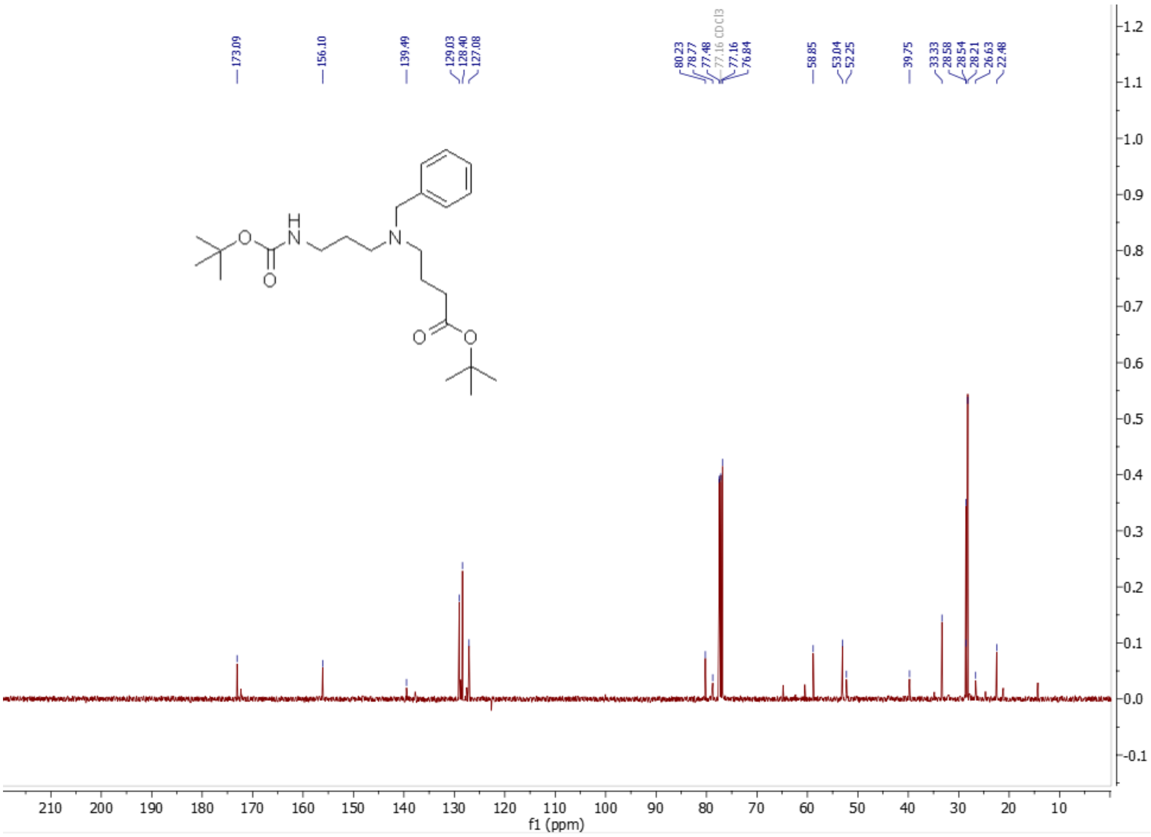

***tert*-Butyl 4-((3-((*tert*-butoxycarbonyl)amino)propyl)(4-methoxybenzyl)amino)butanoate** (**11b**) – in $CDCl_3$; 400 MHz for ^1H, 101 MHz for ^{13}C

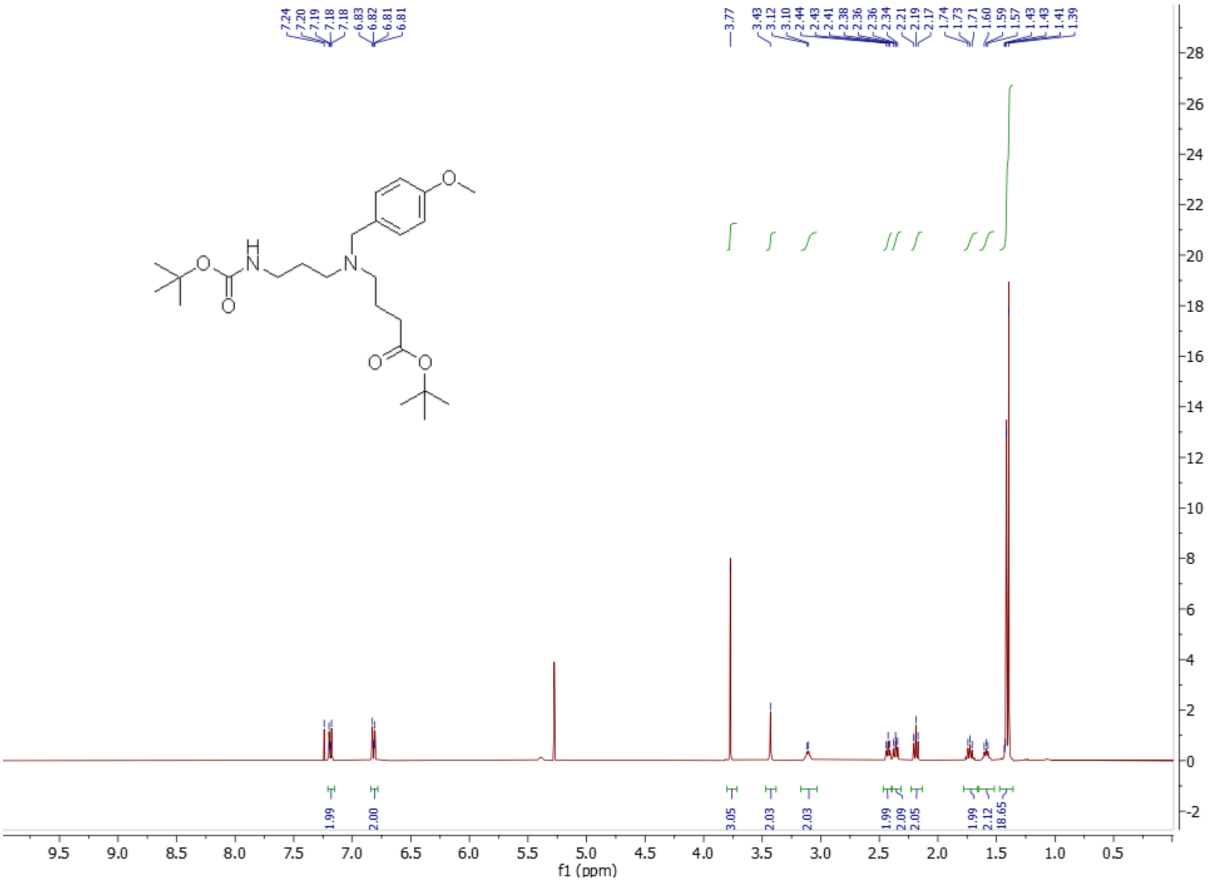

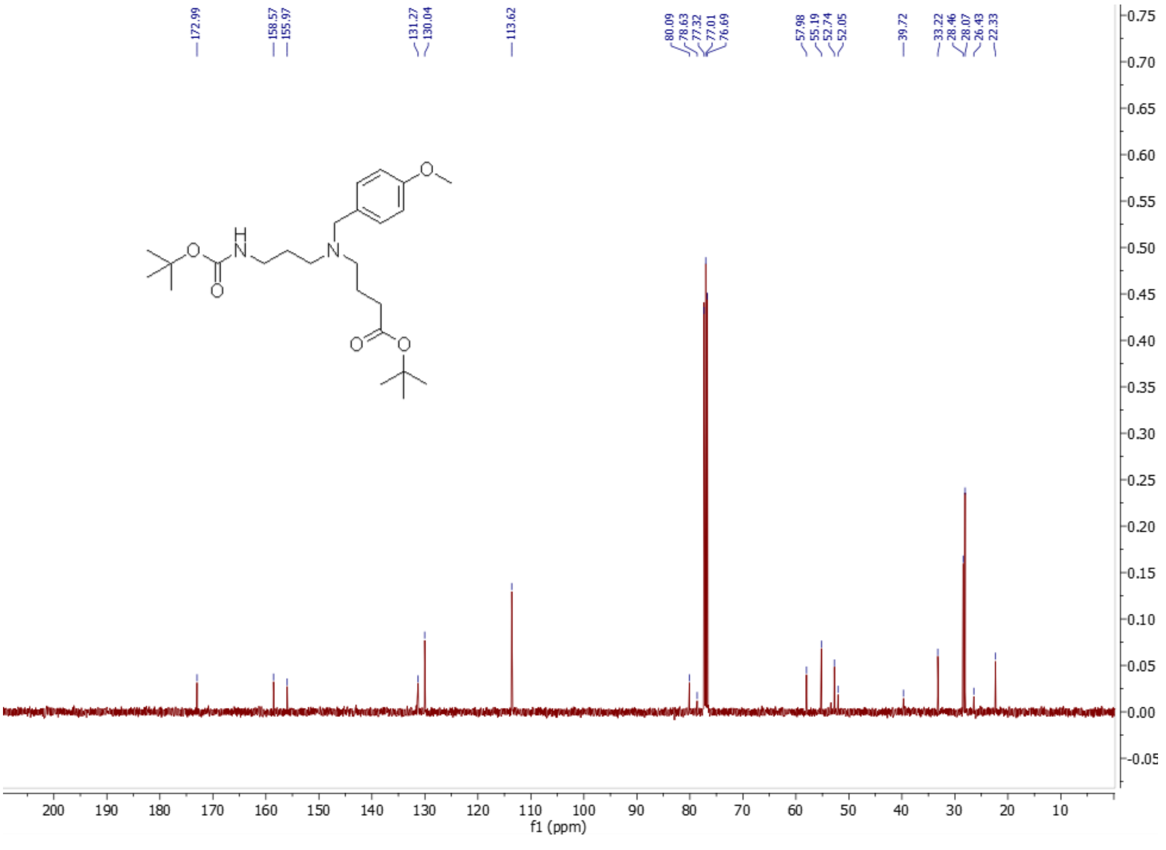

***tert*-Butyl 4-((3-((*tert*-butoxycarbonyl)amino)propyl)(4-methoxyphenyl)amino)butanoate (11c)** – in $CDCl_3$; 400 MHz for ^{1}H, 101 MHz for ^{13}C

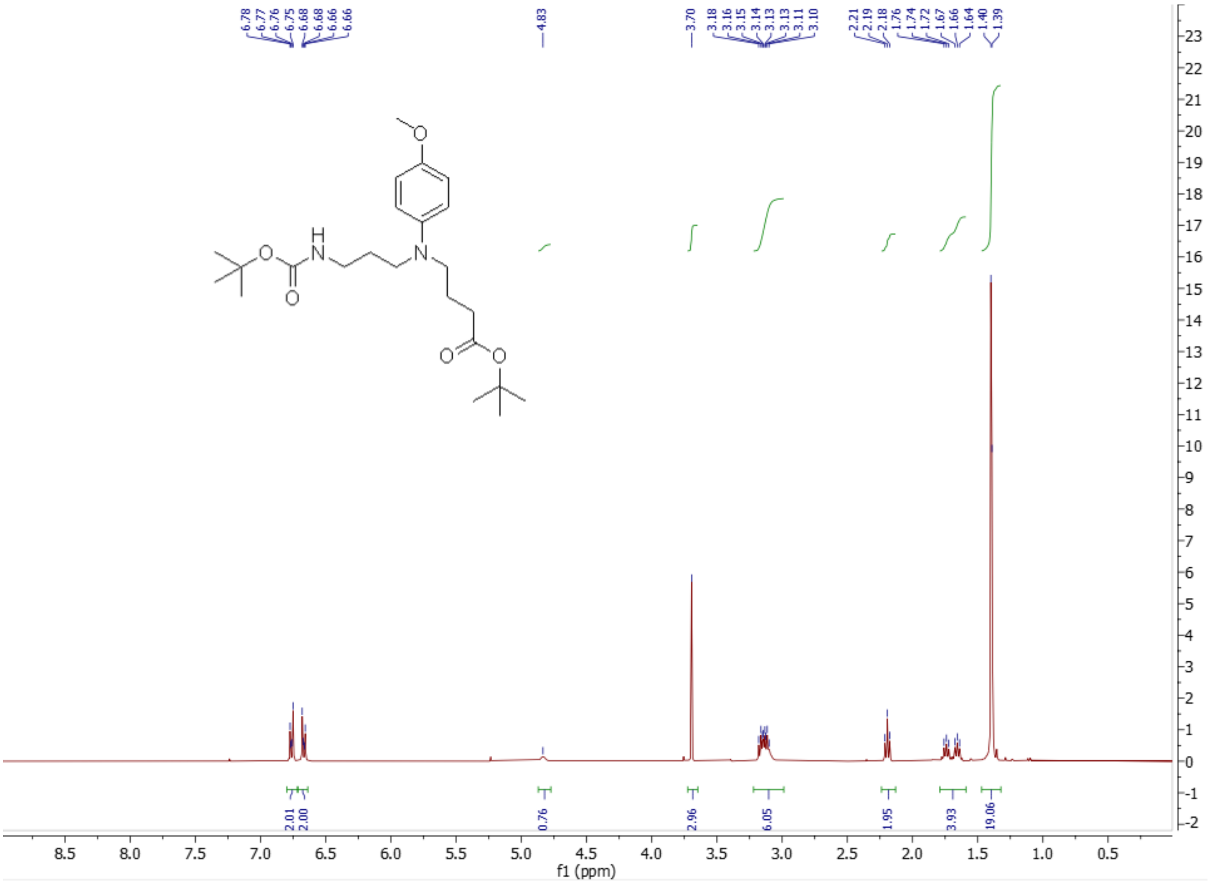

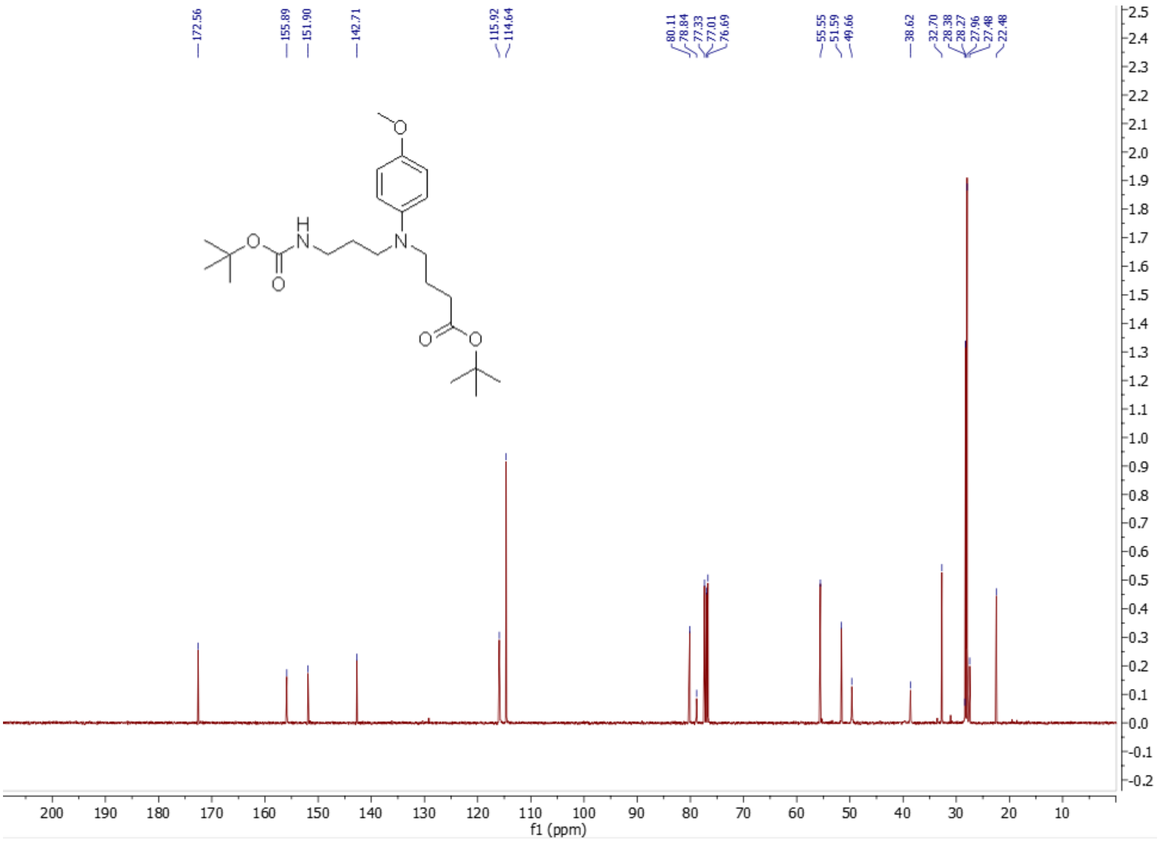

1-Benzyl-1,5-diazonan-6-one (**13a**) – in $CDCl_3$; 400 MHz for ^{1}H, 101 MHz for ^{13}C

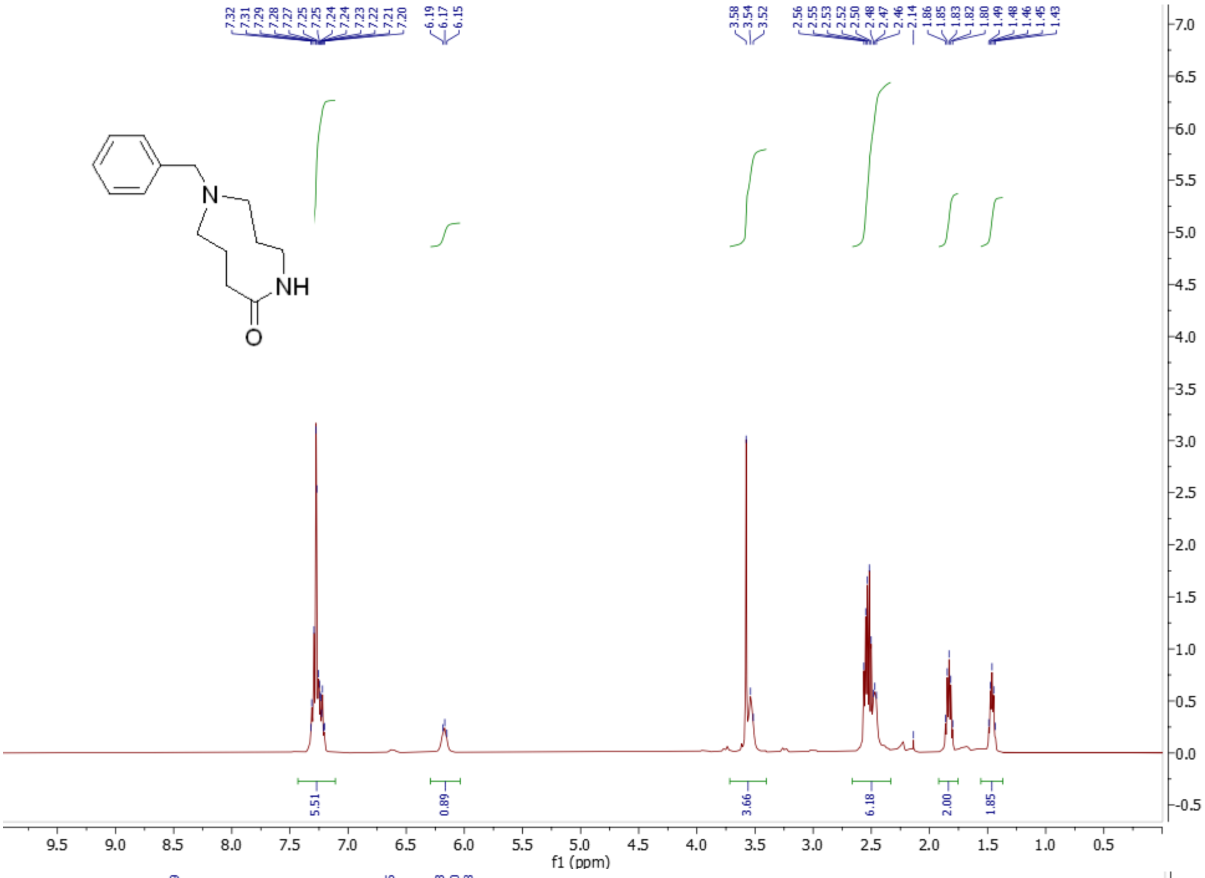

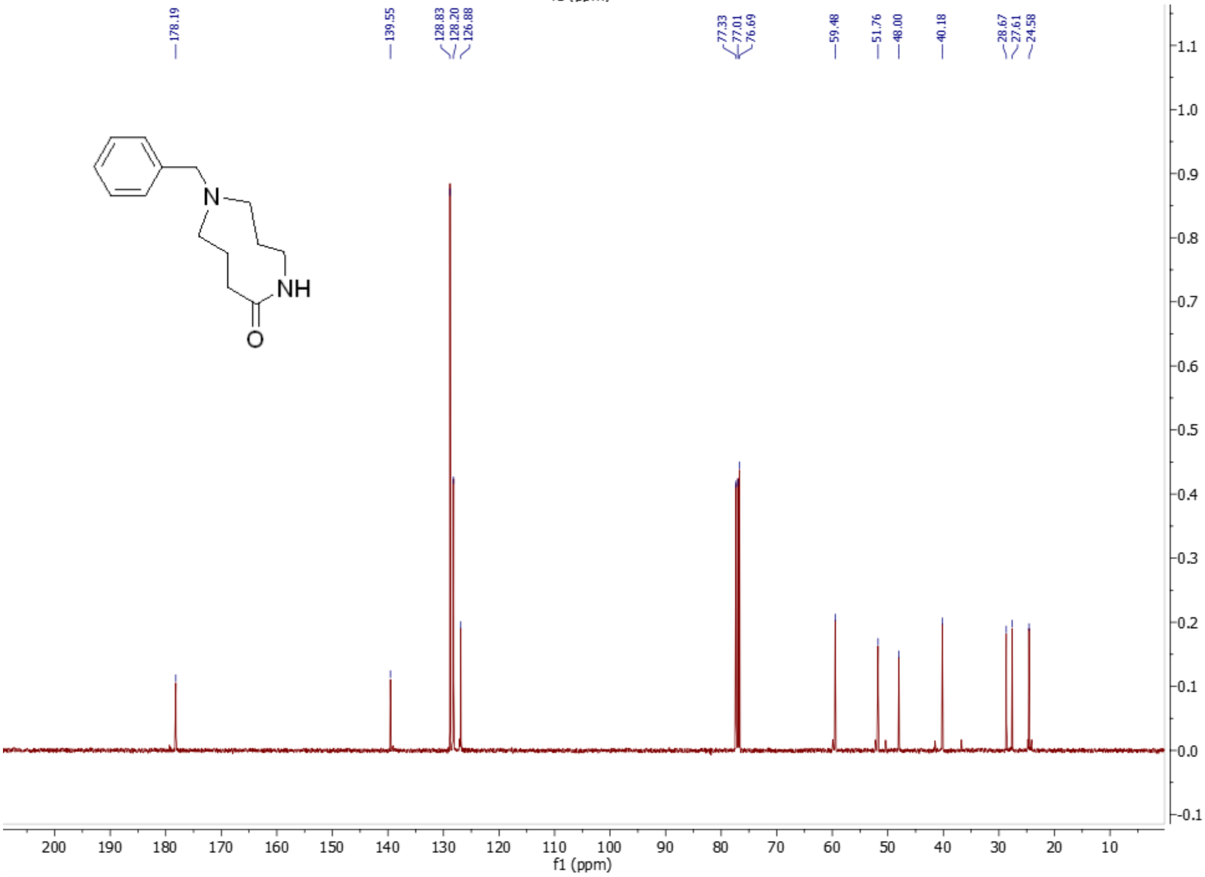

1-(4-Methoxybenzyl)-1,5-diazonan-6-one (**13b**) – in $CDCl_3$; 400 MHz for ^{1}H, 101 MHz for ^{13}C

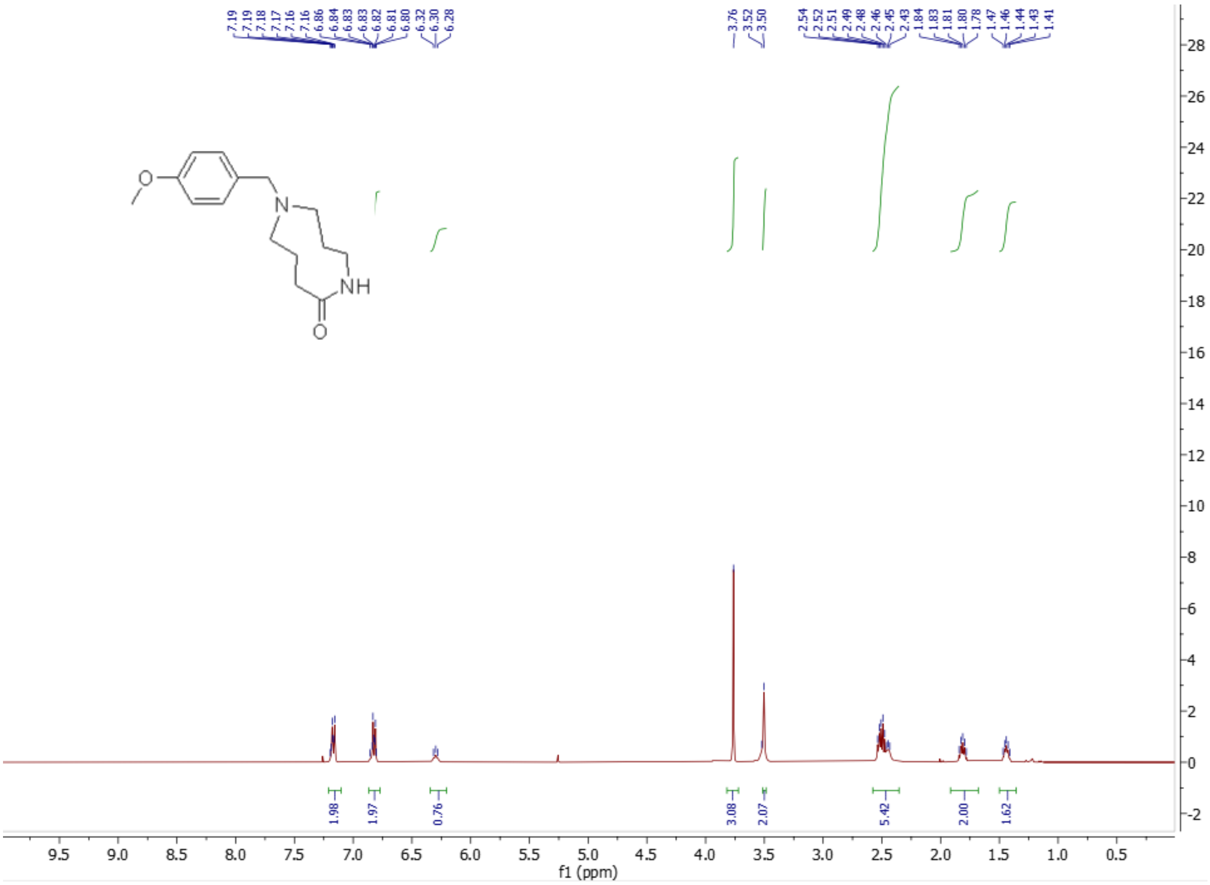

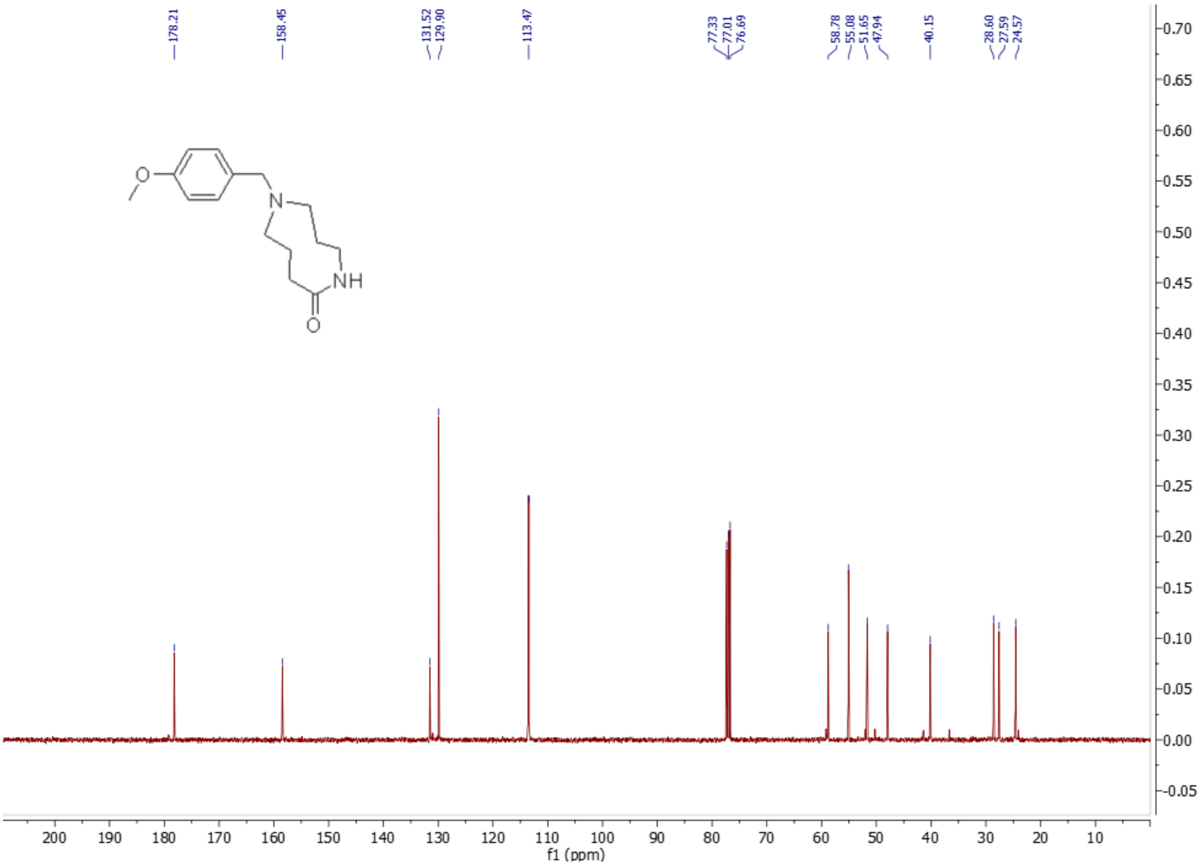

1-(4-Methoxyphenyl)-1,5-diazonan-6-one (**13c**) – in $CDCl_3$; 400 MHz for ^{1}H, 101 MHz for ^{13}C

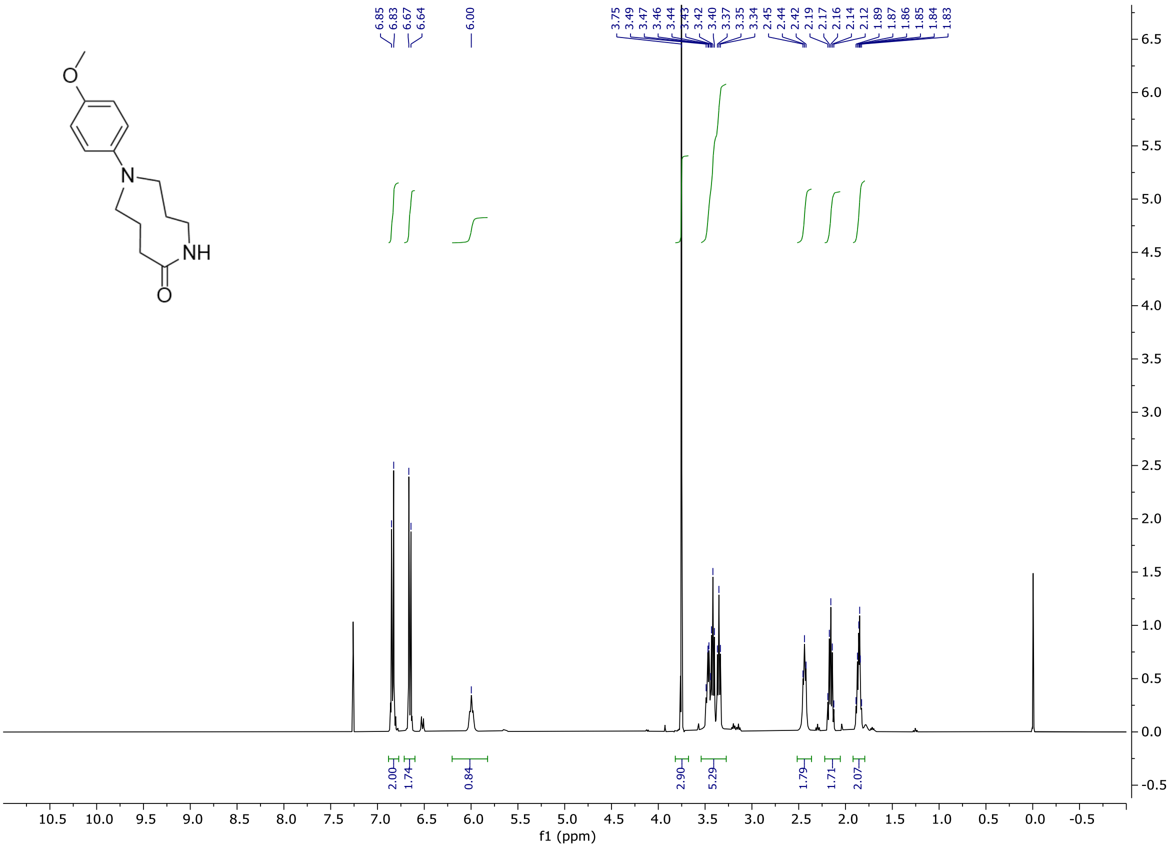

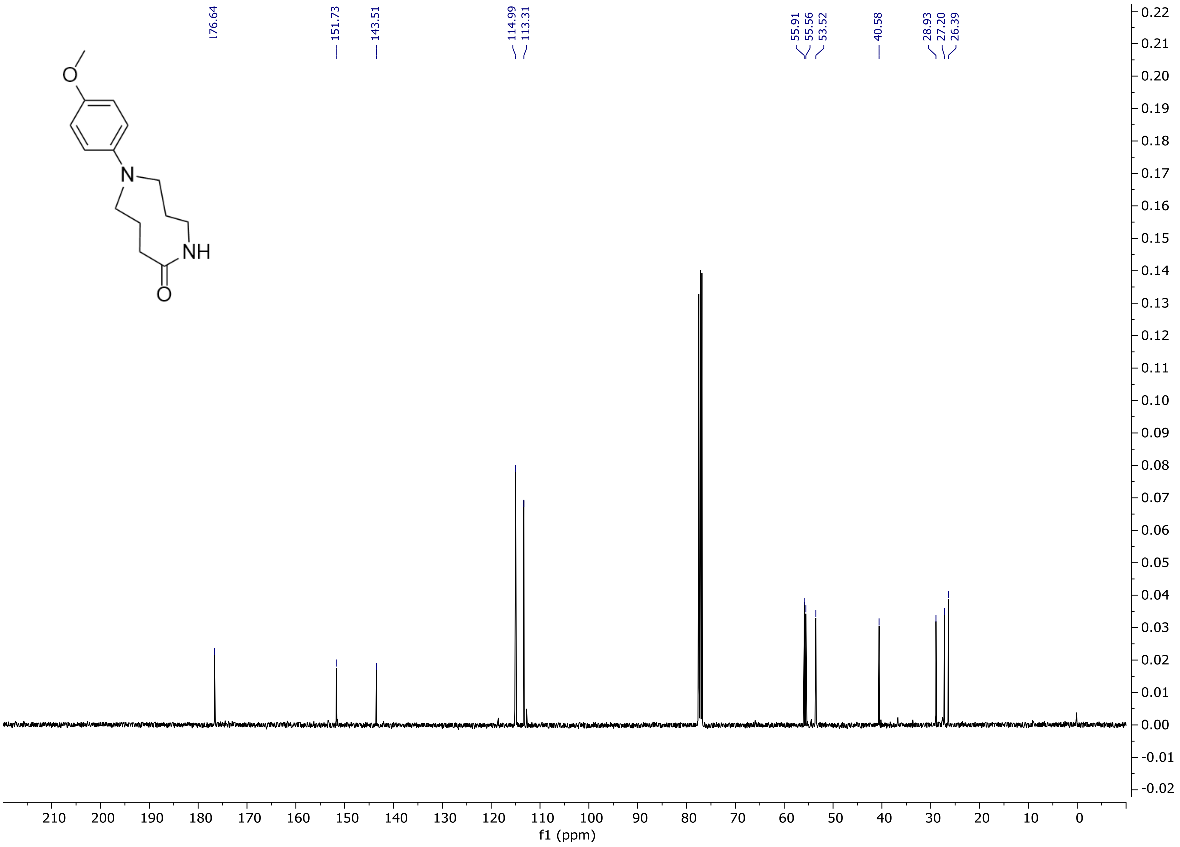

5-Acryloyl-1-benzyl-1,5-diazonan-6-one (**14**) – in $CDCl_3$; 400 MHz for ^1H, 101 MHz for ^{13}C

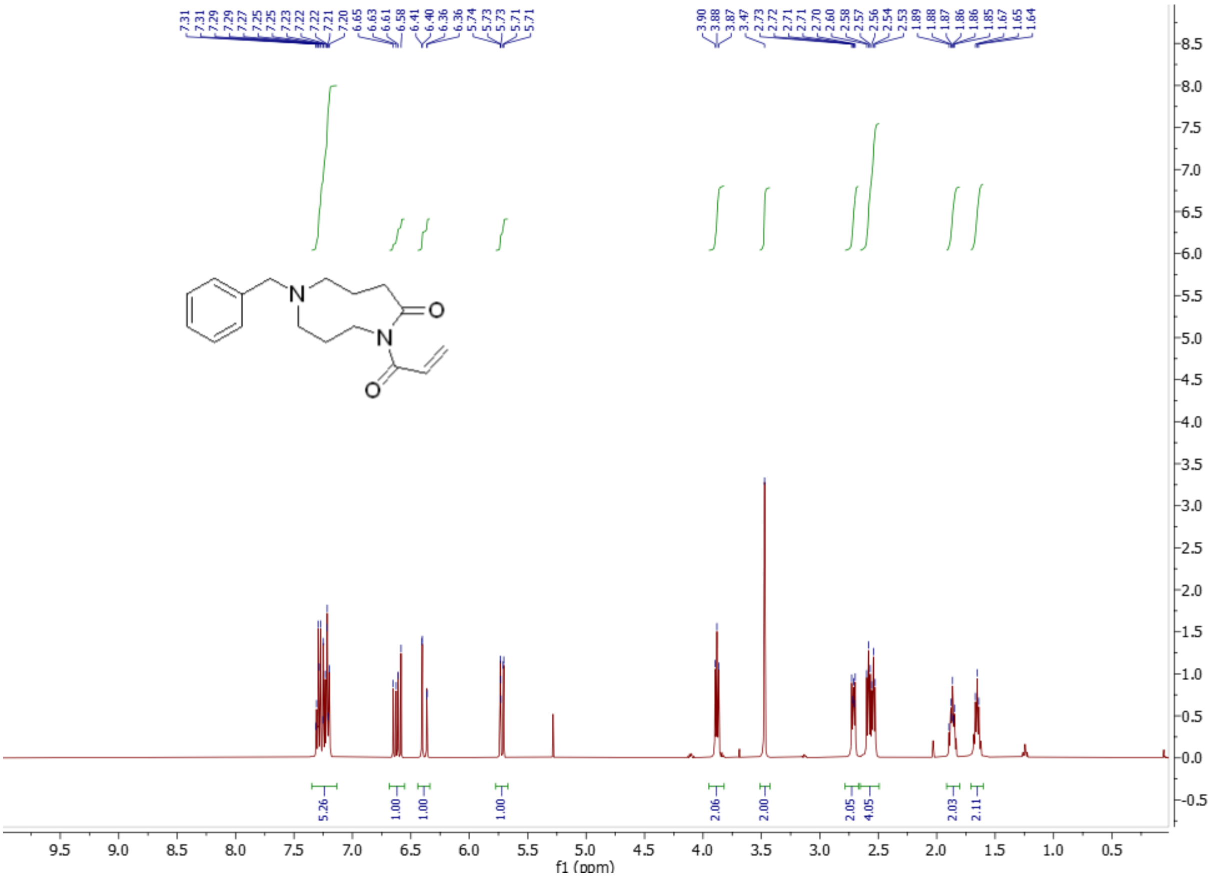

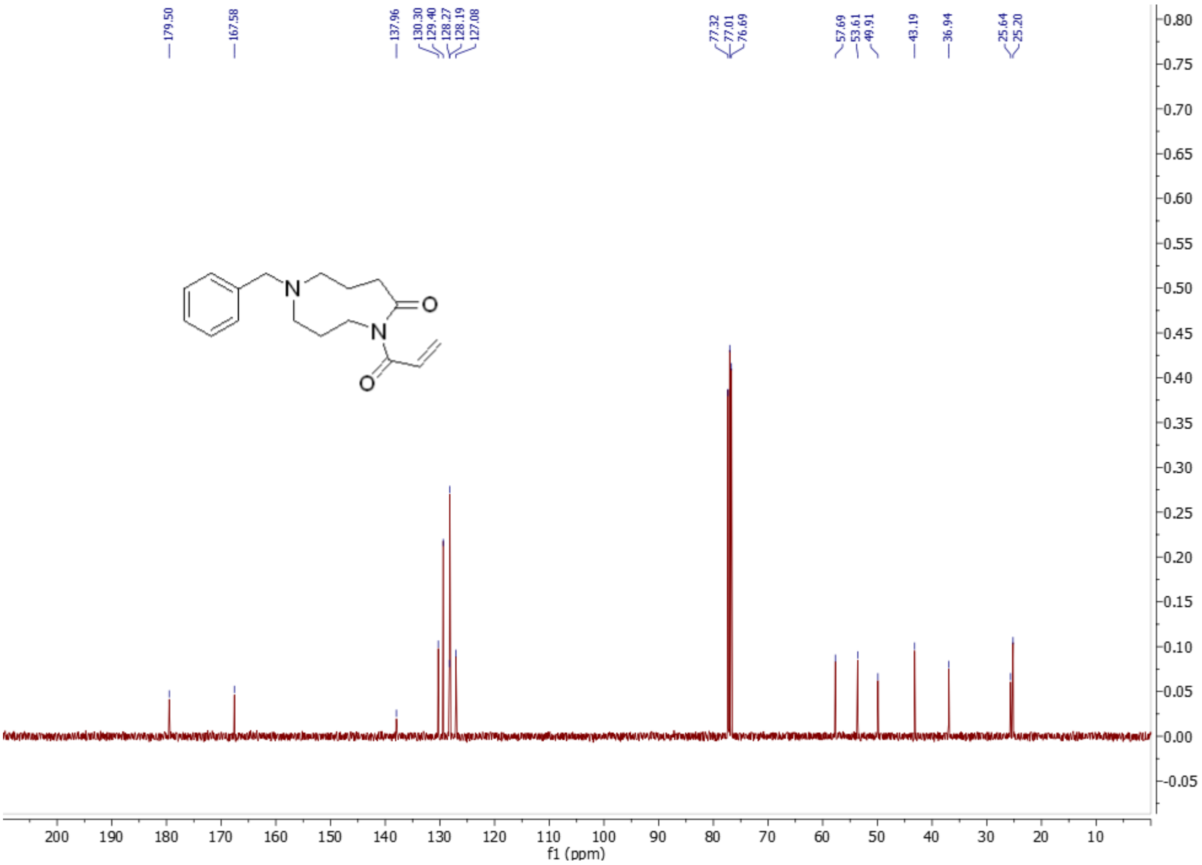

1,9-Dibenzyl-1,5,9-triazacyclotridecane-4,13-dione (**16a**) – in d_6-DMSO; 400 MHz for ^1H, 101 MHz for ^{13}C

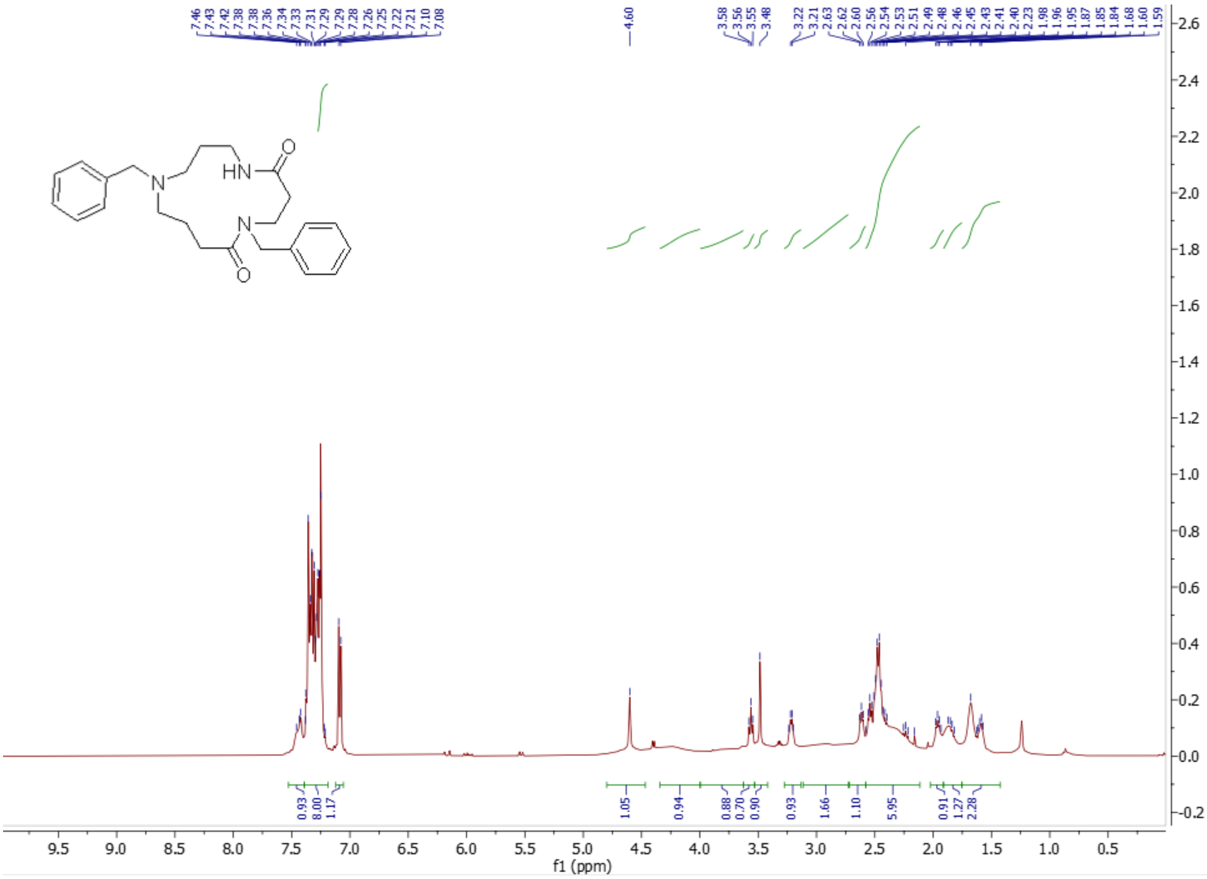

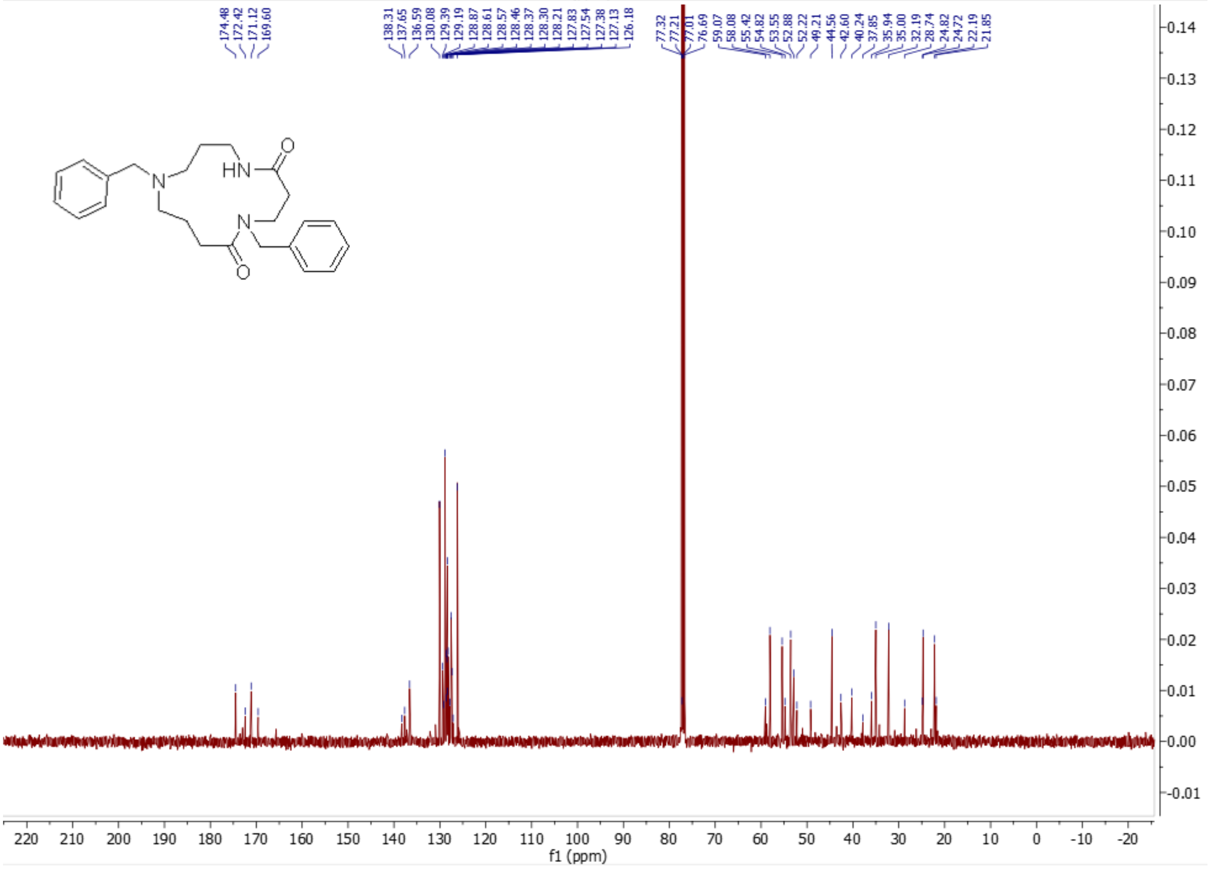

9-Benzyl-1-(4-fluorobenzyl)-1,5,9-triazacyclotridecane-4,13-dione (**16b**) in d_6-DMSO; 700 MHz for ^1H, 176 MHz for ^{13}C

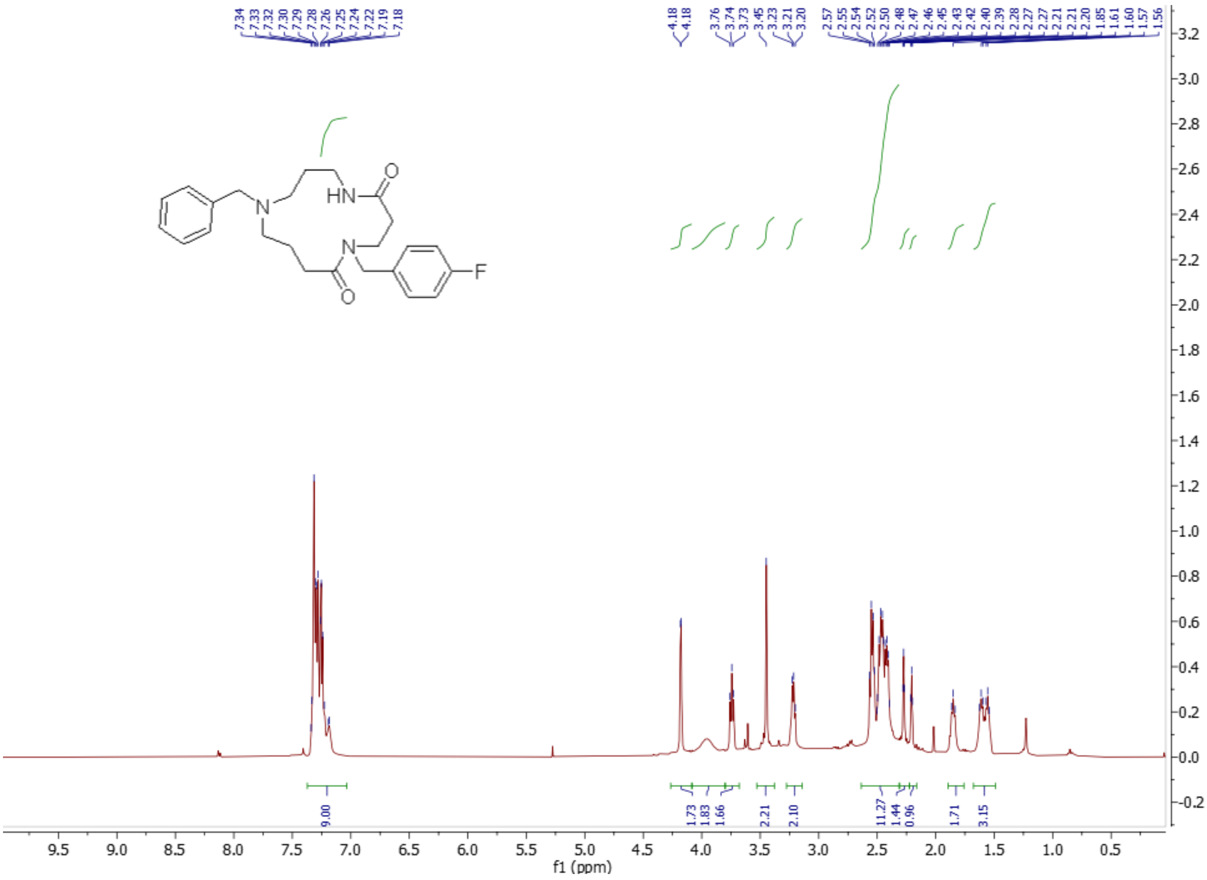

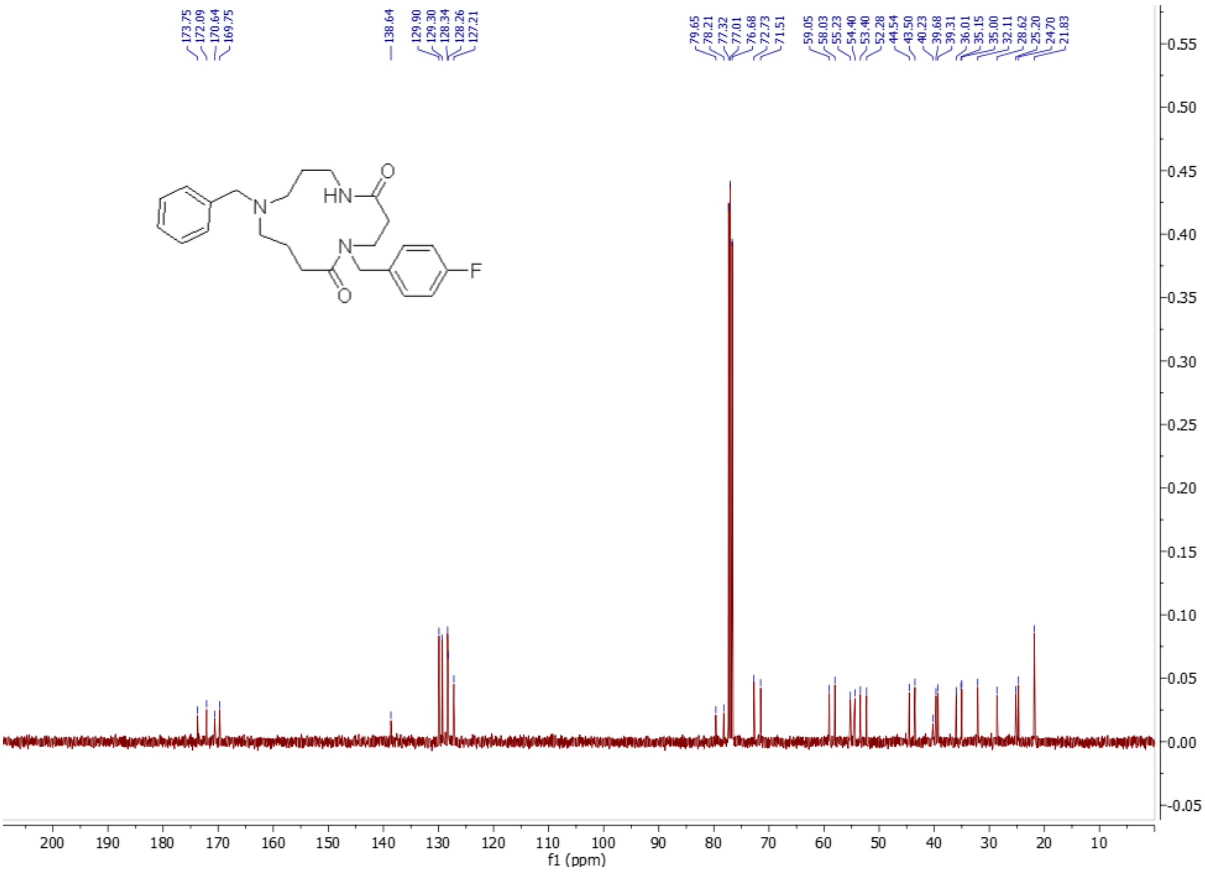

9-Benzyl-1-cyclopropyl-1,5,9-triazacyclotridecane-4,13-dione (**16c**) – in $CDCl_3$; 400 MHz for ^1H, 101 MHz for ^{13}C

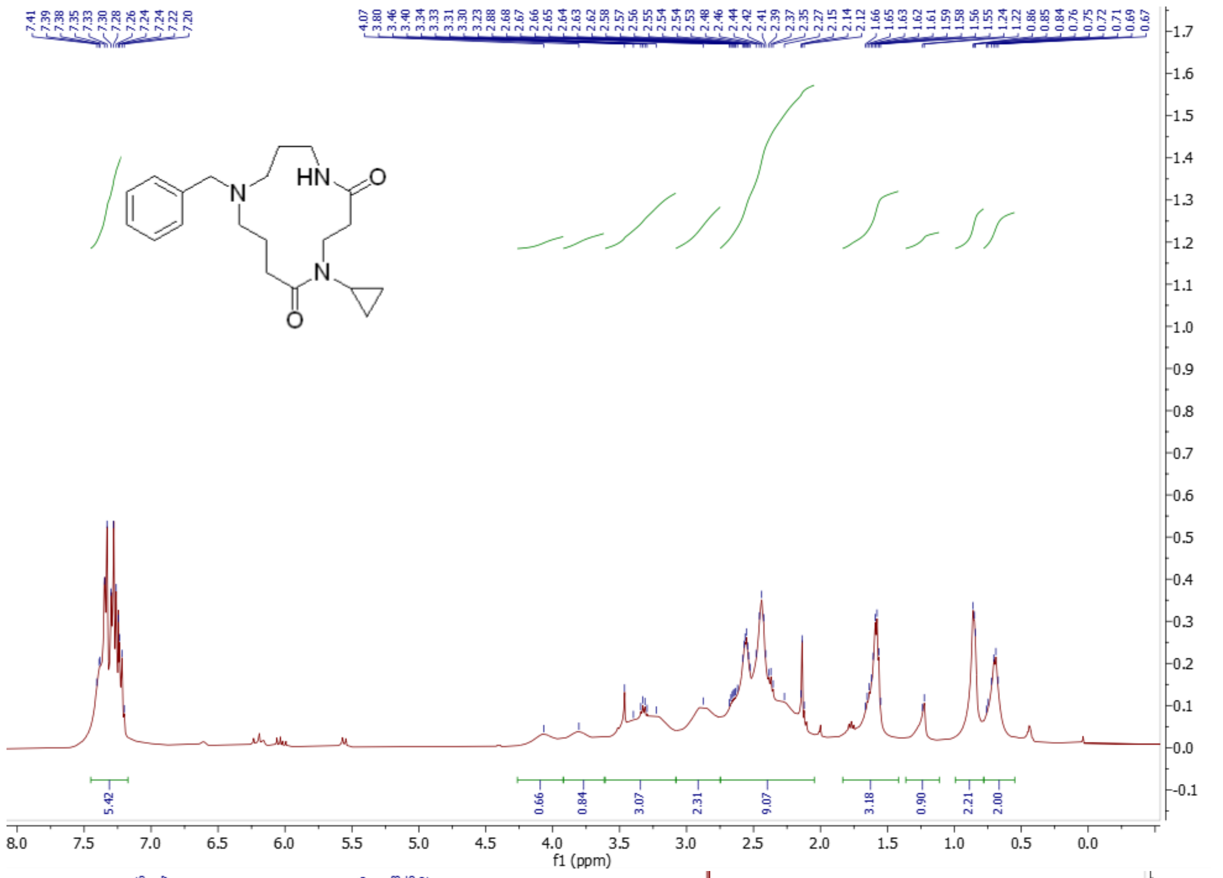

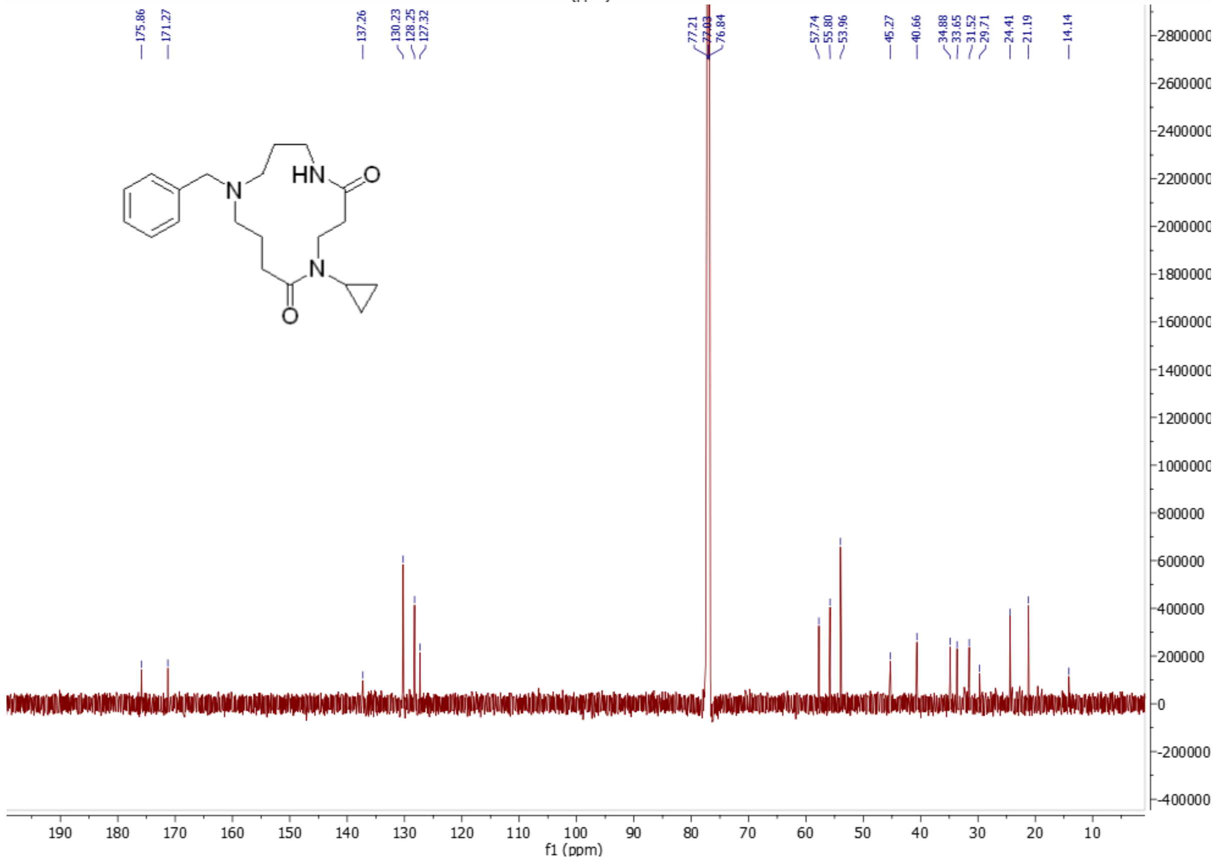

1-Allyl-9-benzyl-1,5,9-triazacyclotridecane-4,13-dione (**16d**) – in $CDCl_3$; 400 MHz for ^{1}H, 101 MHz for ^{13}C

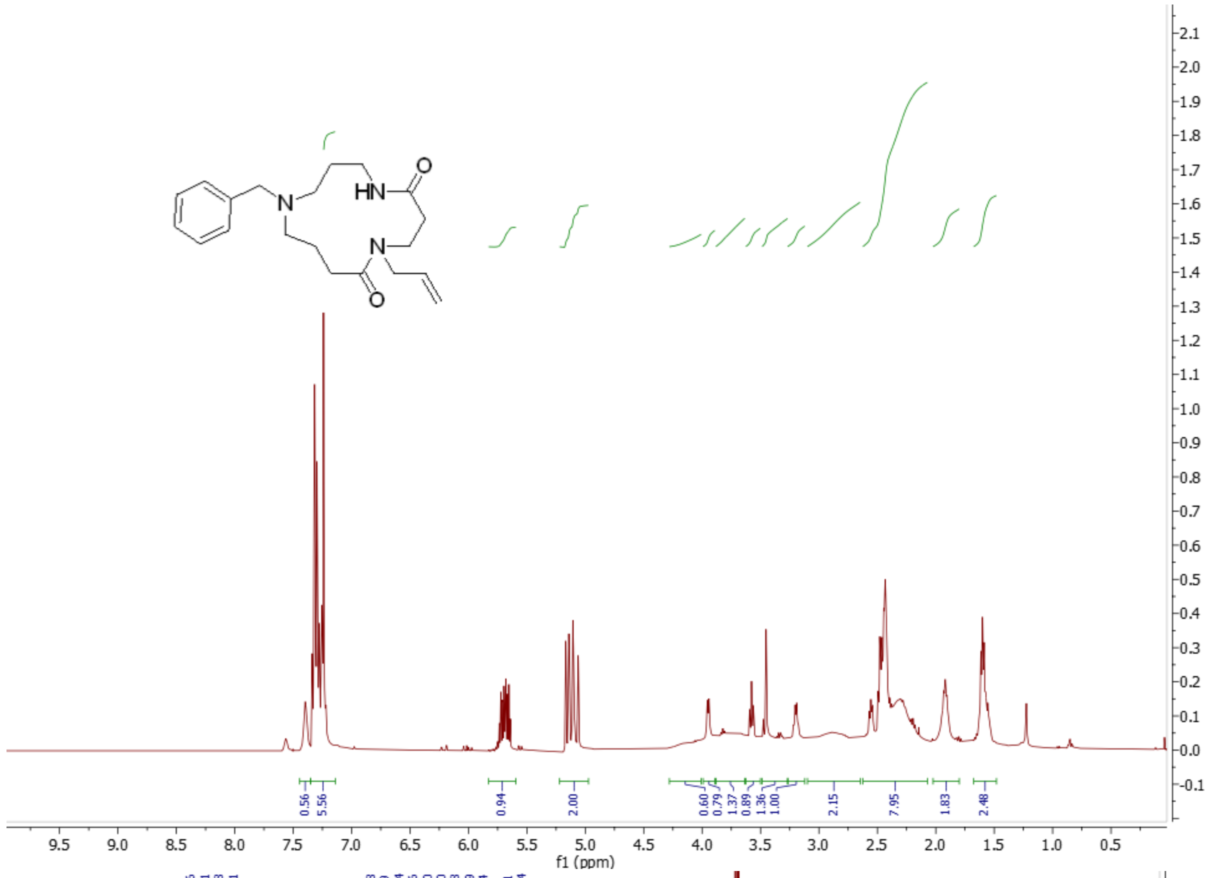

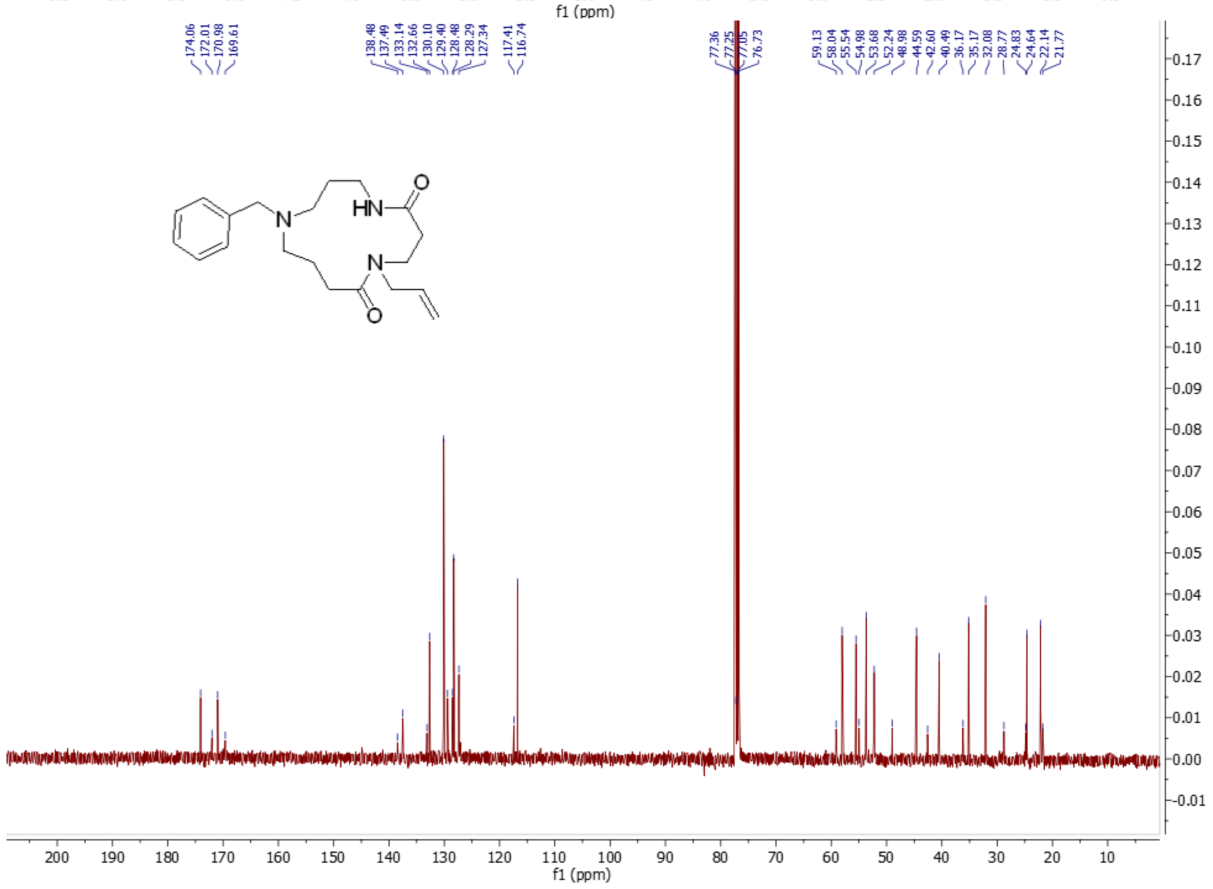

9-Benzyl-1-(prop-2-yn-1-yl)-1,5,9-triazacyclotridecane-4,13-dione (**16e**) in d_6-DMSO; 400 MHz for ^{1}H, 101 MHz for ^{13}C

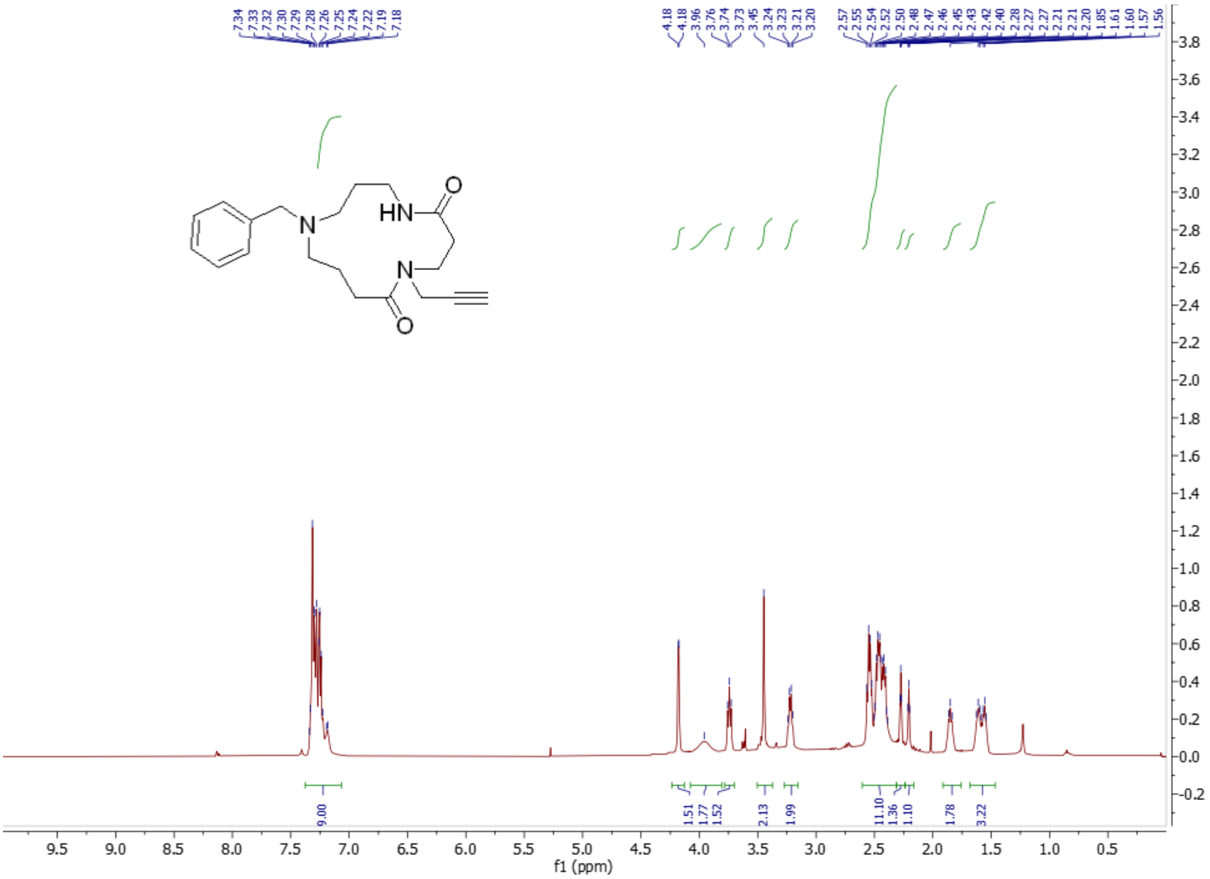

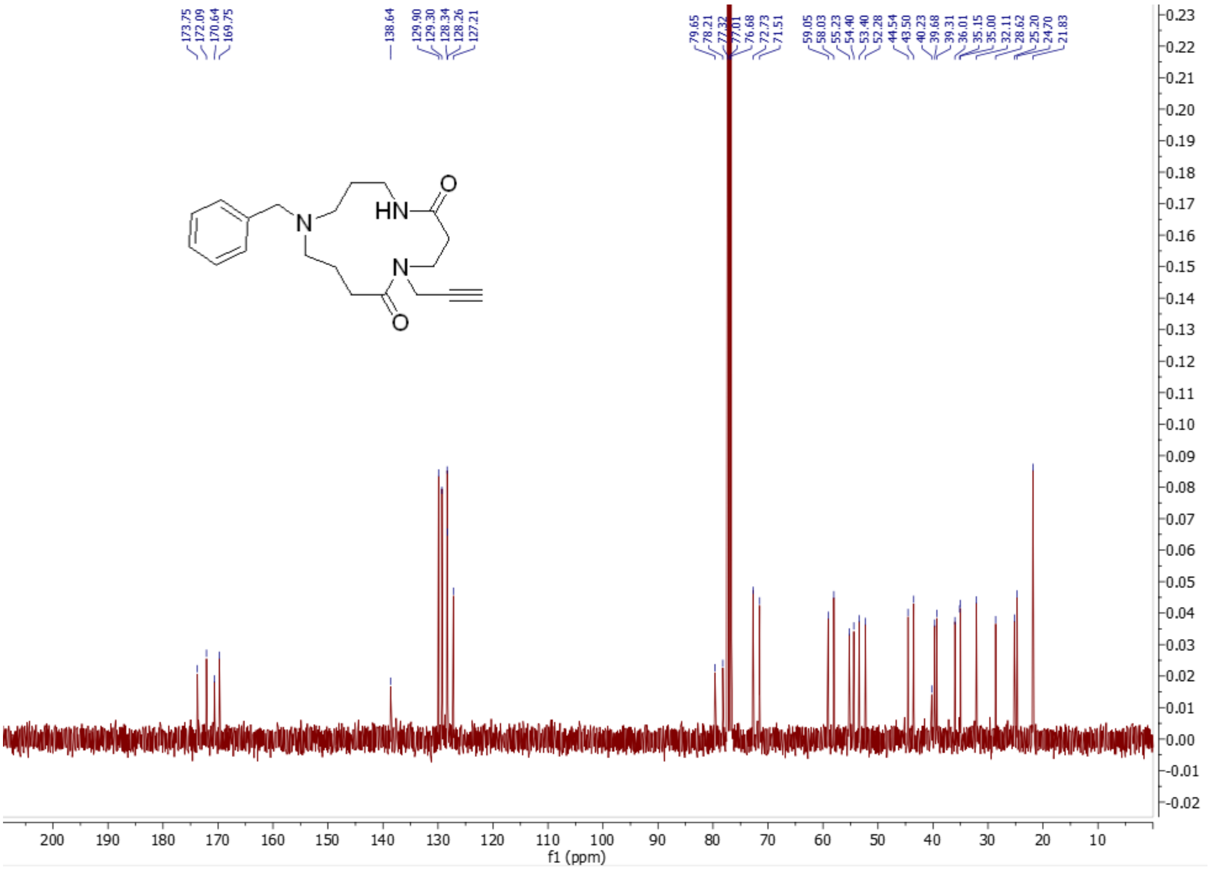

1-(Benzo[d][1,3]dioxol-5-ylmethyl)-9-benzyl-1,5,9-triazacyclotridecane-4,13-dione (**16f**) – in d_6-DMSO; 700 MHz for ^{1}H, 176 MHz for ^{13}C

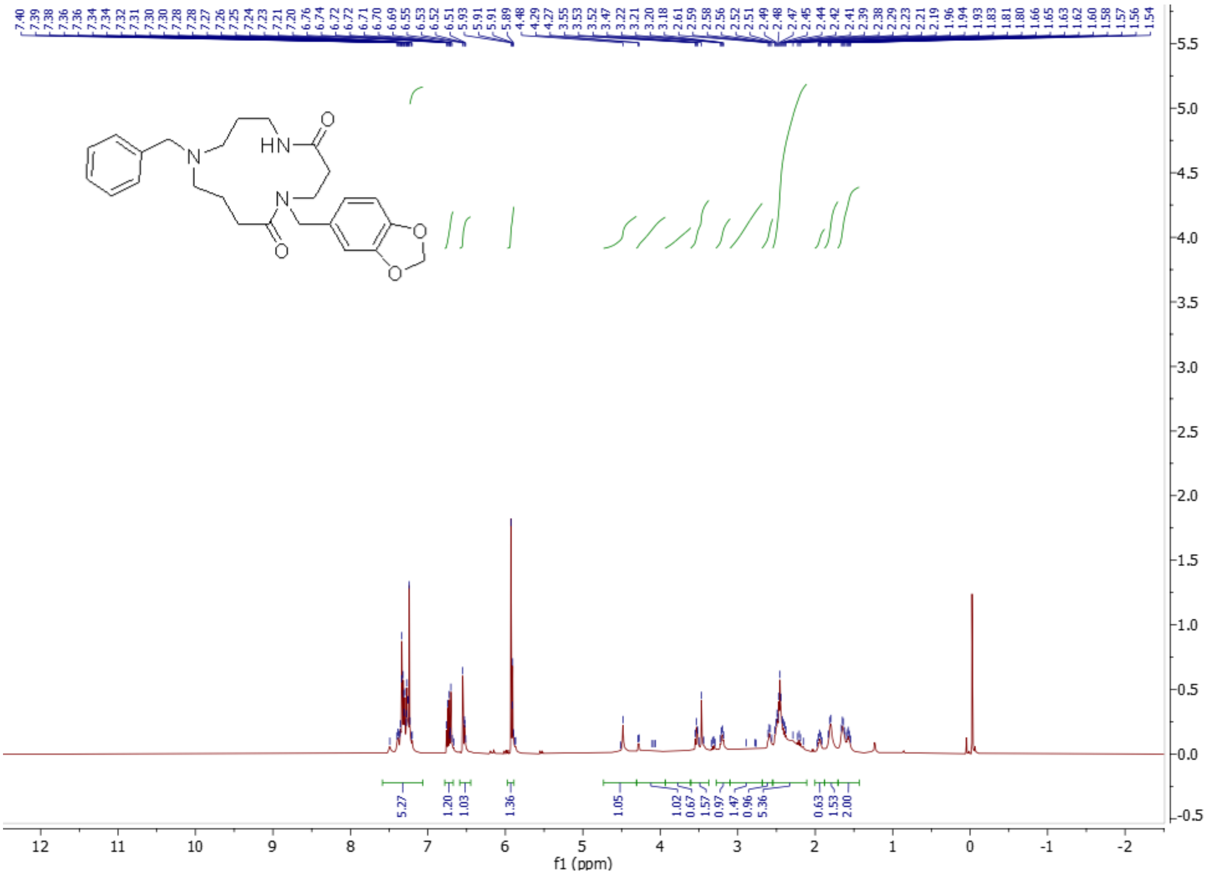

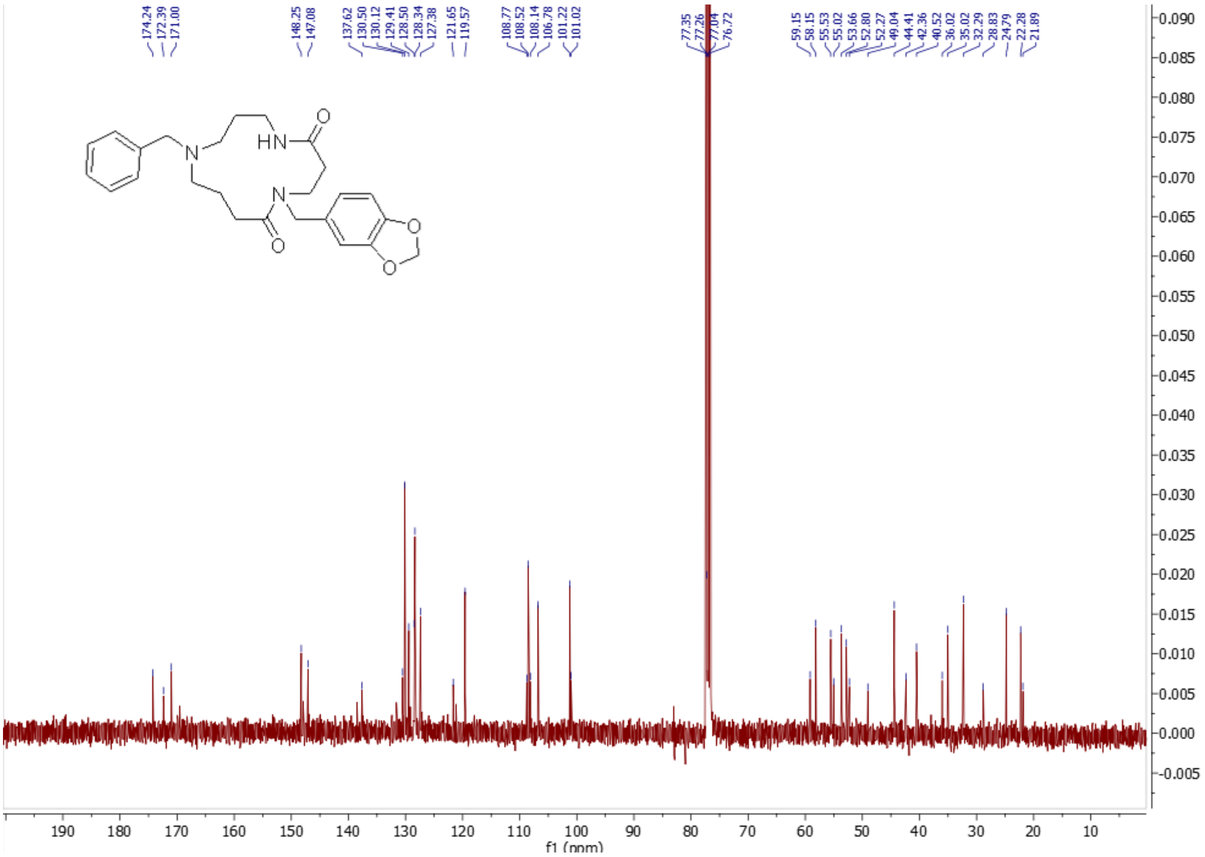

9-Benzyl-1-(2-(methylthio)ethyl)-1,5,9-triazacyclotridecane-4,13-dione (**16g**) in d_6-DMSO; 700 MHz for ^1H, 176 MHz for ^{13}C

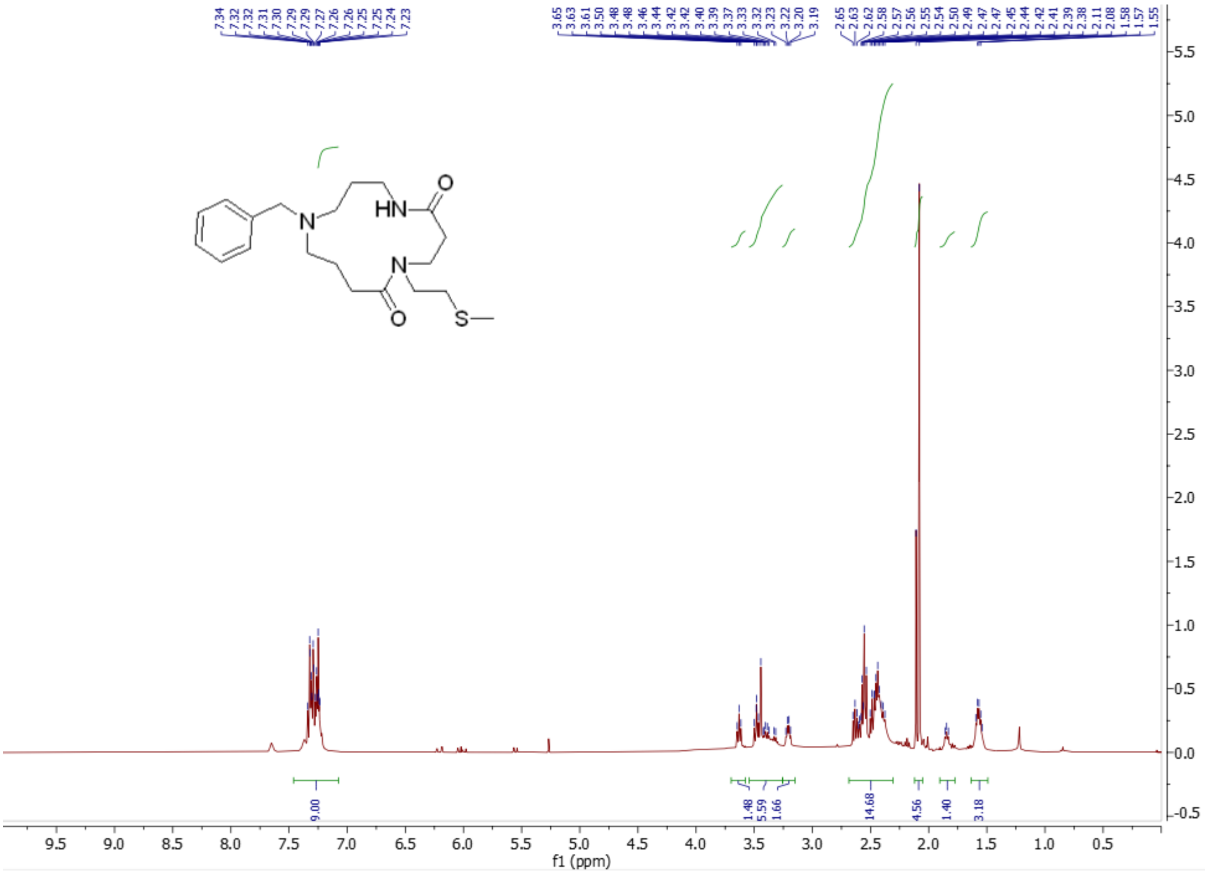

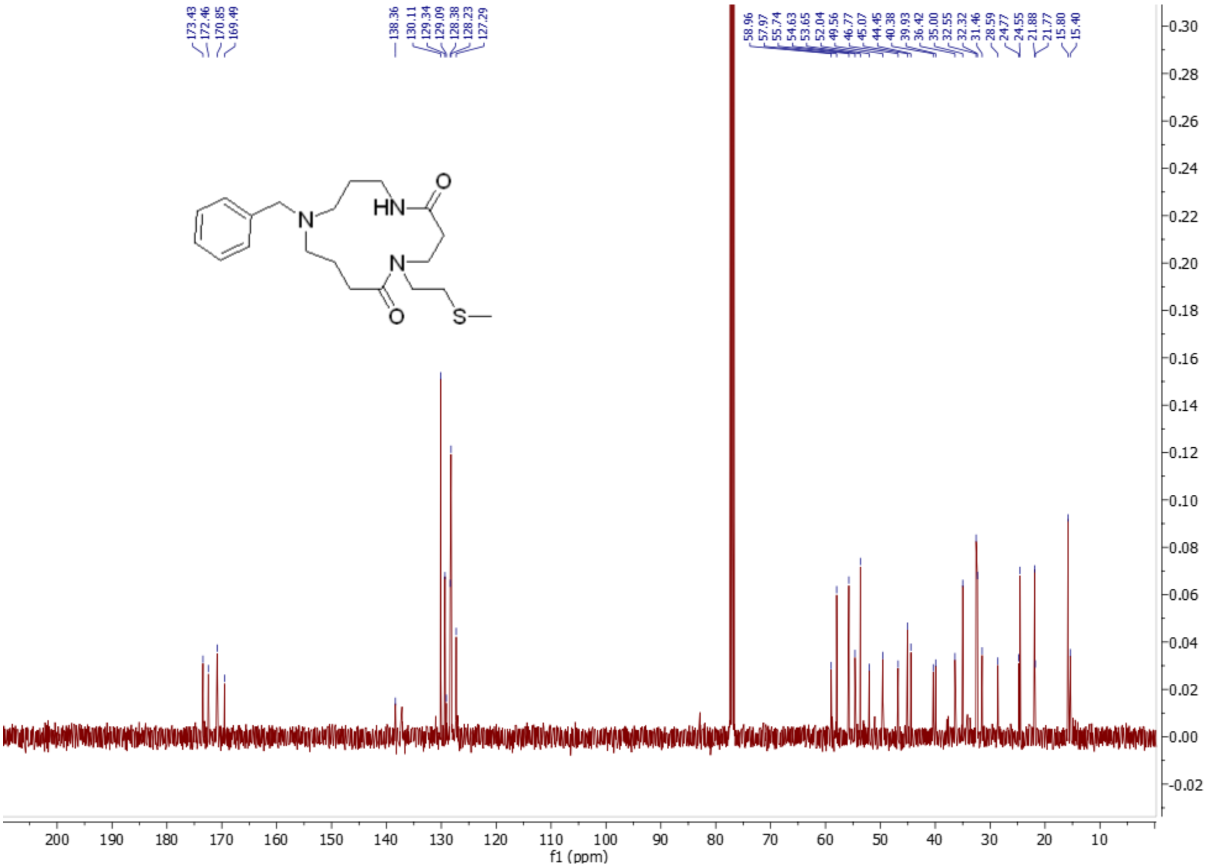

3-(((Benzyloxy)carbonyl)amino)-3-phenylpropanoic acid (**S1**) – in $CDCl_3$; 400 MHz for ^1H, 101 MHz for ^{13}C

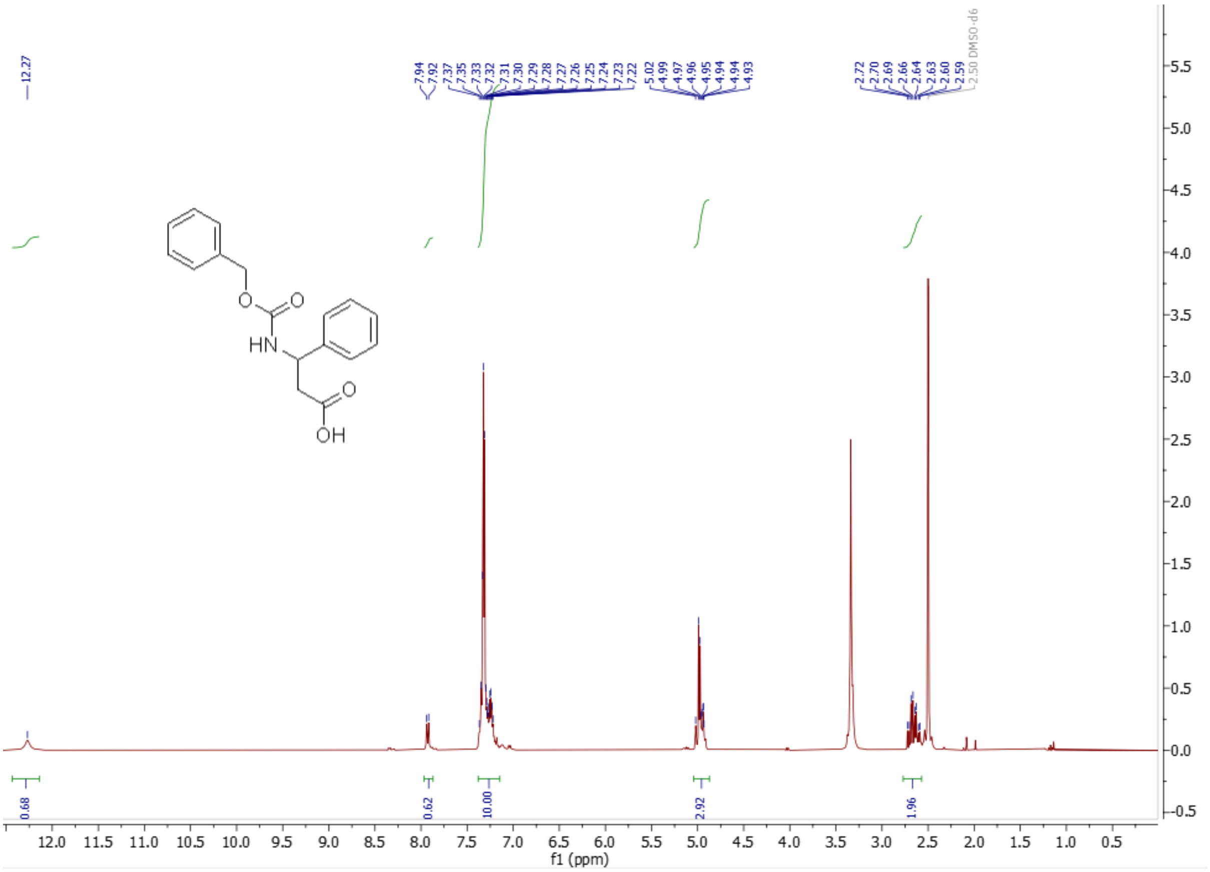

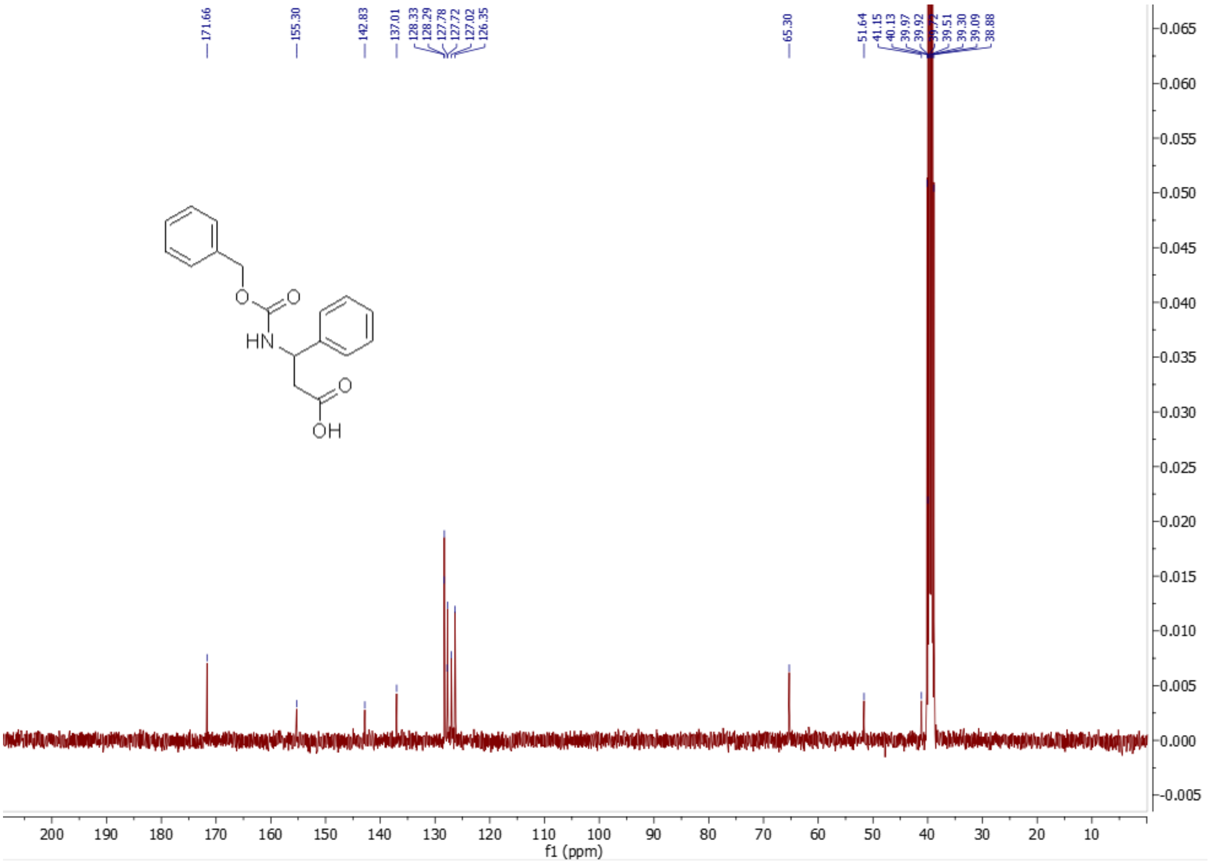

3-((((9H-Fluoren-9-yl)methoxy)carbonyl)amino)-3-phenylpropanoic acid (**S2**) – in $CDCl_3$; 400 MHz for ^{1}H, 101 MHz for ^{13}C

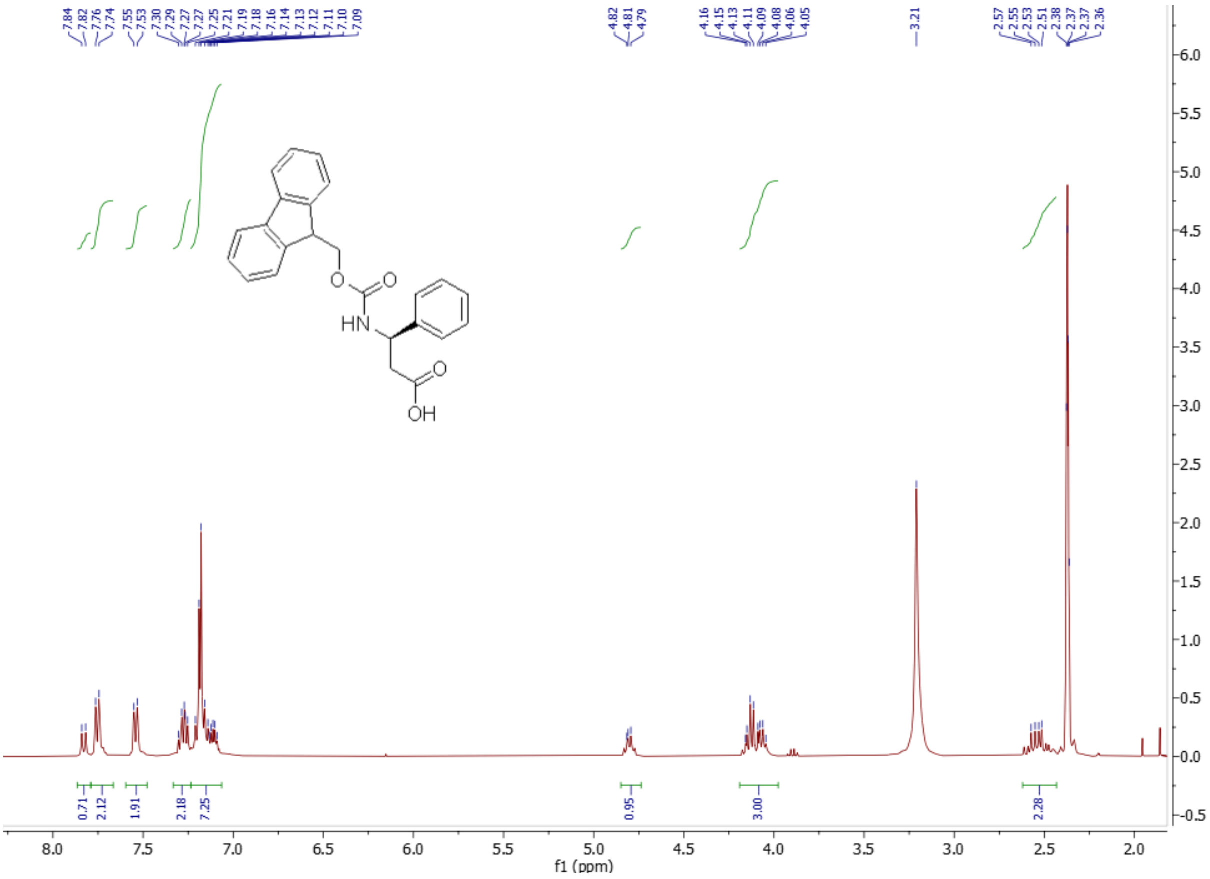

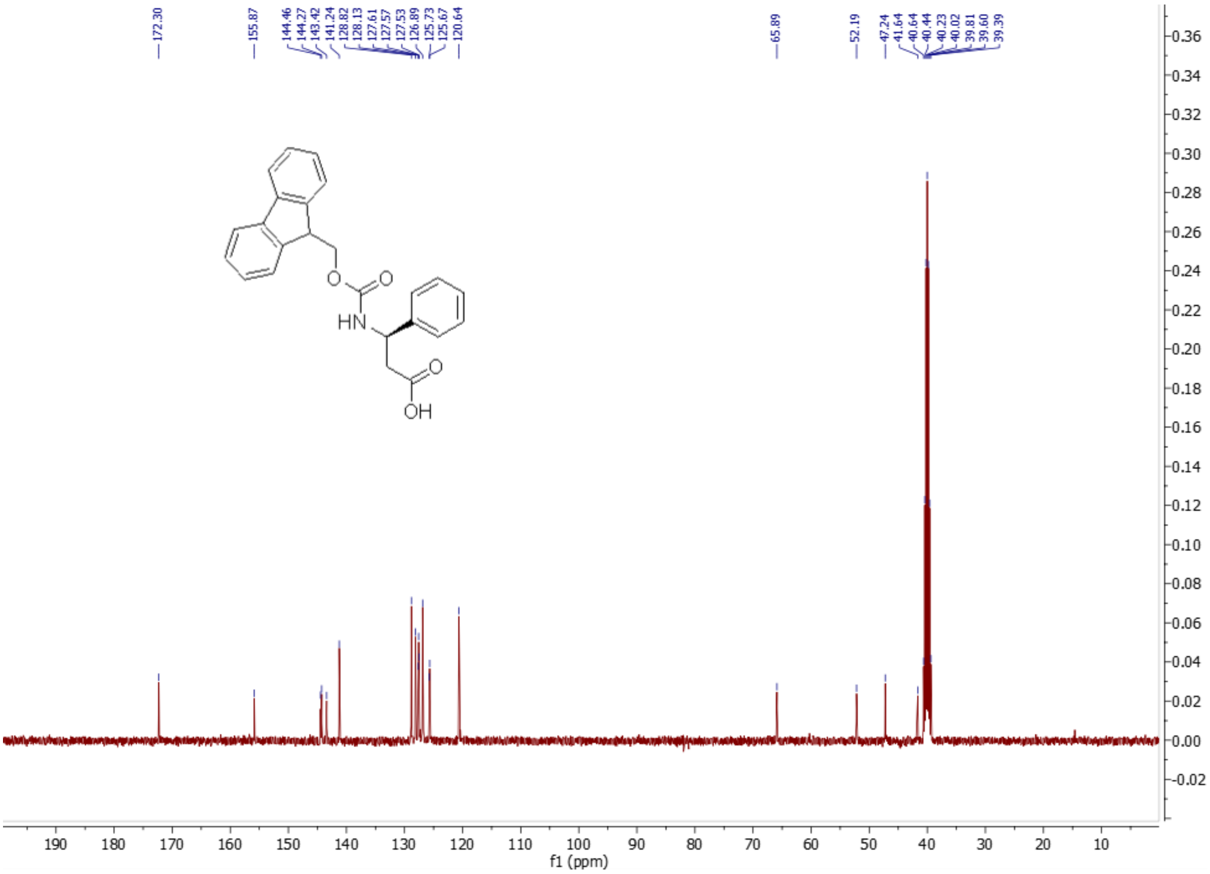

3-((((9H-Fluoren-9-yl)methoxy)carbonyl)amino)butanoic acid (**S3**) – in $CDCl_3$; 400 MHz for ^1H, 101 MHz for ^{13}C

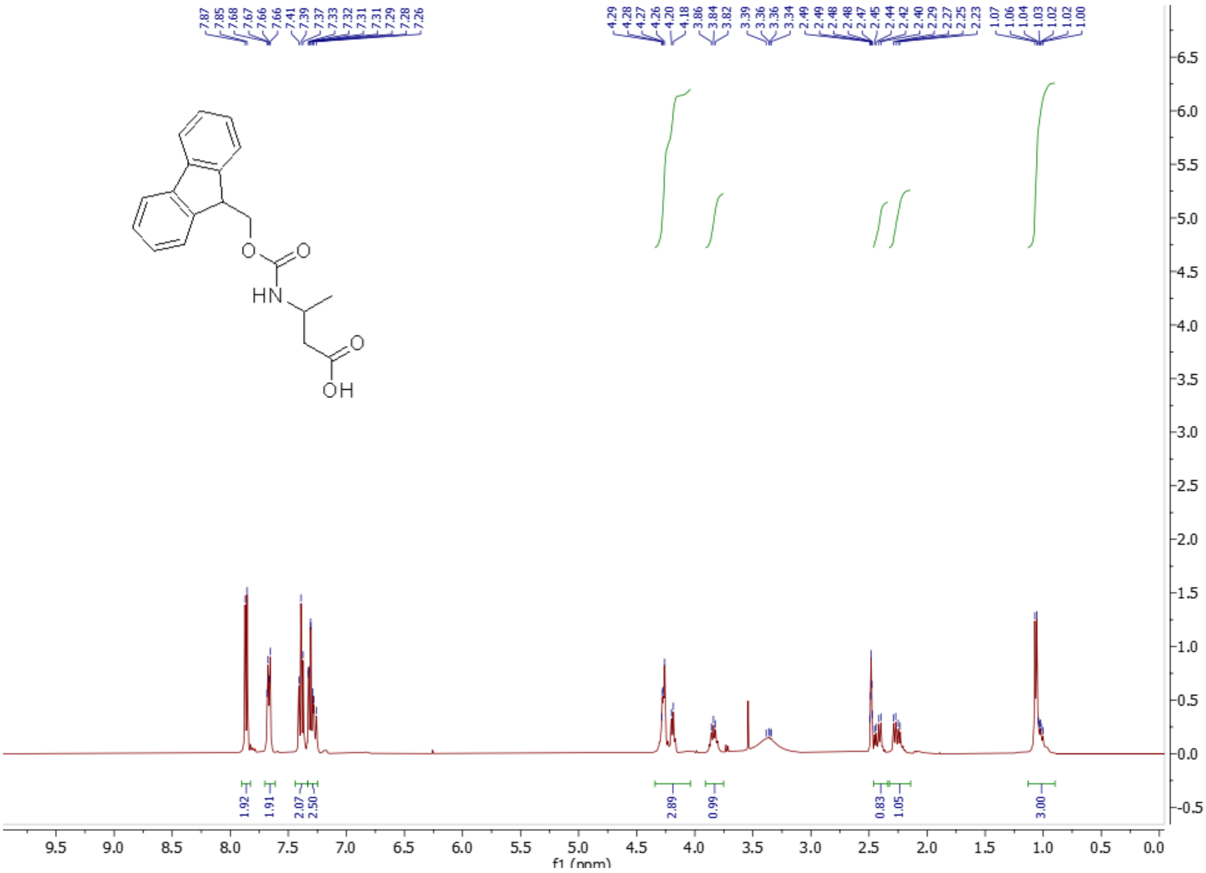

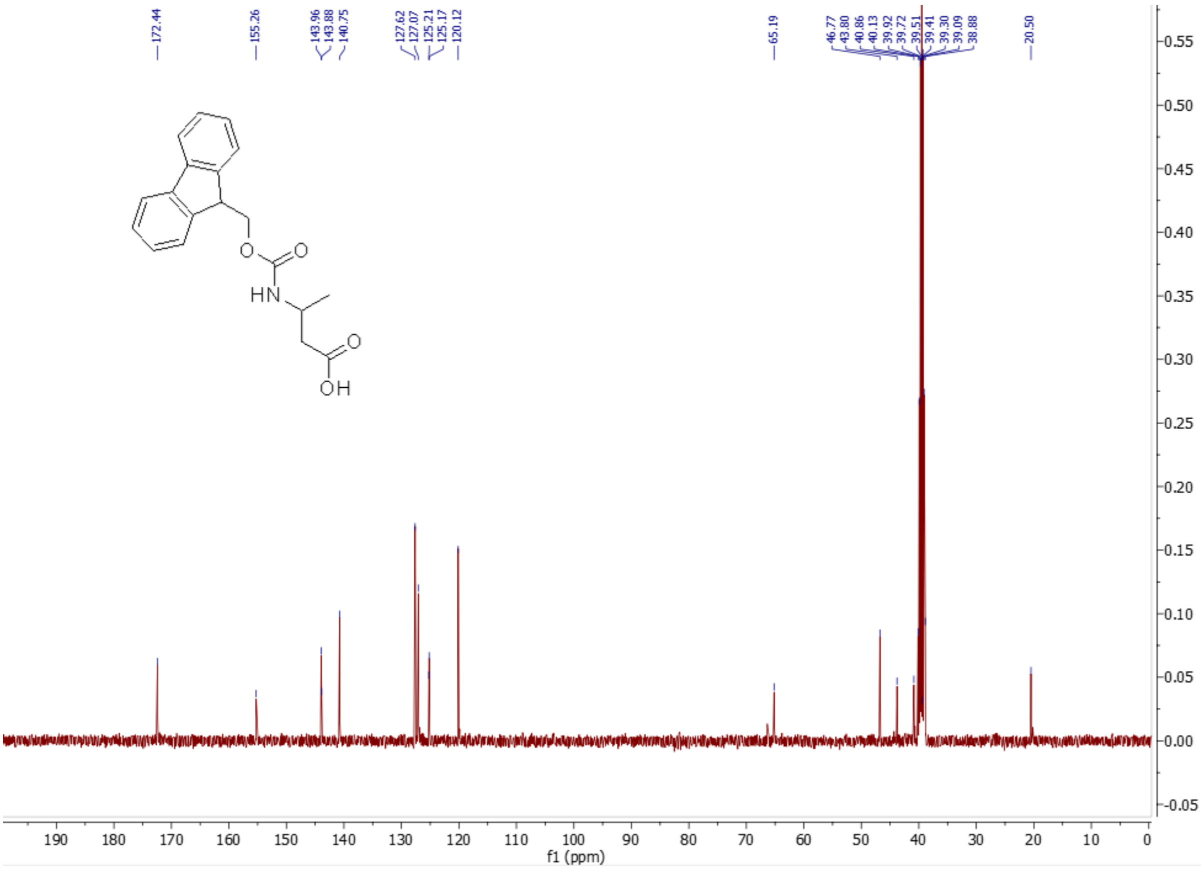

3-((((9H-Fluoren-9-yl)methoxy)carbonyl)(methyl)amino)propanoic acid (**S4**) – in $CDCl_3$; 400 MHz for ^{1}H, 101 MHz for ^{13}C

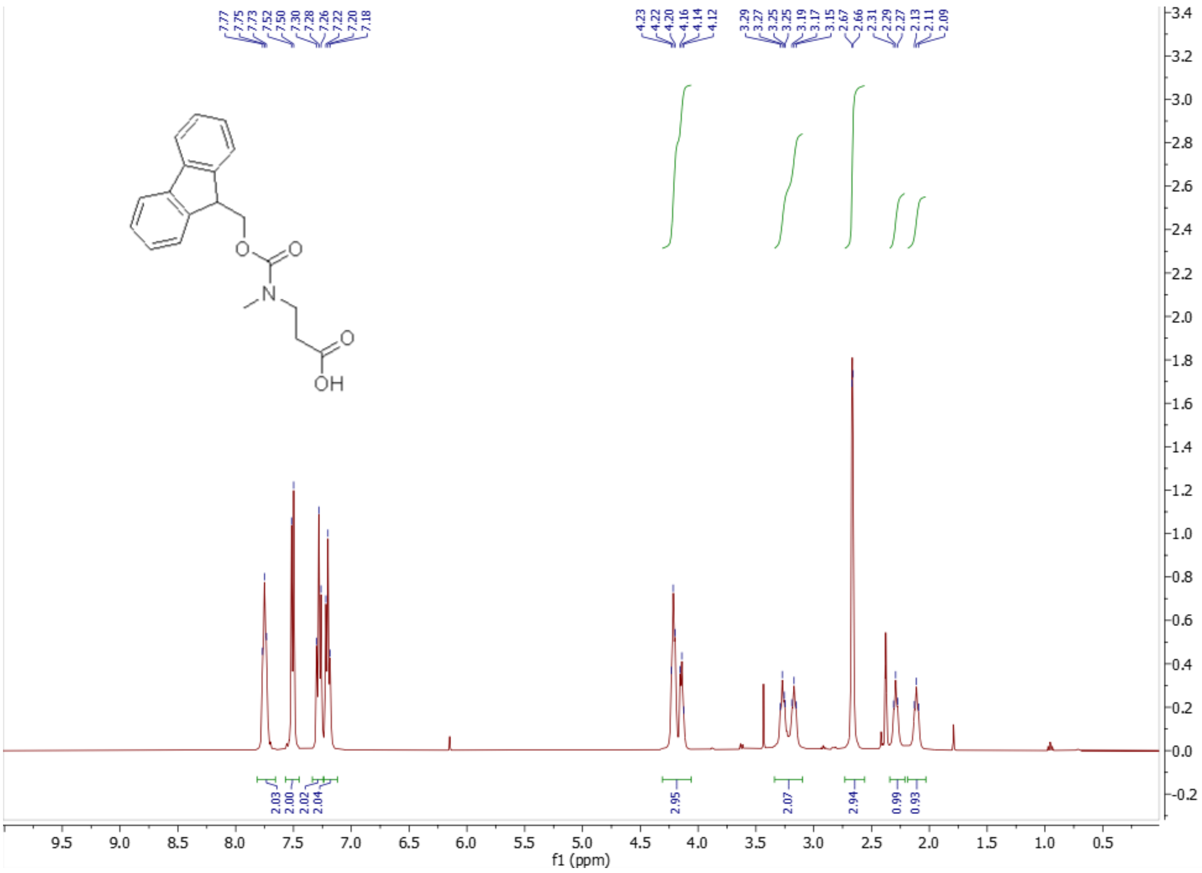

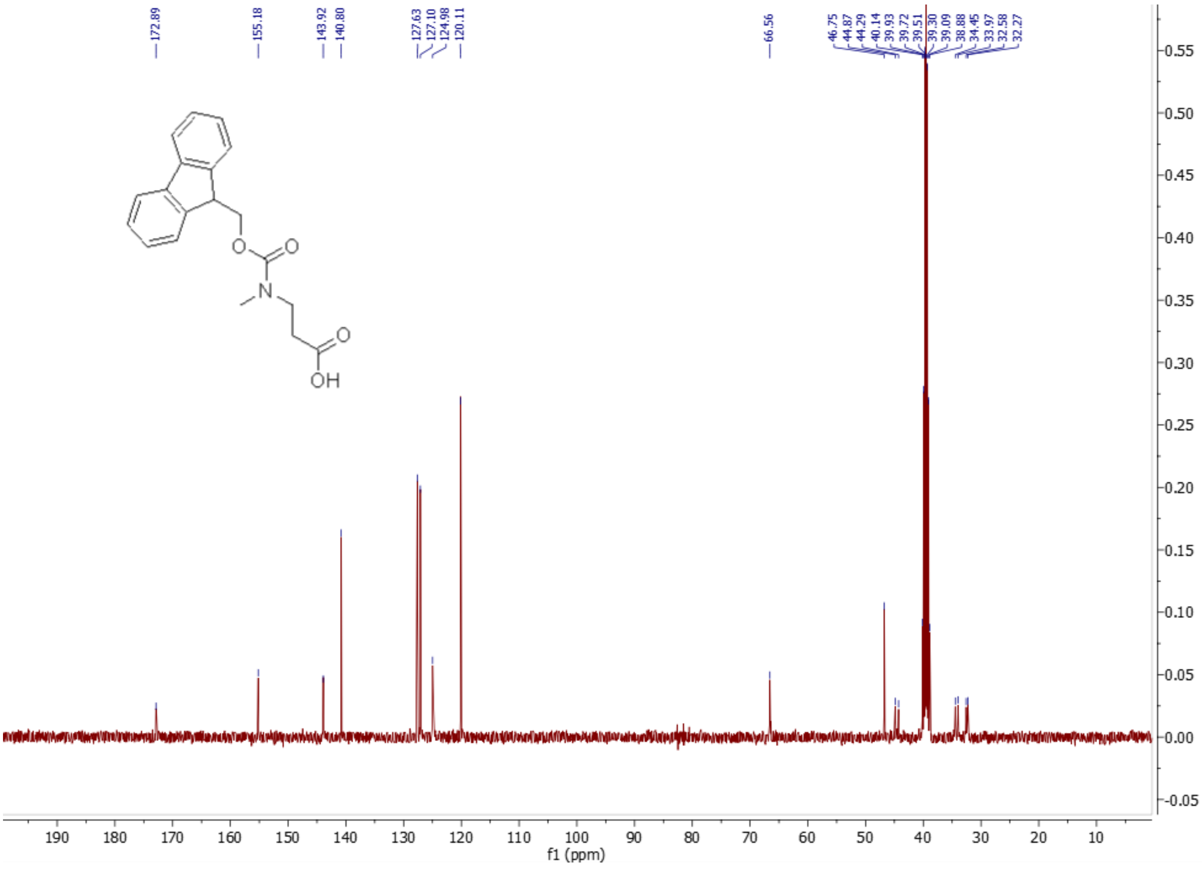

Benzyl (3-(5-benzyl-9-oxo-1,5-diazonan-1-yl)-3-oxo-1-phenylpropyl)carbamate (**18a**) – in $CDCl_3$; 400 MHz for ^{1}H, 101 MHz for ^{13}C

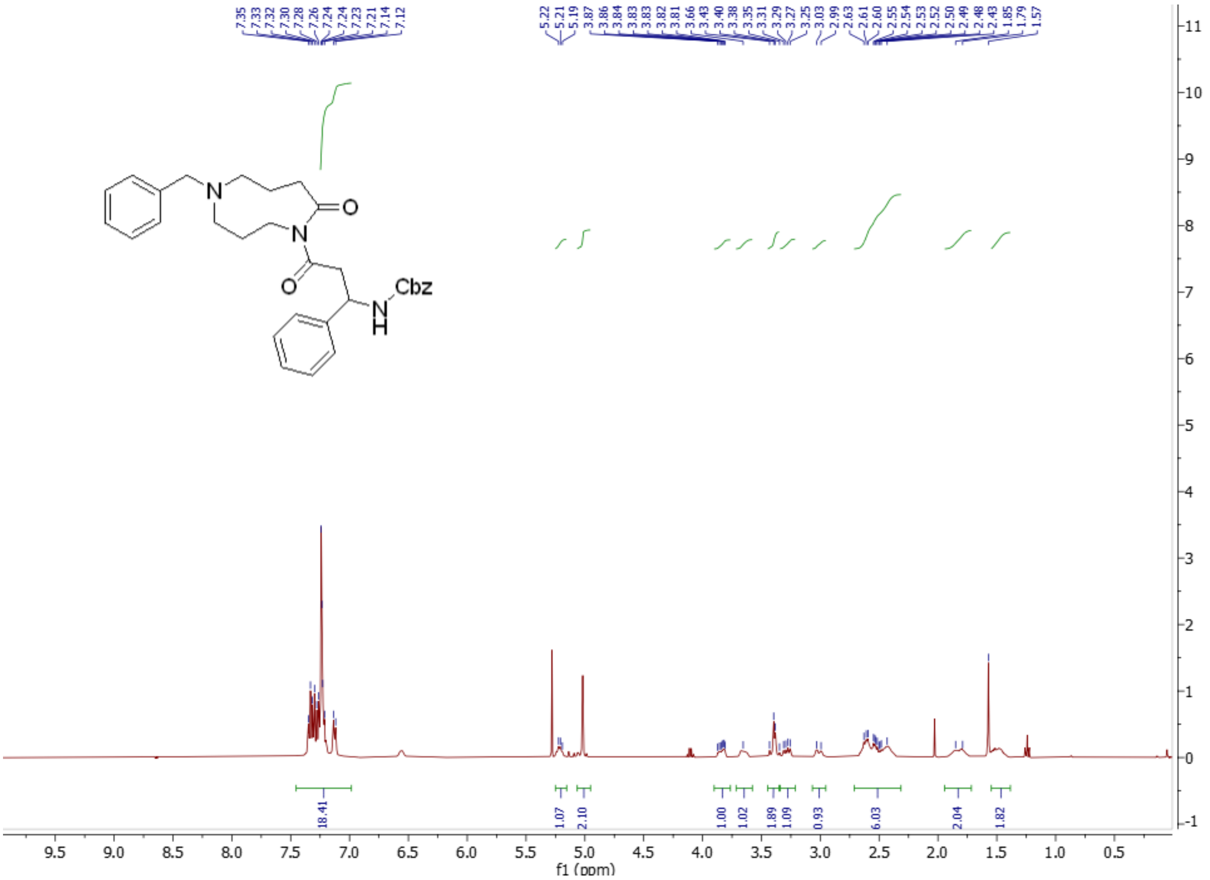

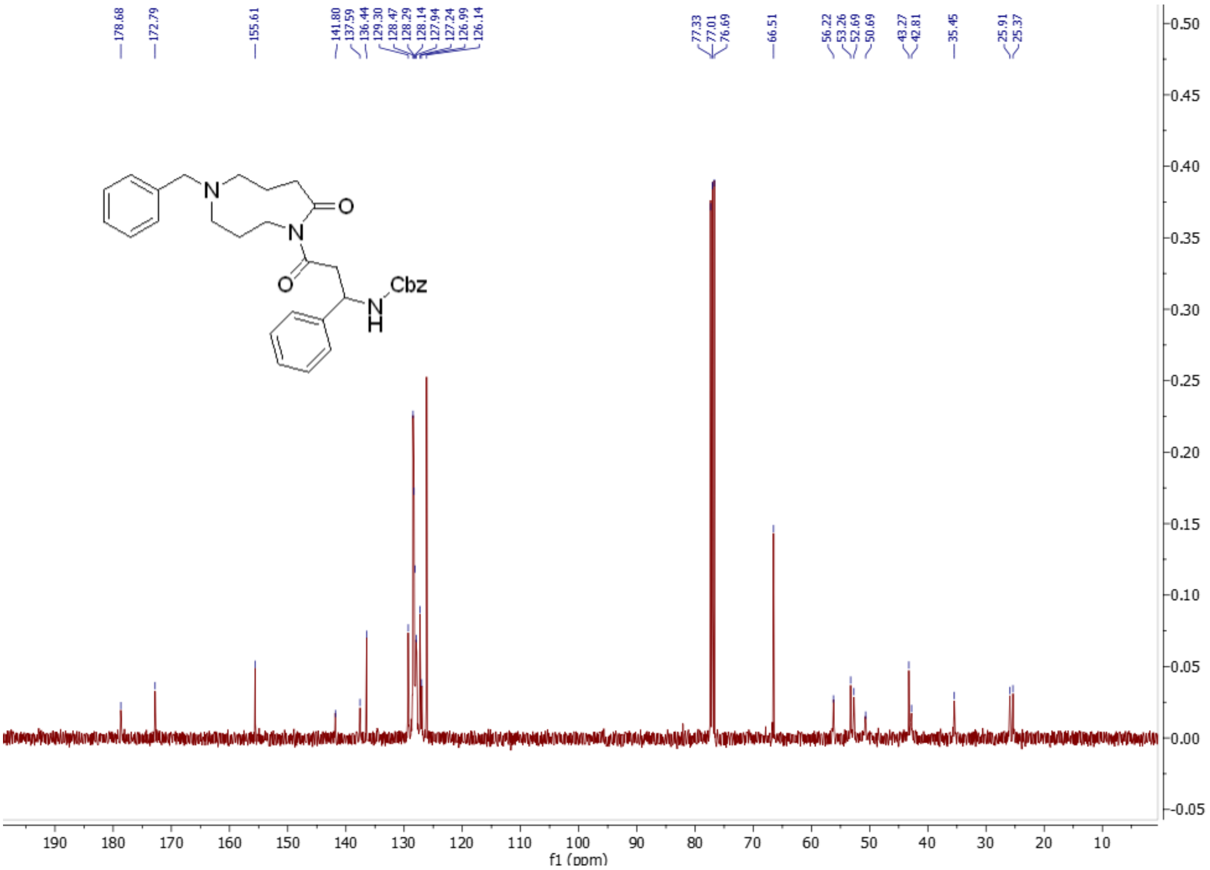

9-Benzyl-2-phenyl-1,5,9-triazacyclotridecane-4,13-dione (**19a**) in d_6-DMSO; 400 MHz for ^{1}H, 101 MHz for ^{13}C

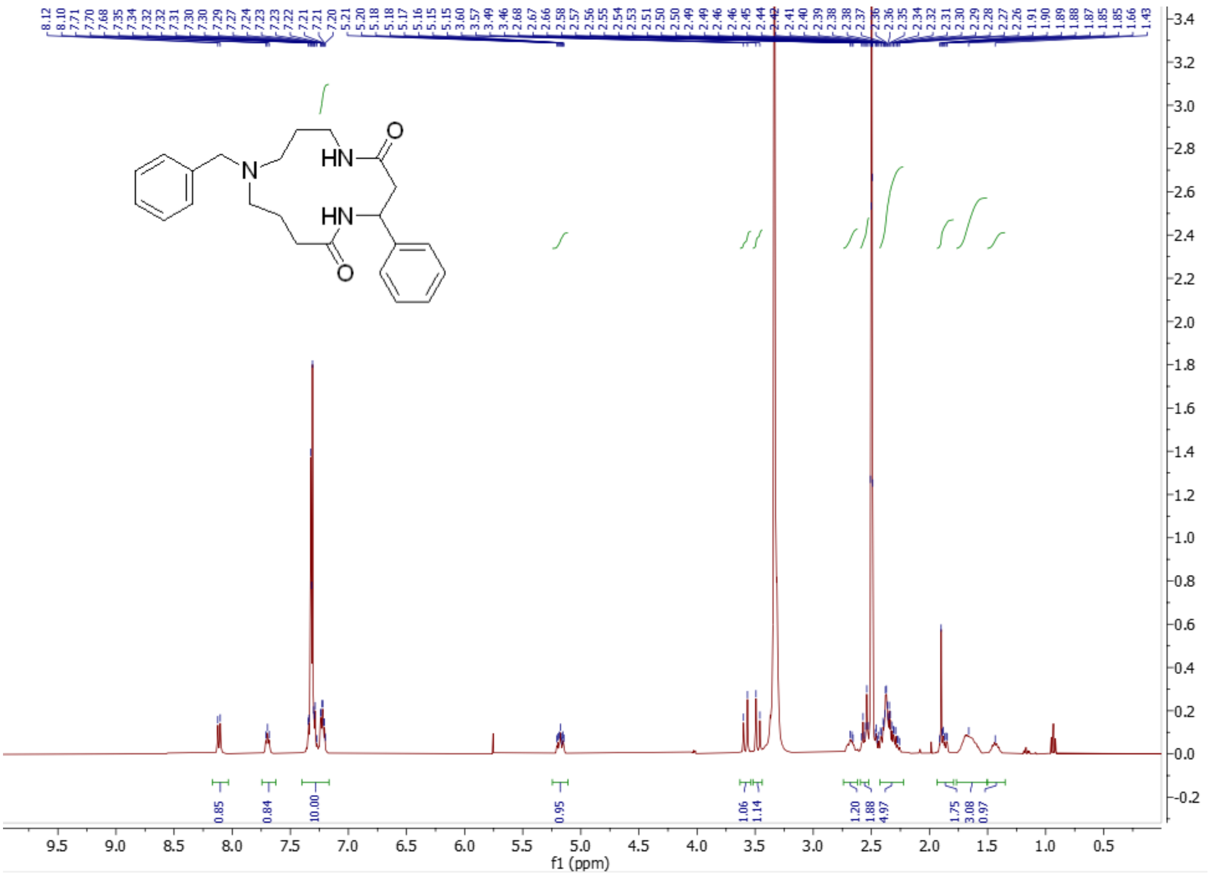

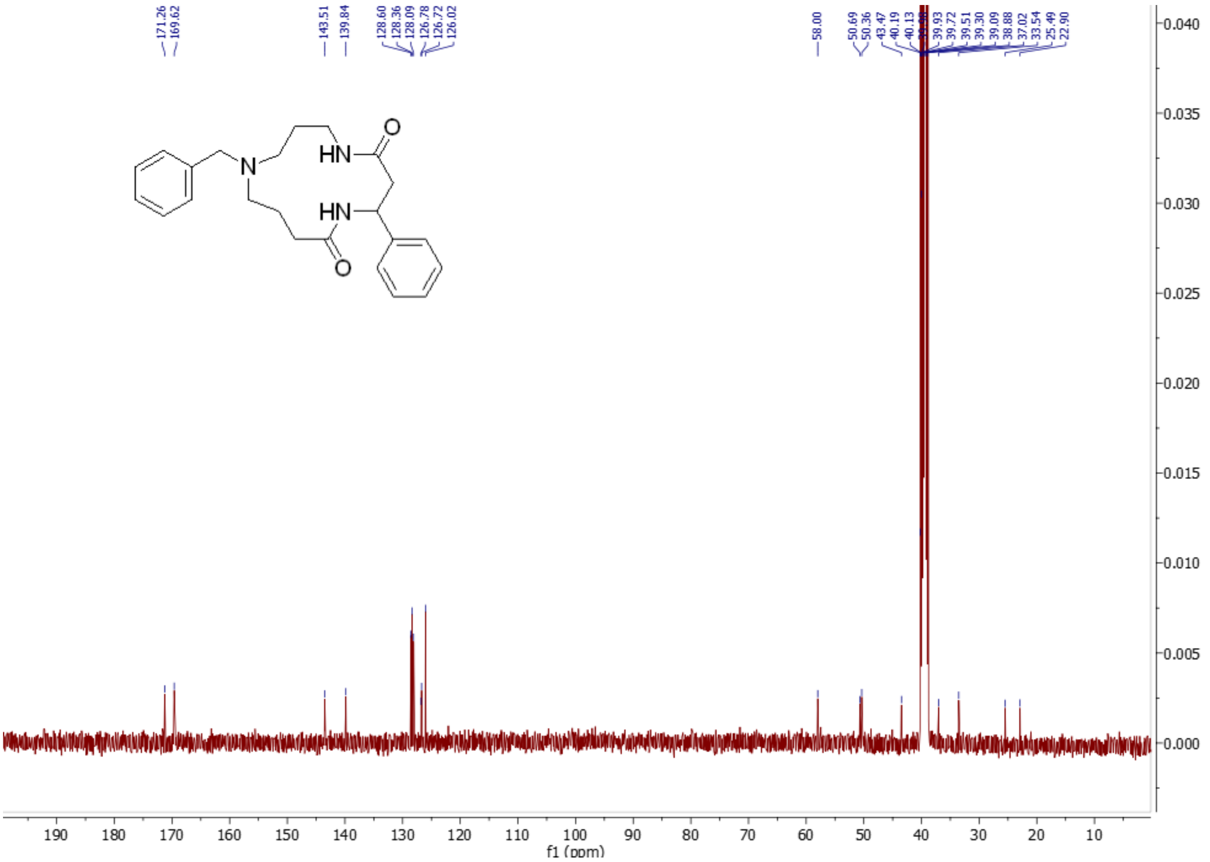

(9H-Fluoren-9-yl)methyl (3-(5-(4-methoxyphenyl)-9-oxo-1,5-diazonan-1-yl)-3-oxo-1-phenylpropyl)carbamate (**18b**) – in $CDCl_3$; 400 MHz for ^1H, 101 MHz for ^{13}C

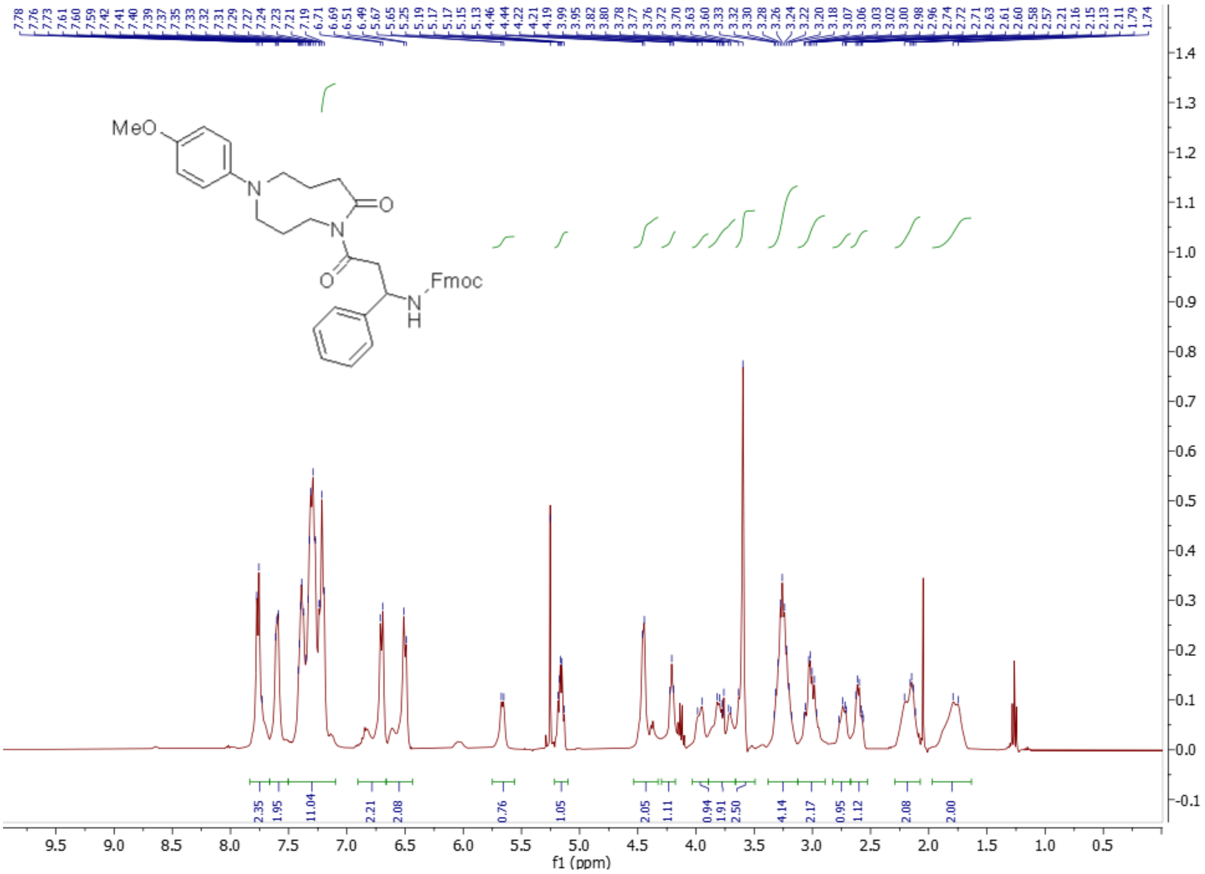

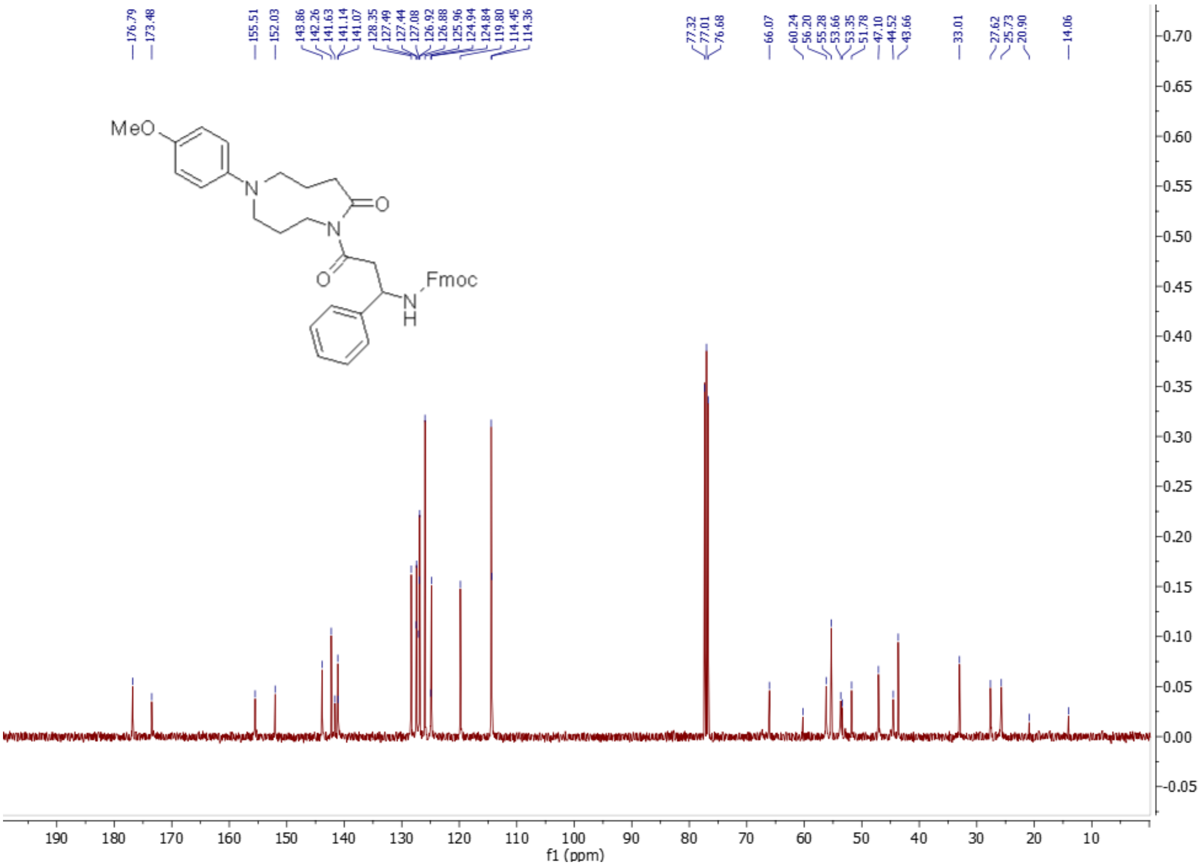

(*R*)-9-(4-Methoxyphenyl)-2-phenyl-1,5,9-triazacyclotridecane-4,13-dione (**19b**) in d_6-DMSO; 400 MHz for ^1H, 101 MHz for ^{13}C

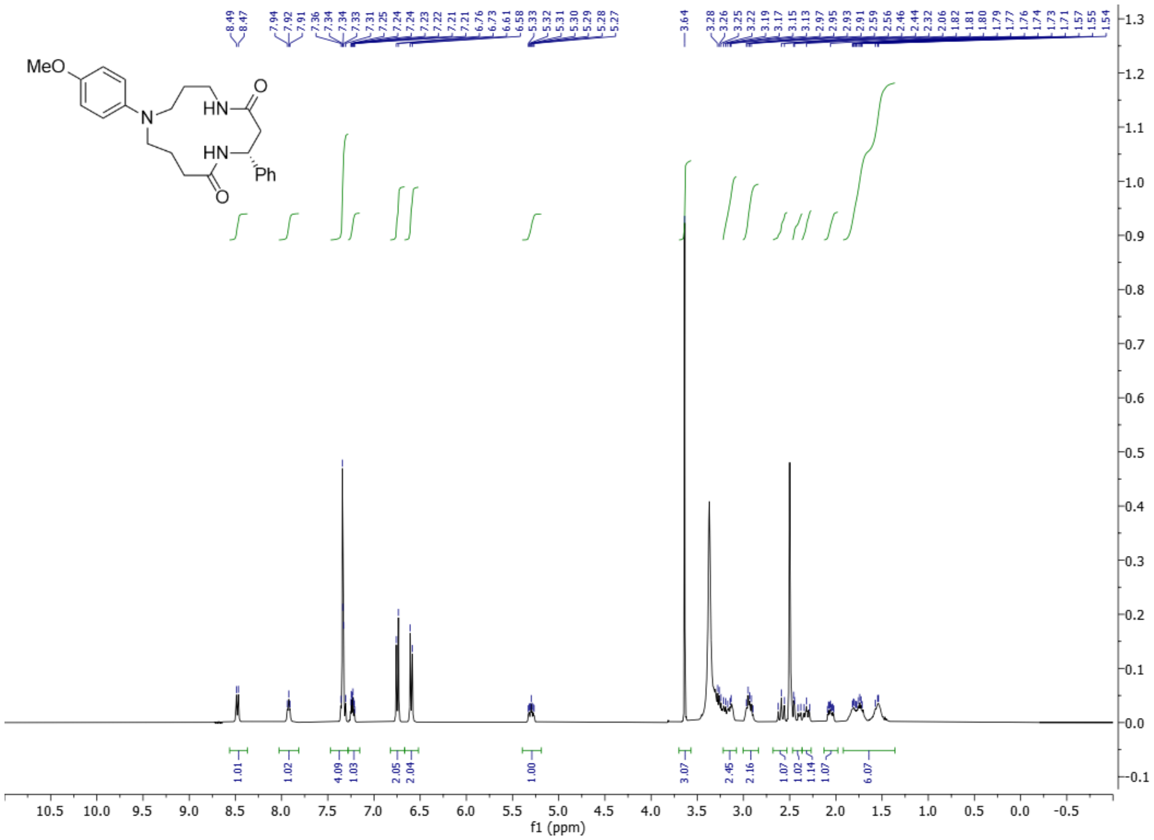

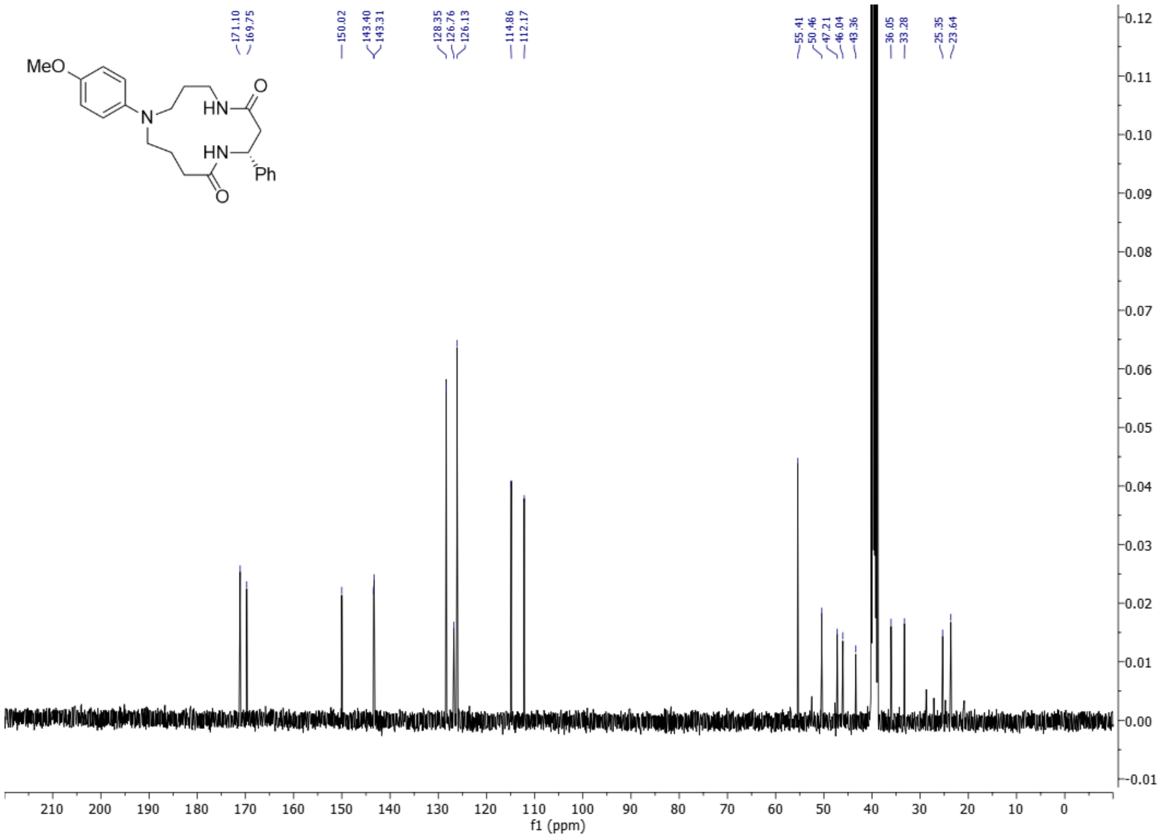

9-(4-Methoxyphenyl)-2-methyl-1,5,9-triazacyclotridecane-4,13-dione (**19c**) – in $CDCl_3$; 400 MHz for ^1H, 101 MHz for ^{13}C

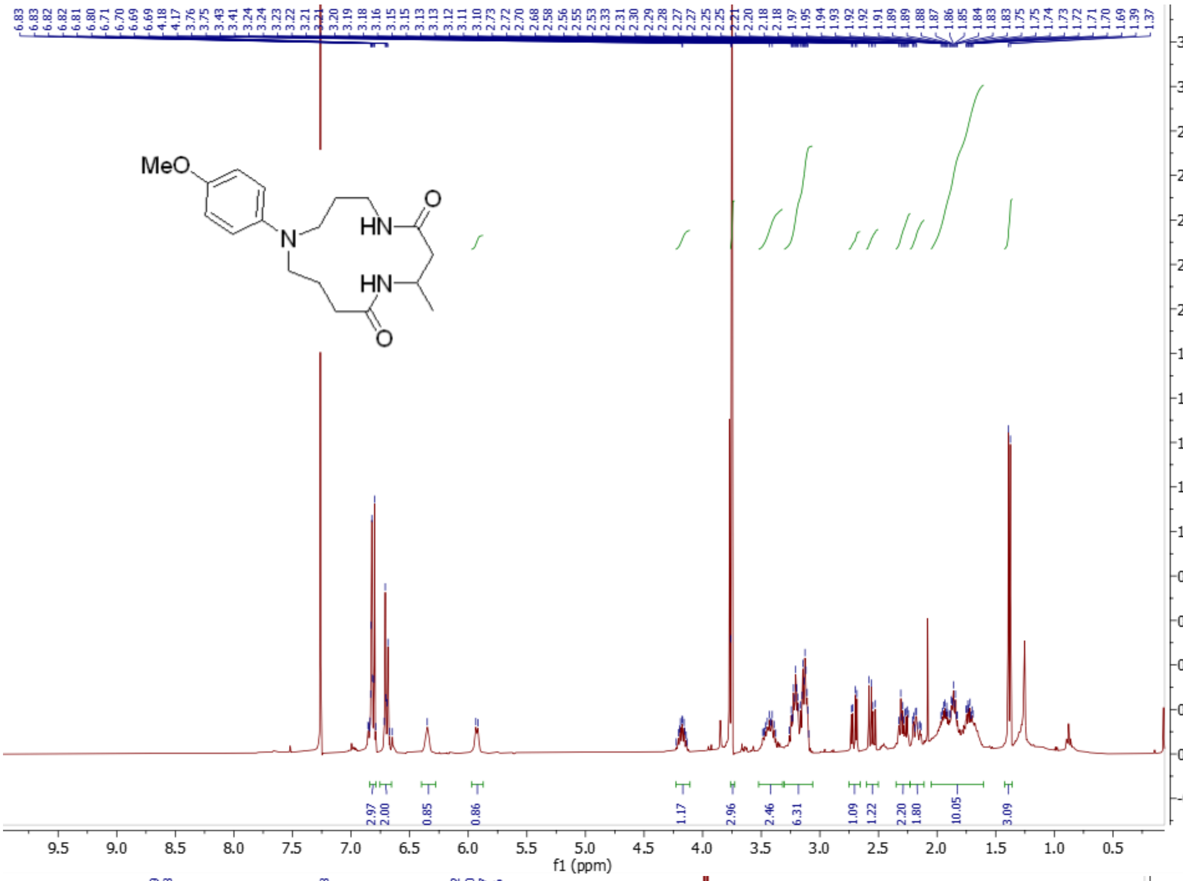

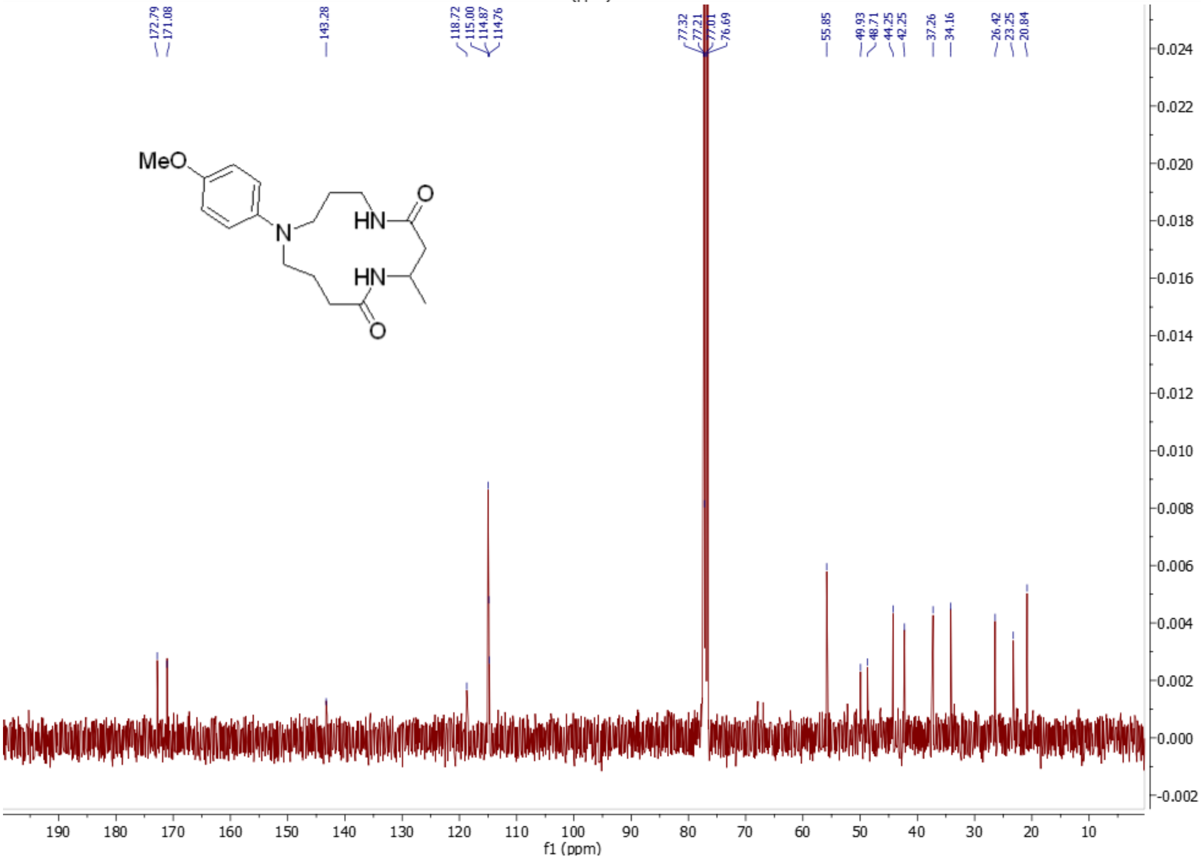

9-(4-Methoxyphenyl)-1-methyl-1,5,9-triazacyclotridecane-4,13-dione (**19d**) – in $CDCl_3$; 400 MHz for ^1H, 101 MHz for ^{13}C

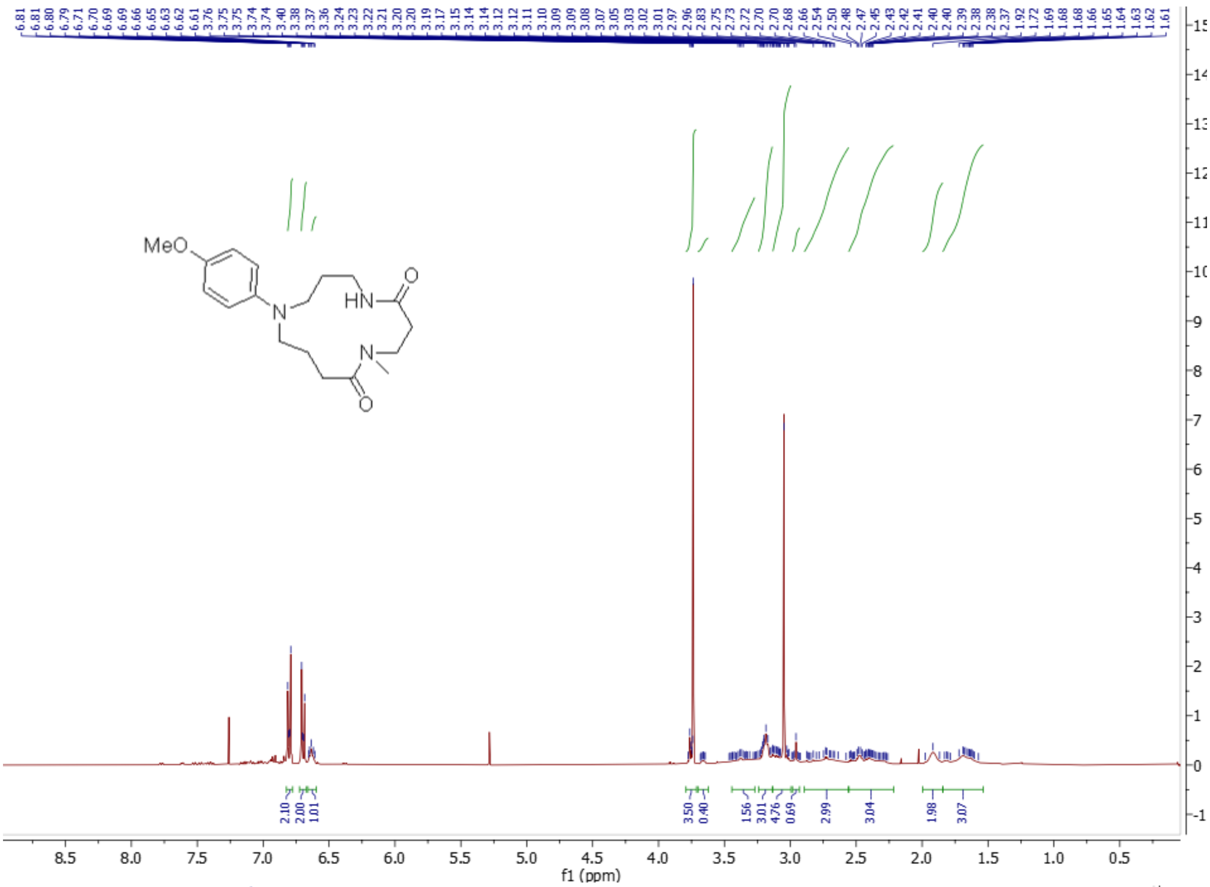

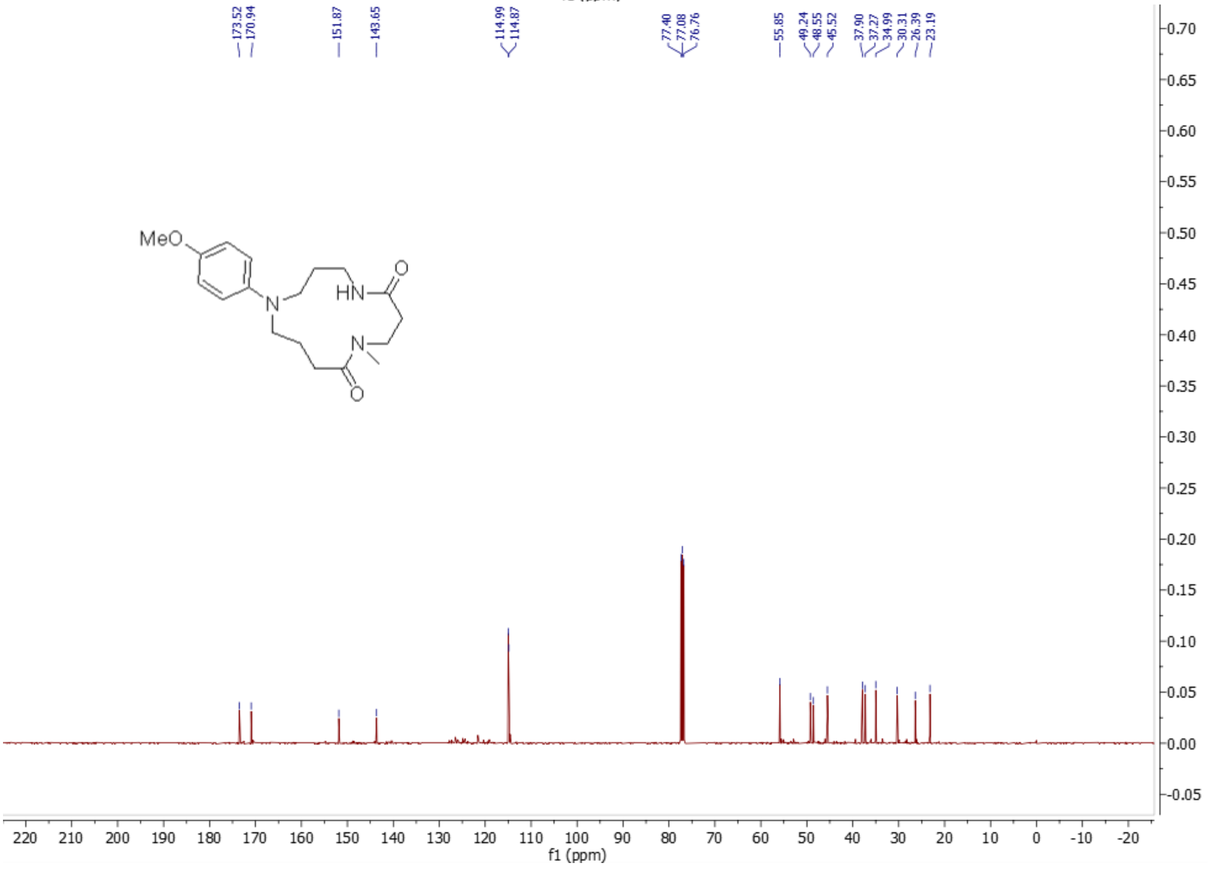

9-(4-Methoxyphenyl)-1,5,9-triazacyclotridecane-4,13-dione (**19e**) – in $CDCl_3$; 400 MHz for ^{1}H, 101 MHz for ^{13}C

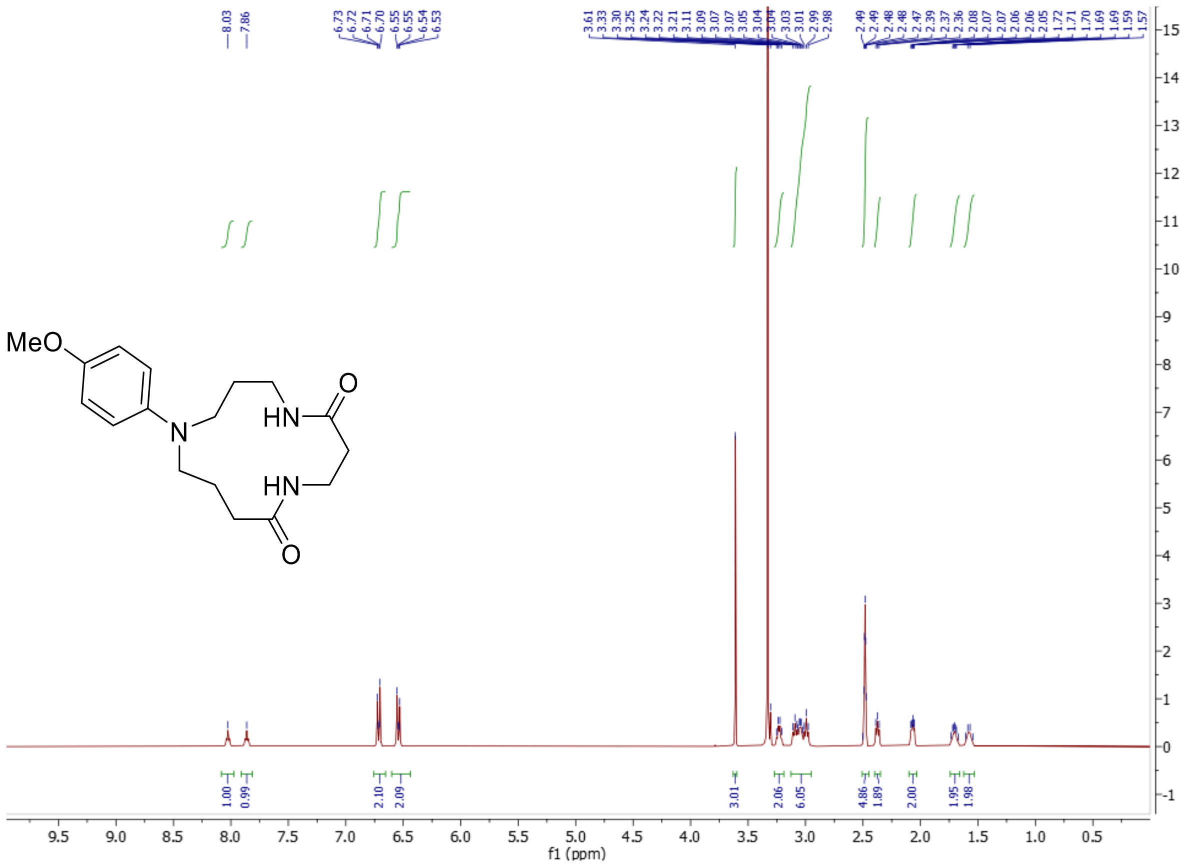

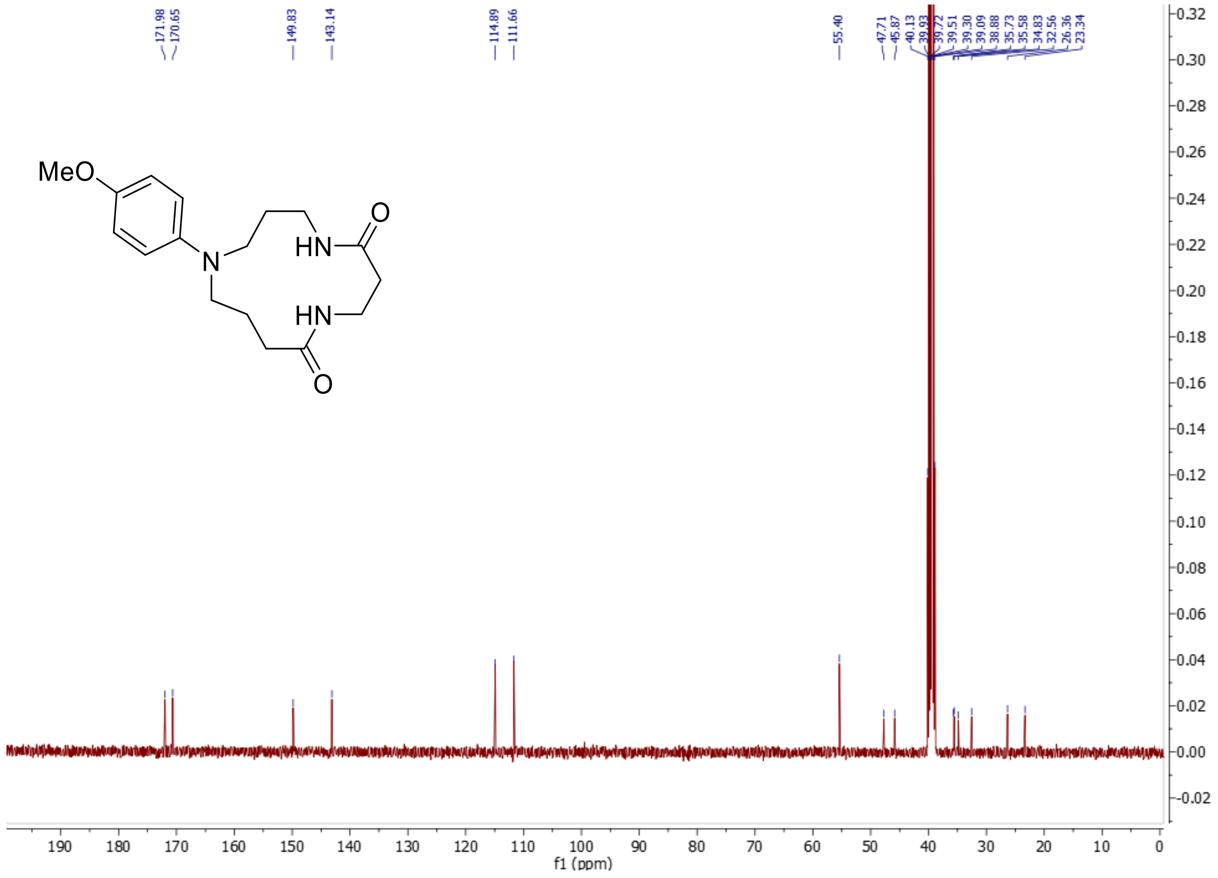

(*R*)-2-Phenyl-1,5,9-triazacyclotridecane-4,13-dione (21) – in d4-MeOD; 400 MHz for ^{1}H, 101 MHz for ^{13}C

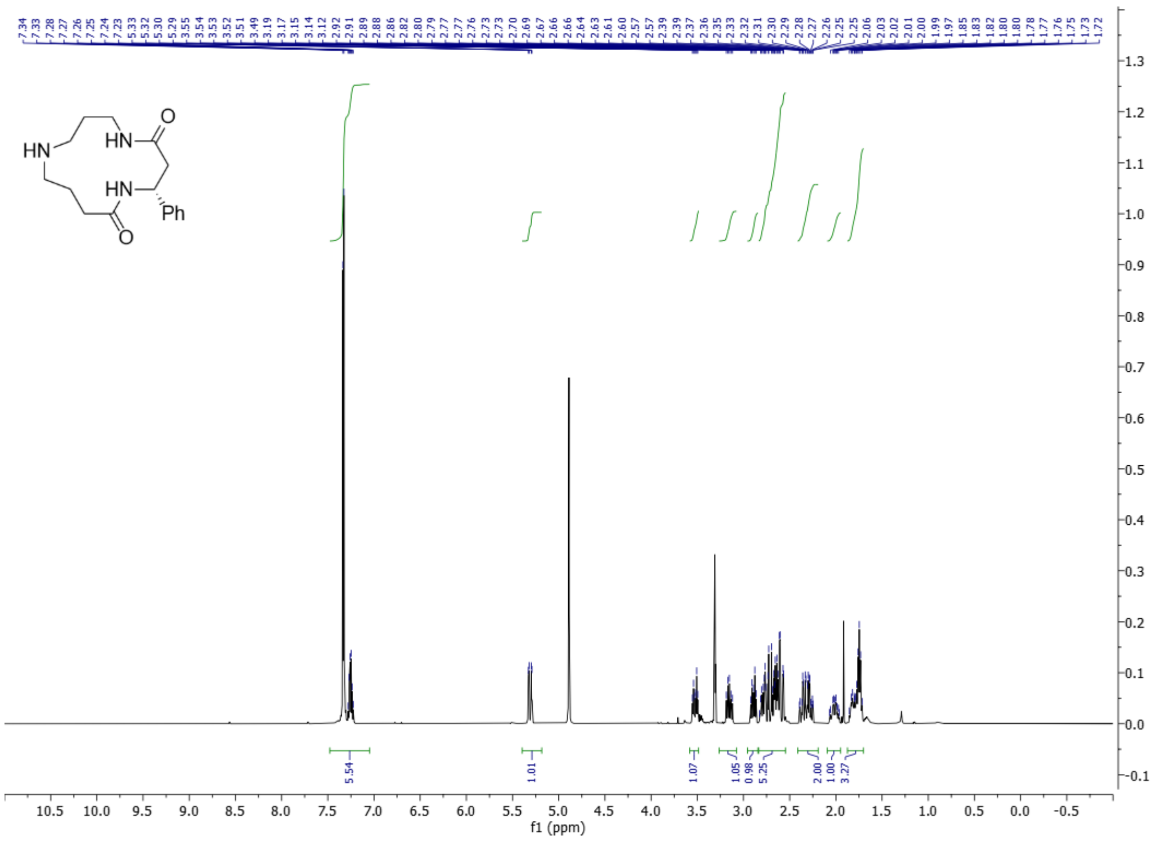

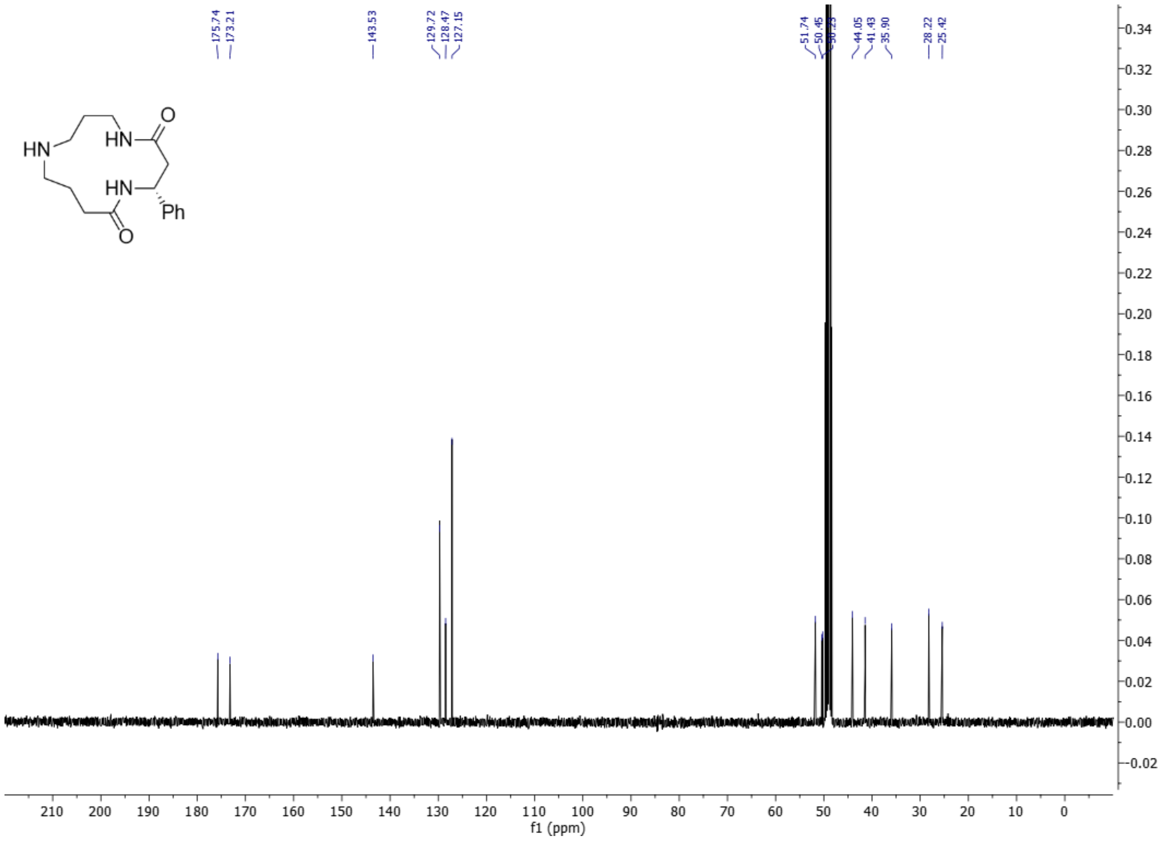

(*R*)-9-(Furan-3-carbonyl)-2-phenyl-1,5,9-triazacyclotridecane-4,13-dione (2) – in d_6 DMSO; 700 MHz for ^{1}H, 176 MHz for ^{13}C

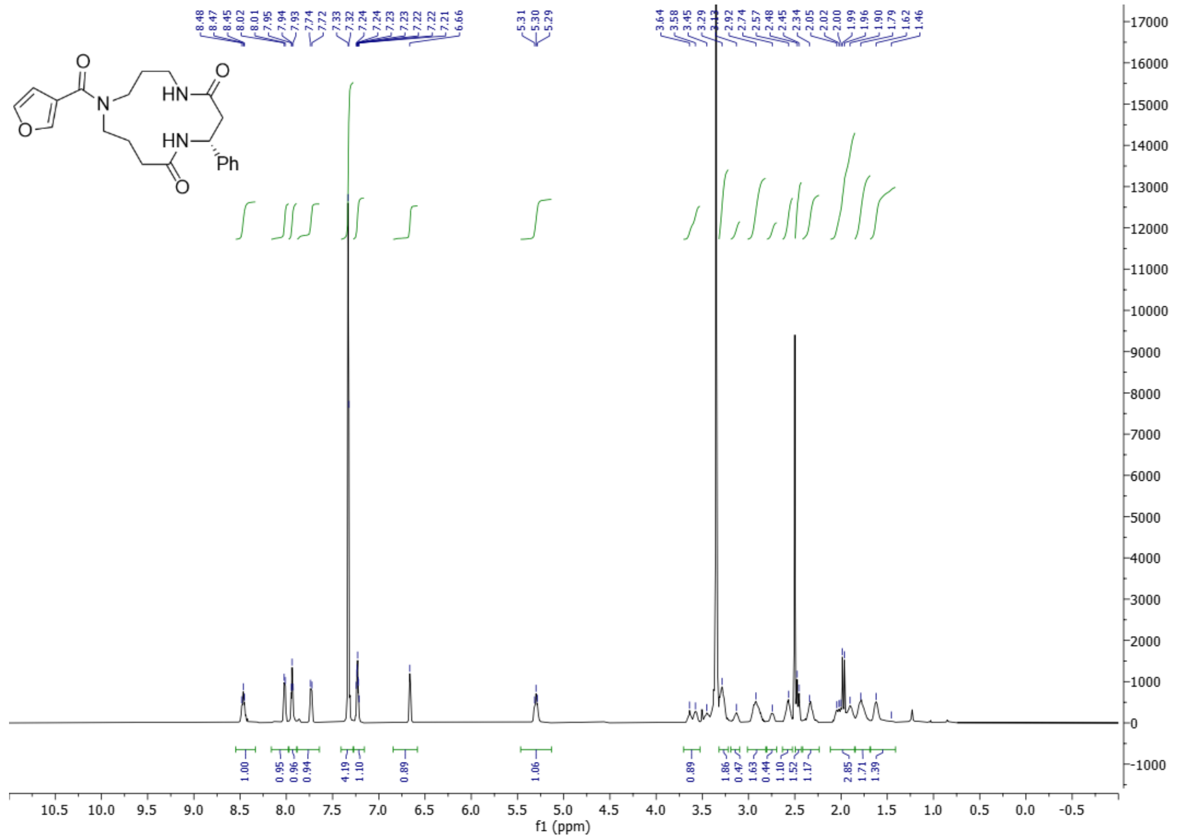

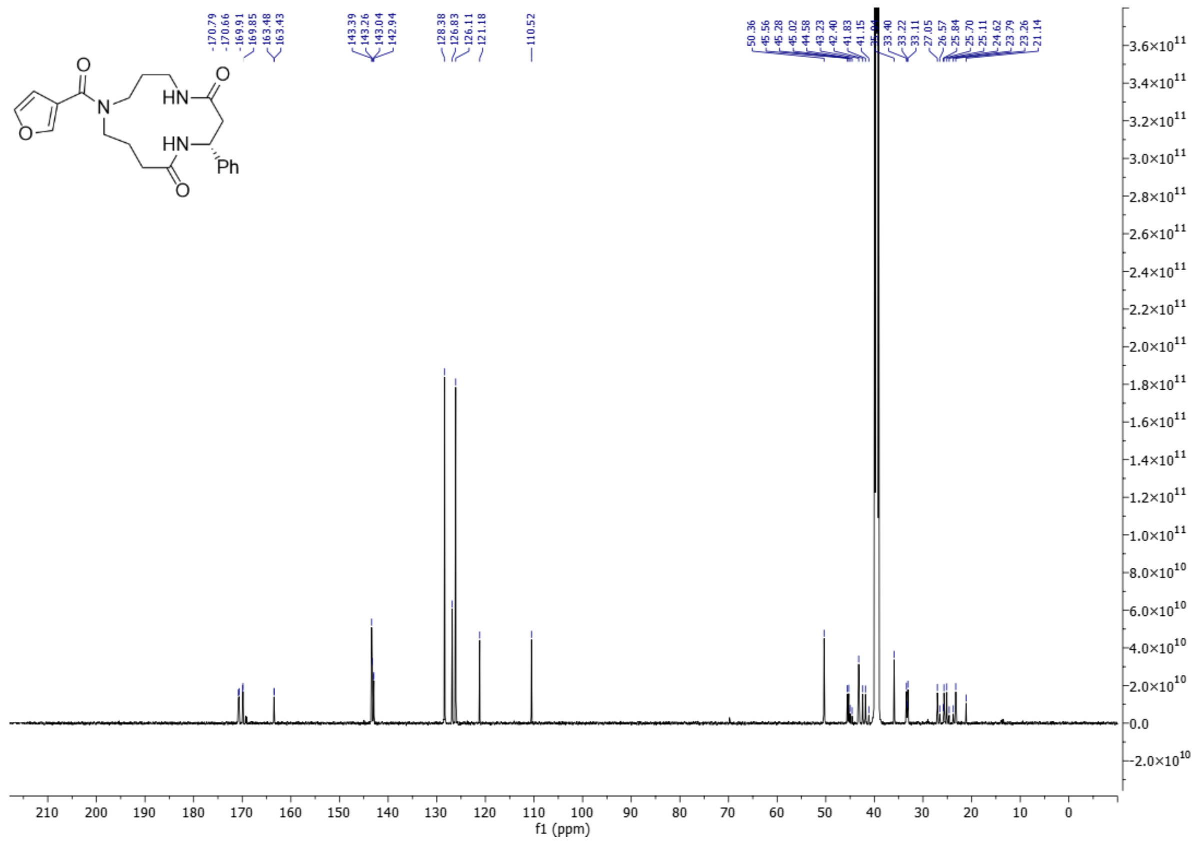

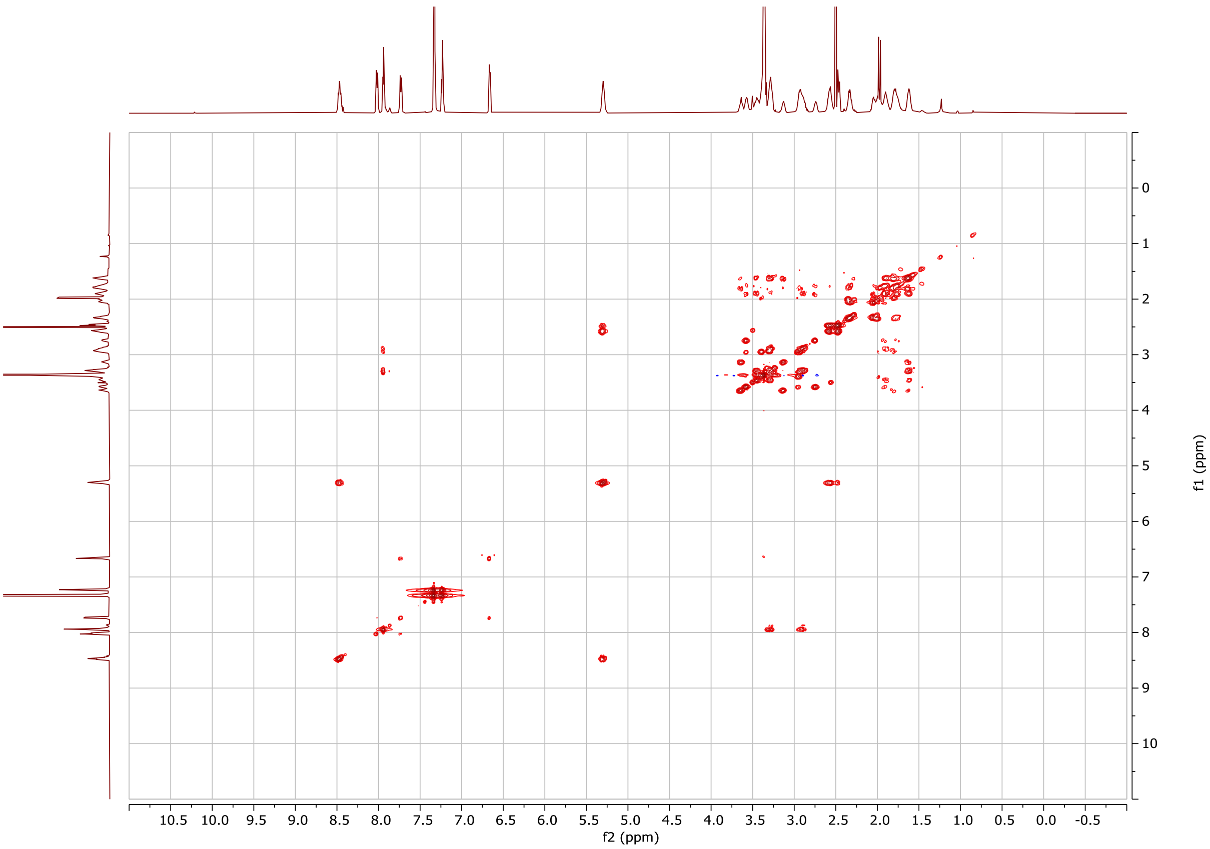
f2 (ppm)
f1 (ppm)

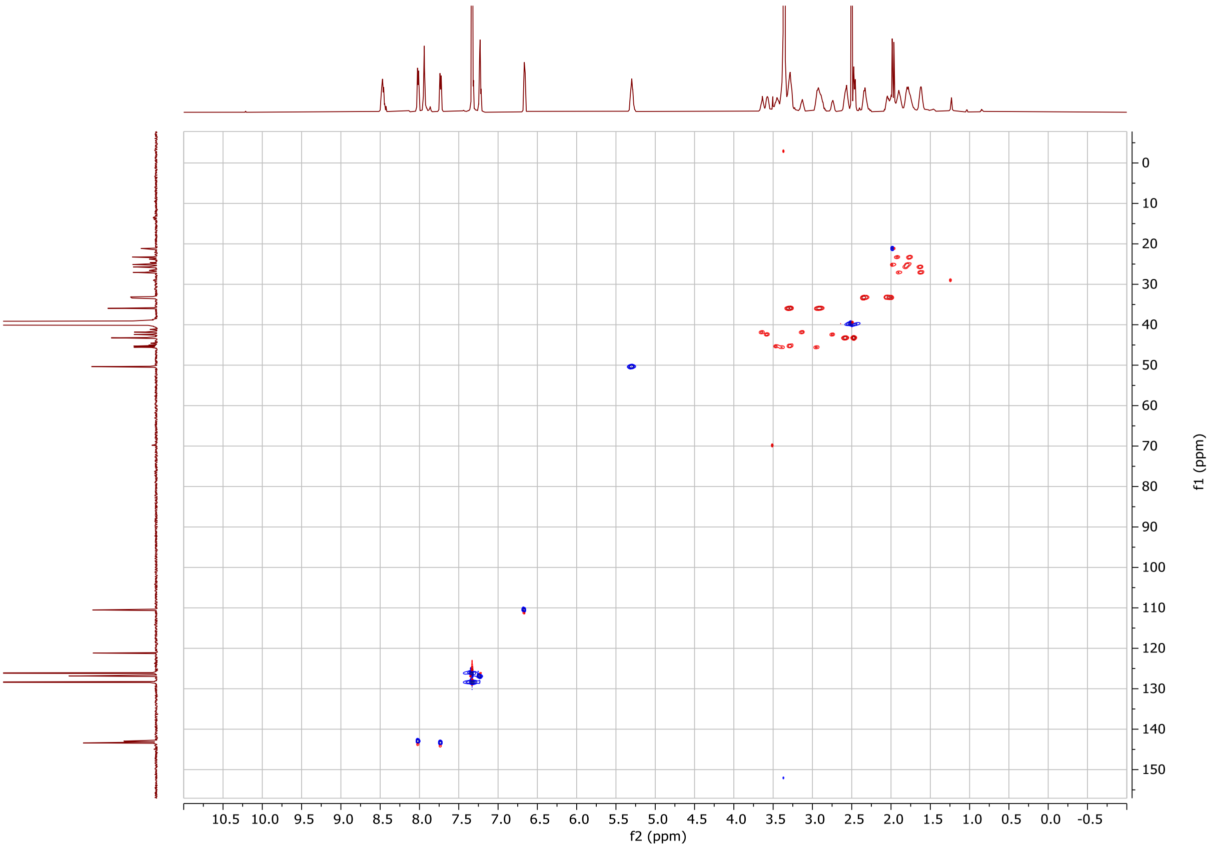
f2 (ppm)
f1 (ppm)

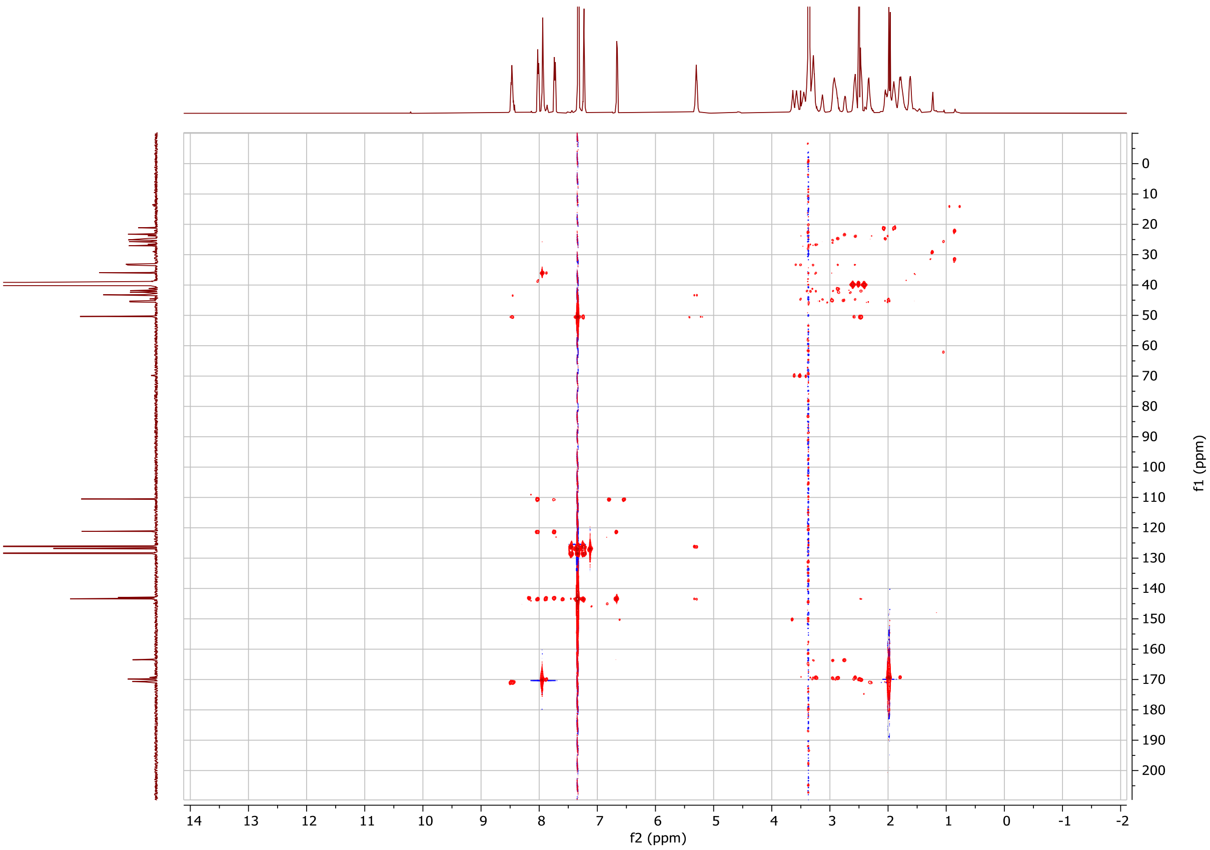

Image of weak (low solubility) ^{1}H NMR of 2 in d_4-MeOD

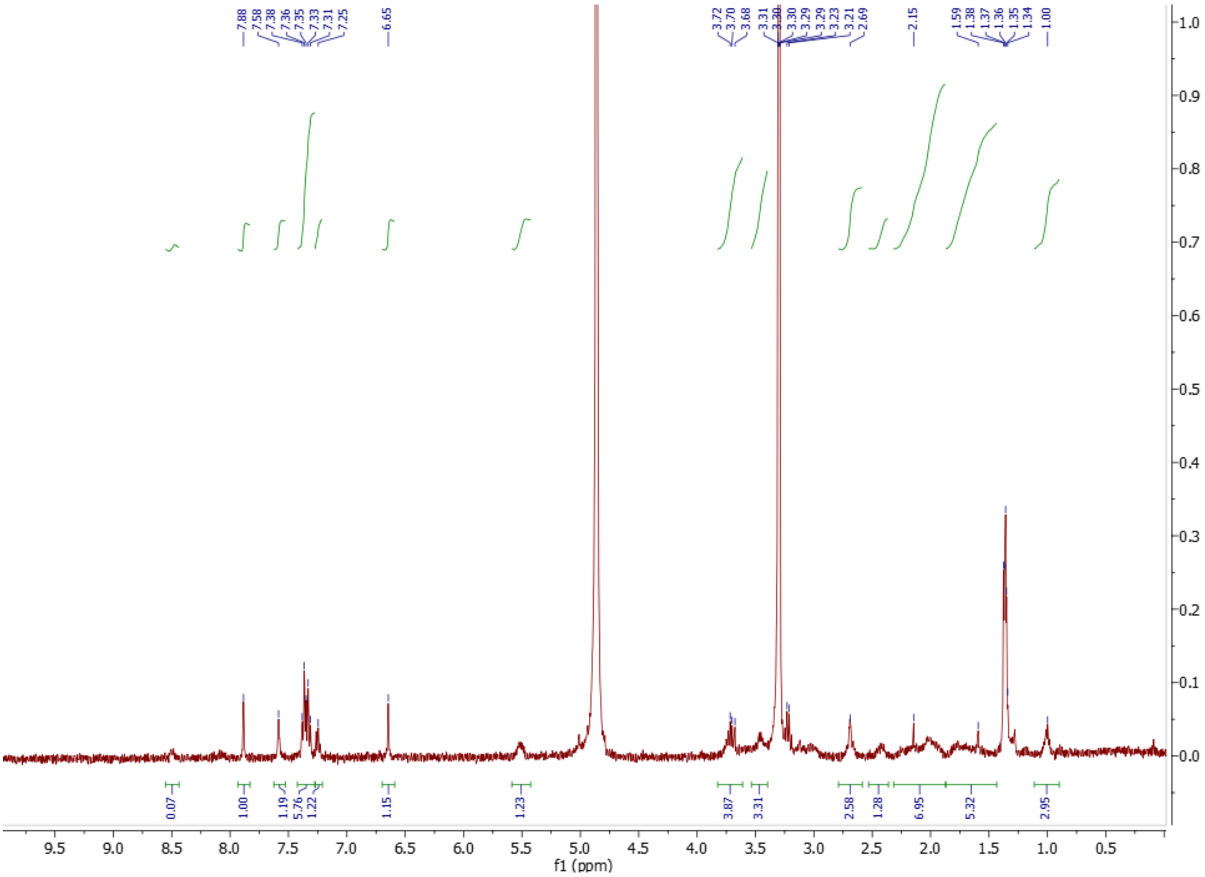

9-Benzoyl-2-phenyl-1,5,9-triazacyclotridecane-4,13-dione (**22a**) in d_6-DMSO; 400 MHz for ^{1}H, 101 MHz for ^{13}C

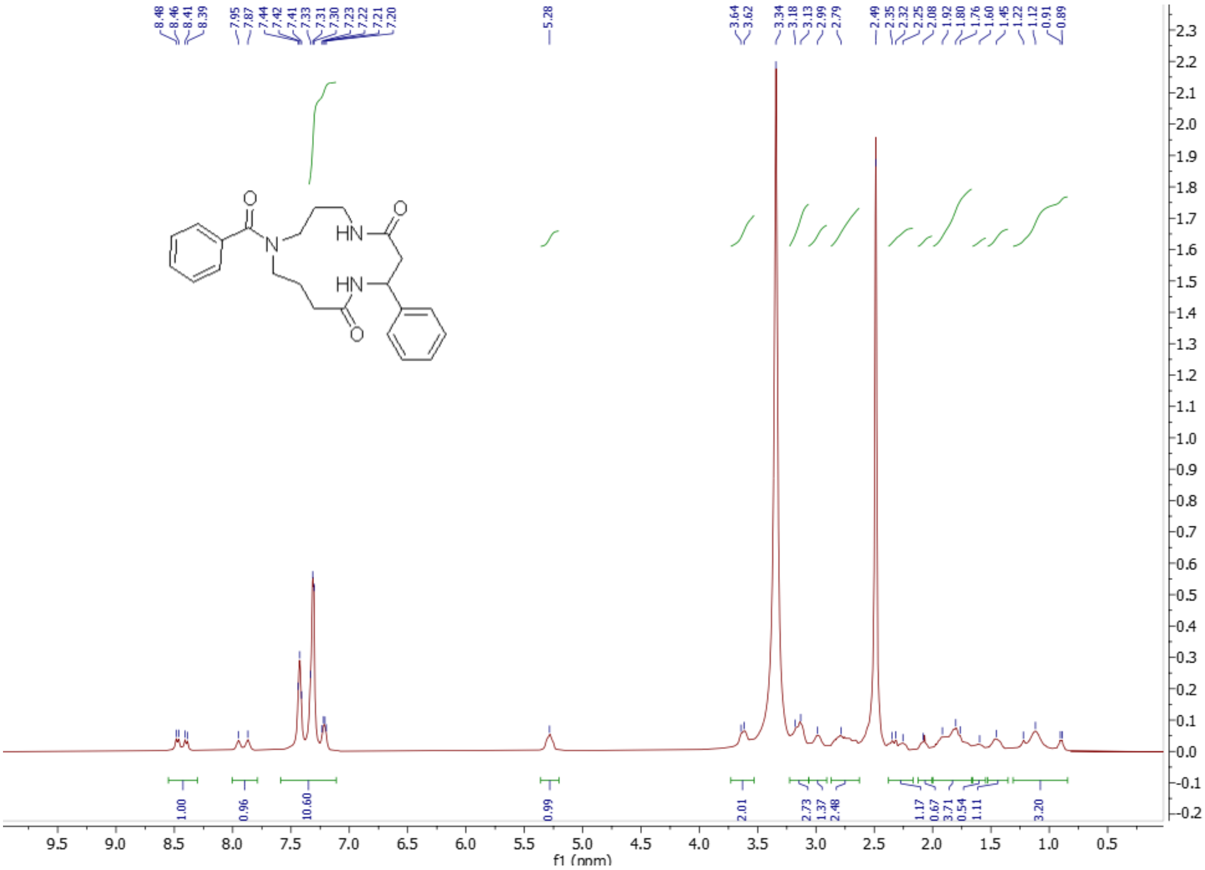

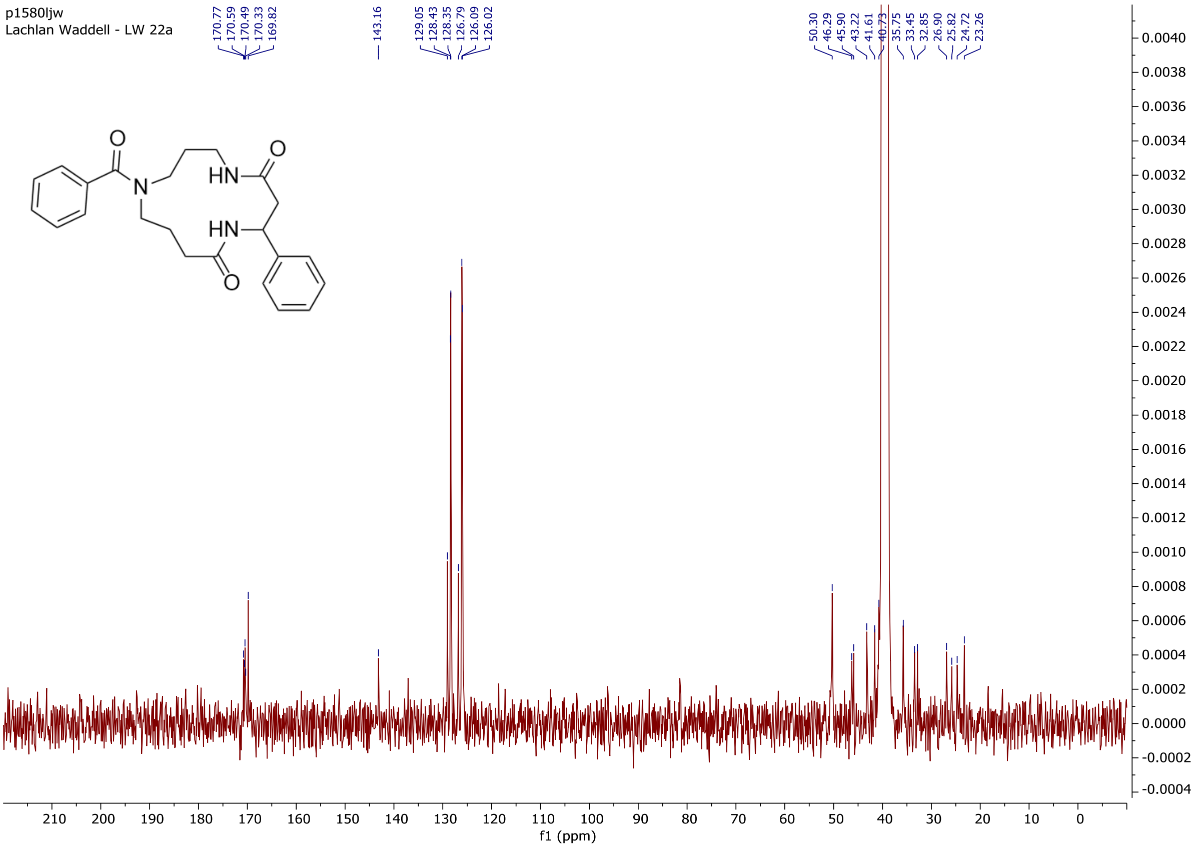

9-Cinnamoyl-2-phenyl-1,5,9-triazacyclotridecane-4,13-dione (**22b**) in d_6-DMSO; 700 MHz for ^1H, 176 MHz for ^{13}C

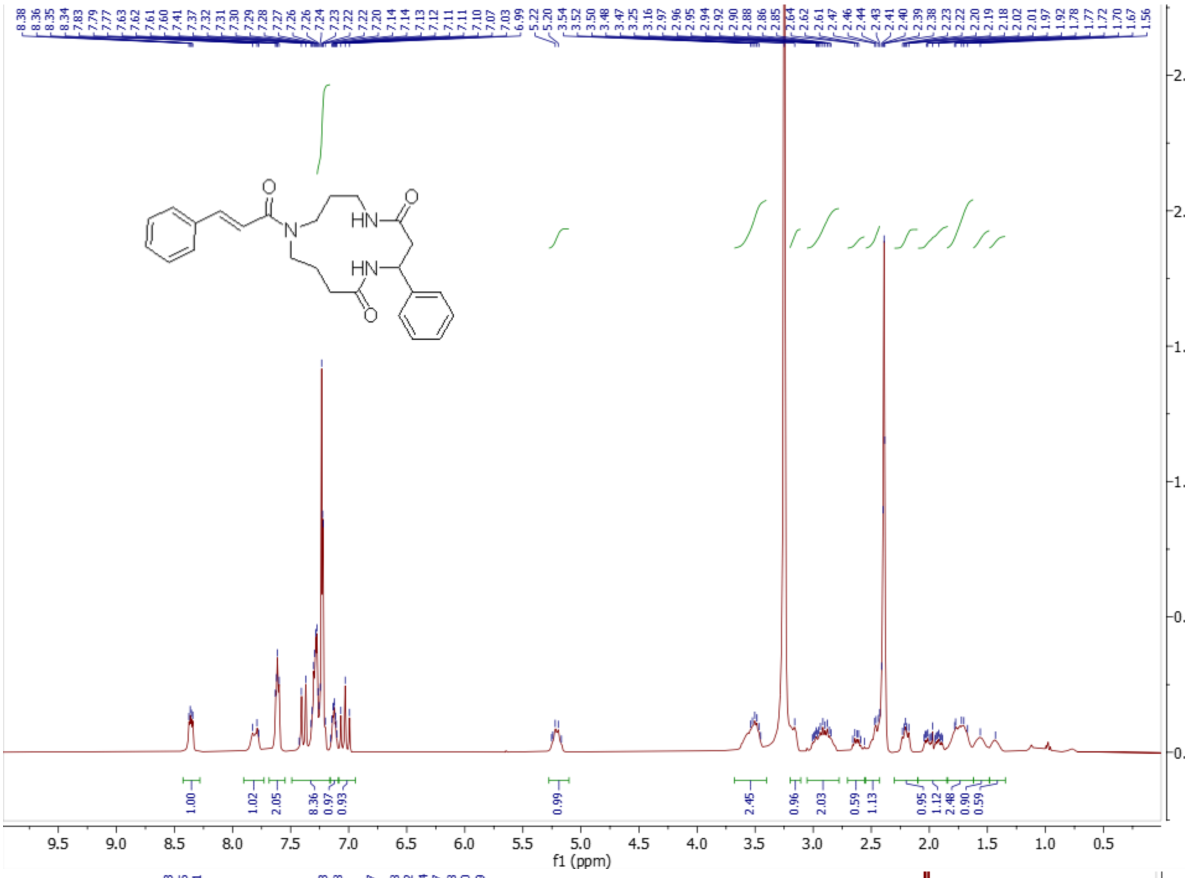

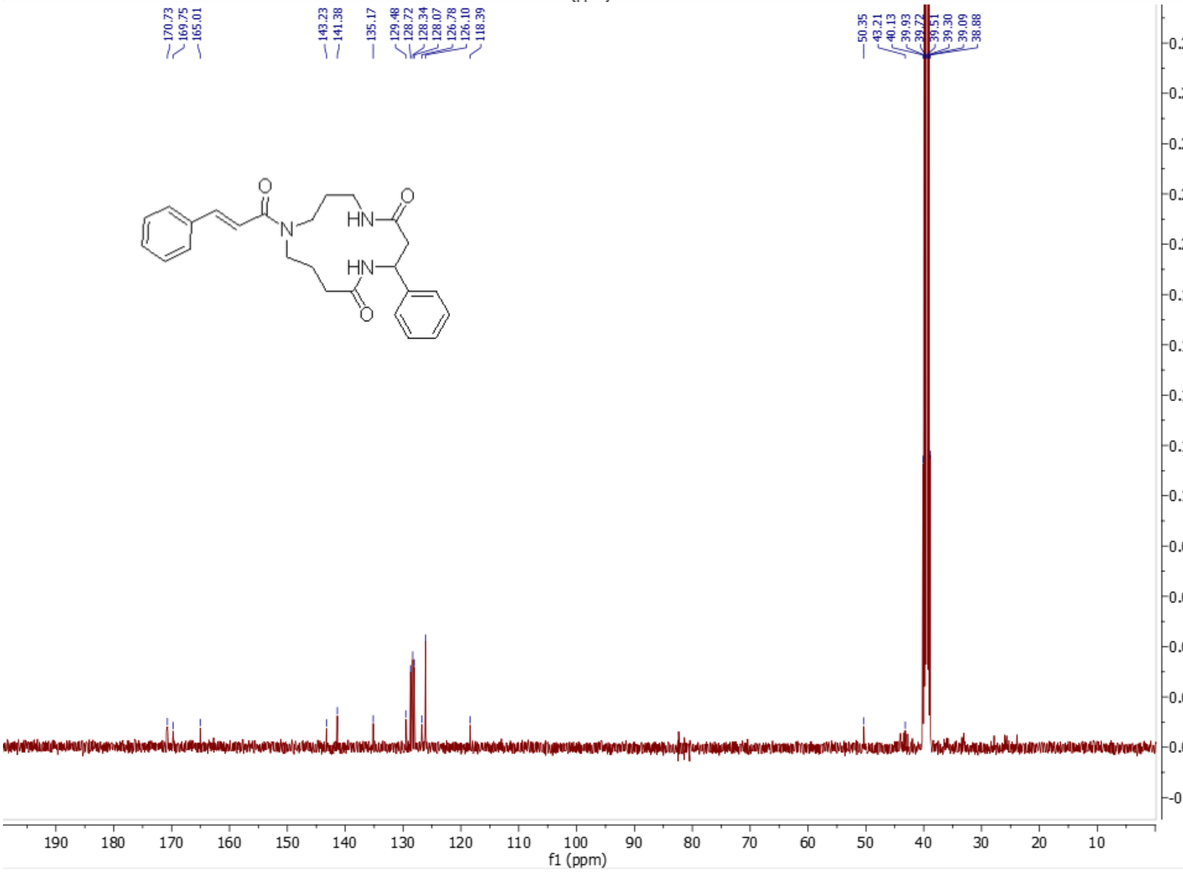

9-(2-(4-(Dimethylamino)phenyl)acetyl)-2-phenyl-1,5,9-triazacyclotridecane-4,13-dione (**22c**) in d_6-DMSO; 700 MHz for ^1H, 176 MHz for ^{13}C

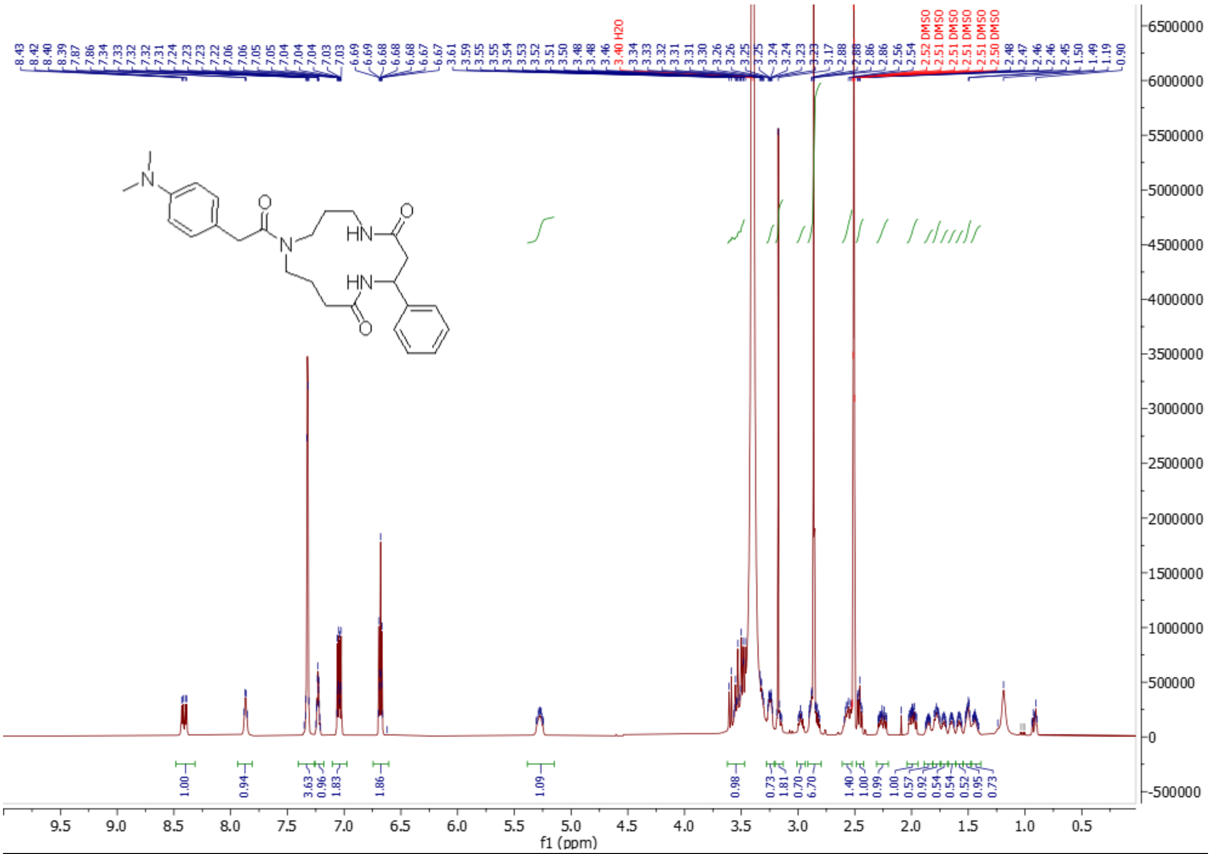

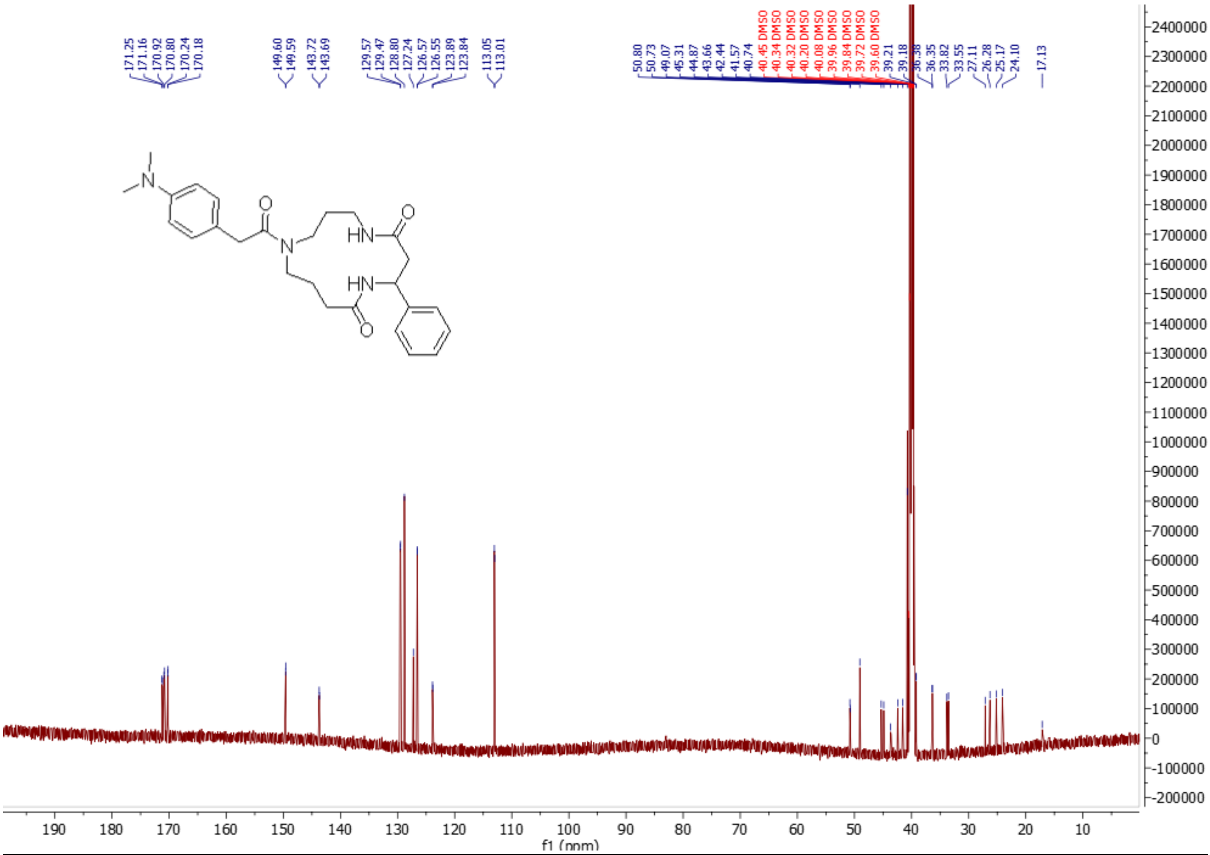

(*S*)-2-Benzyl-8-(4-methoxyphenyl)-1,4,8-triazacyclododecane-3,12-dione (19f) – in $CDCl_3$; 400 MHz for ^1H, 101 MHz for ^{13}C

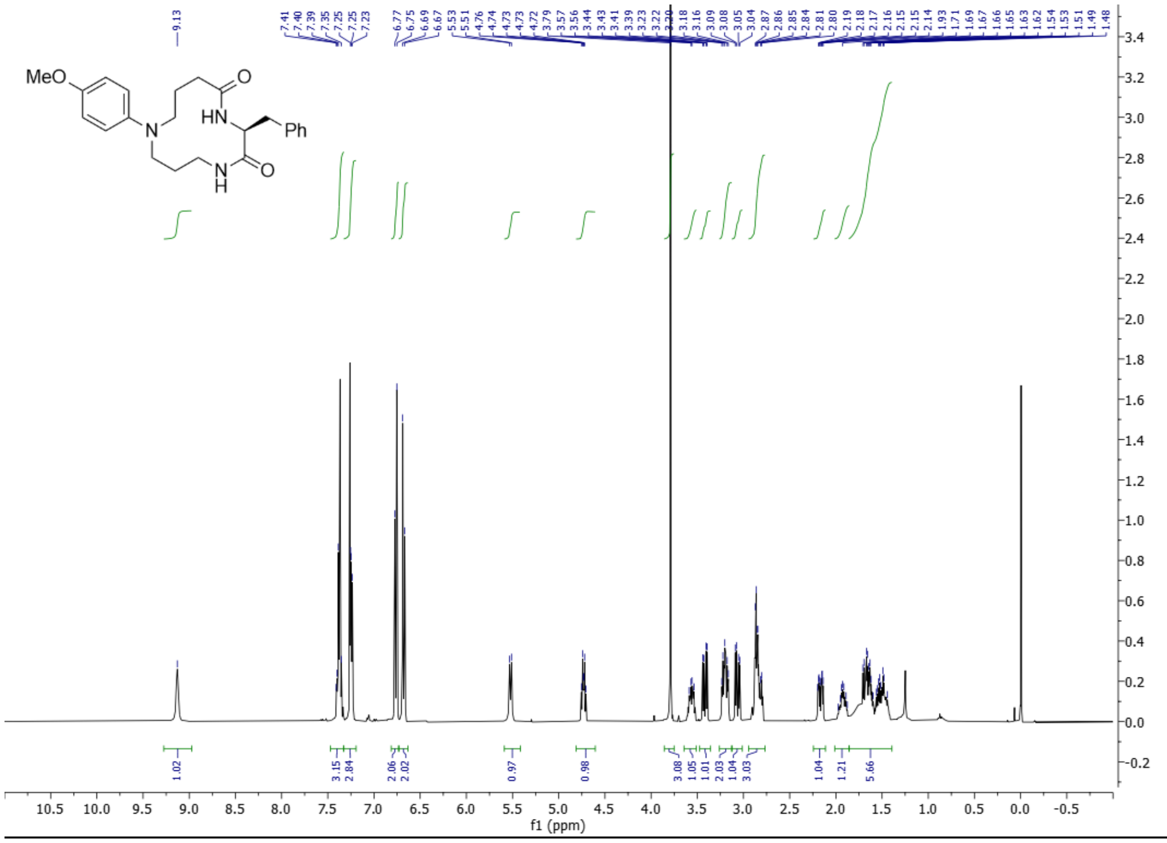

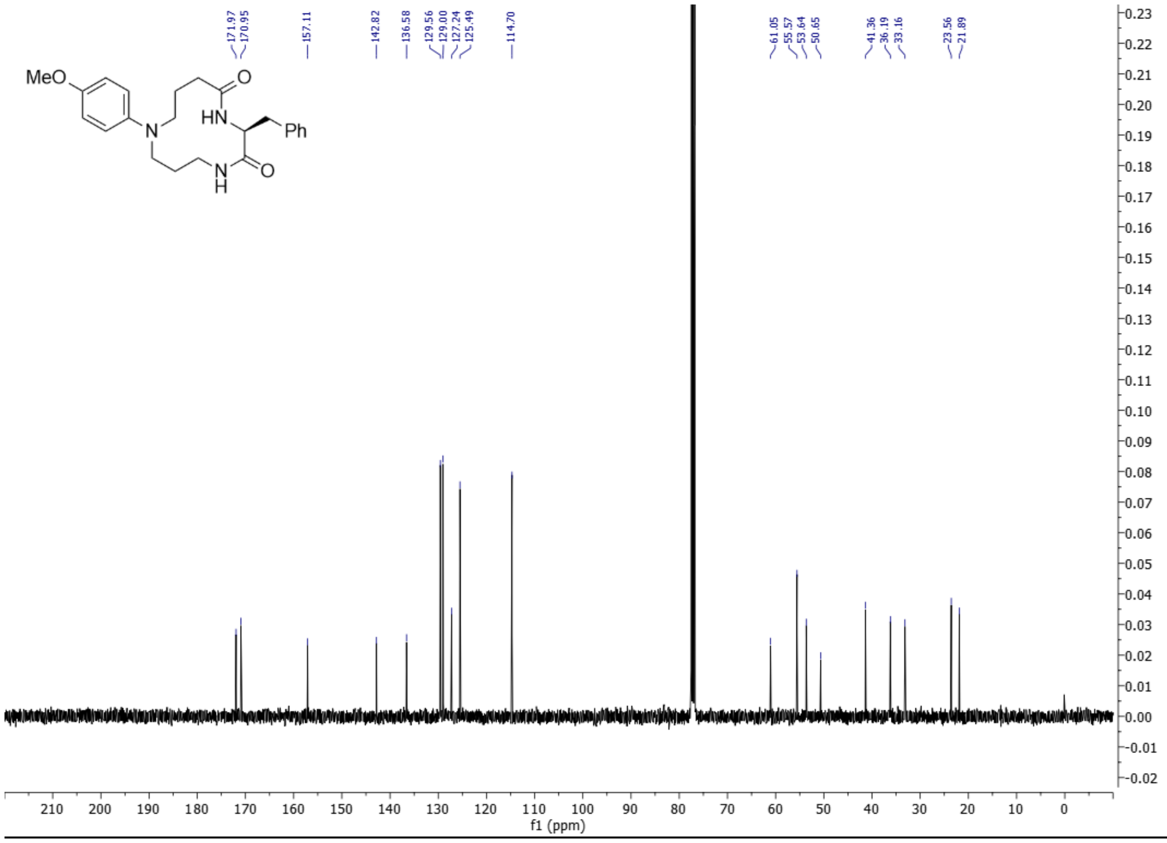

(*S*)-2-Benzyl-1,4,8-triazacyclododecane-3,12-dione (23) – in d_4-MeOD; 400 MHz for ^1H, 101 MHz for ^{13}C

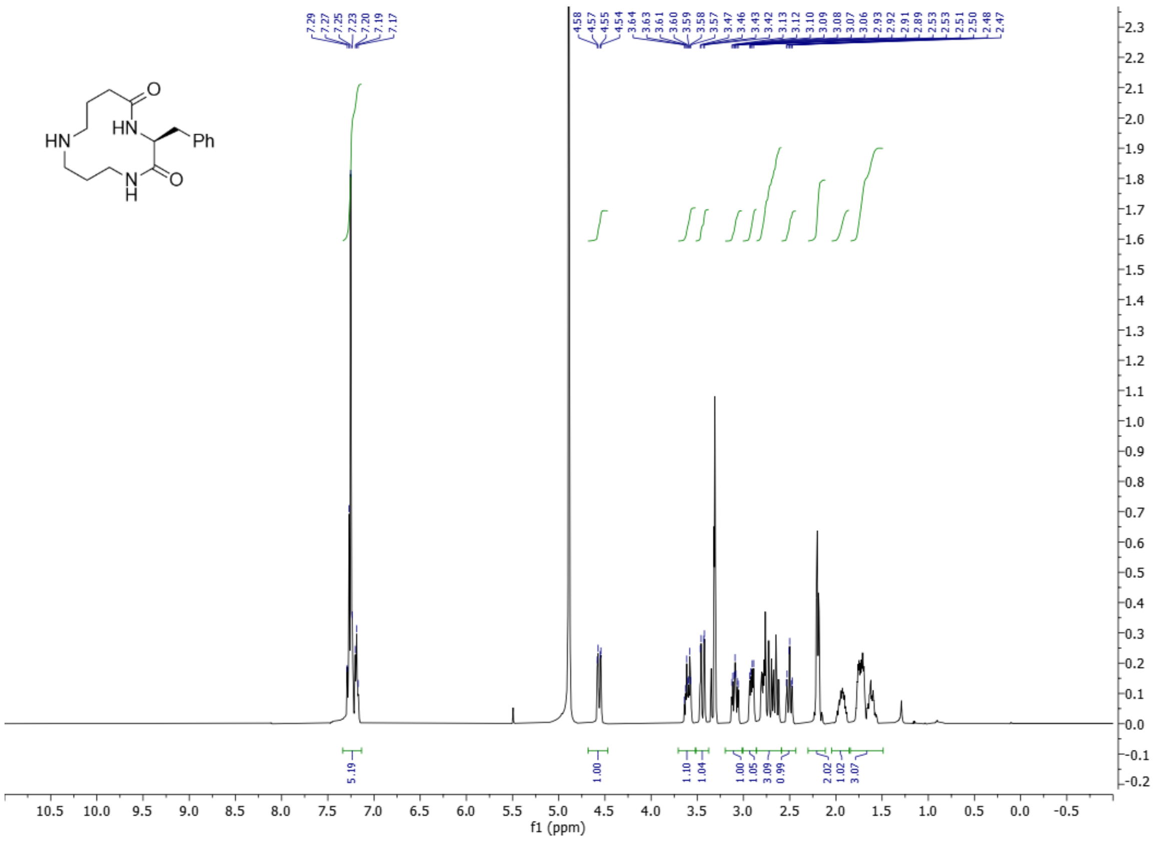

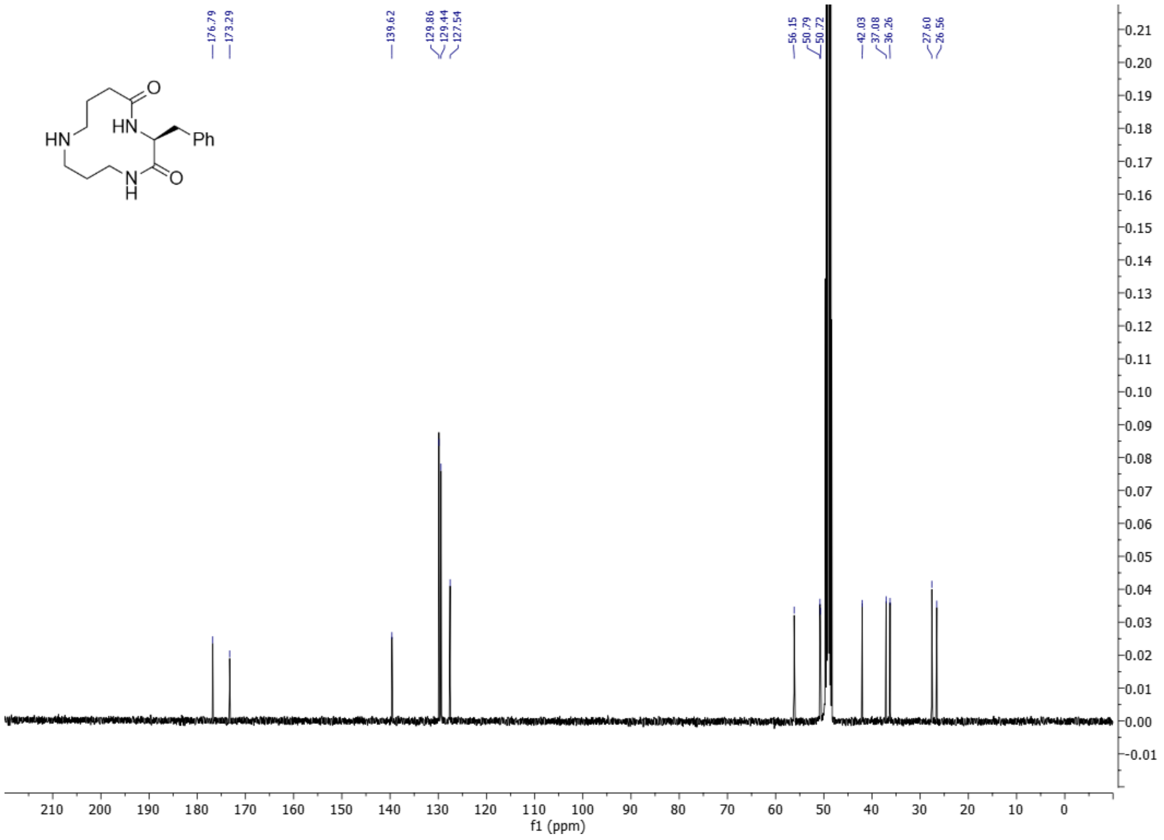

(*S*)-2-Benzyl-8-(furan-3-carbonyl)-1,4,8-triazacyclododecane-3,12-dione (24) – in d_4-MeOD; 400 MHz for ^1H, 101 MHz for ^{13}C

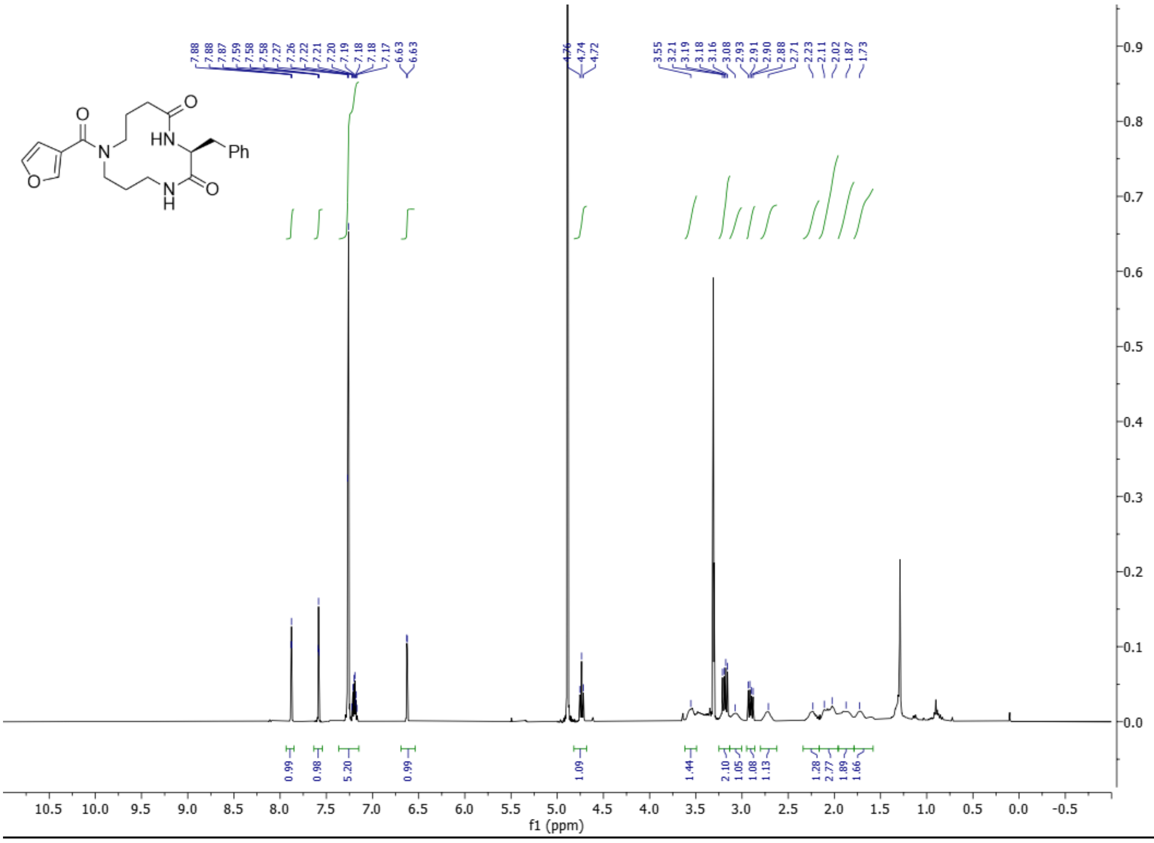

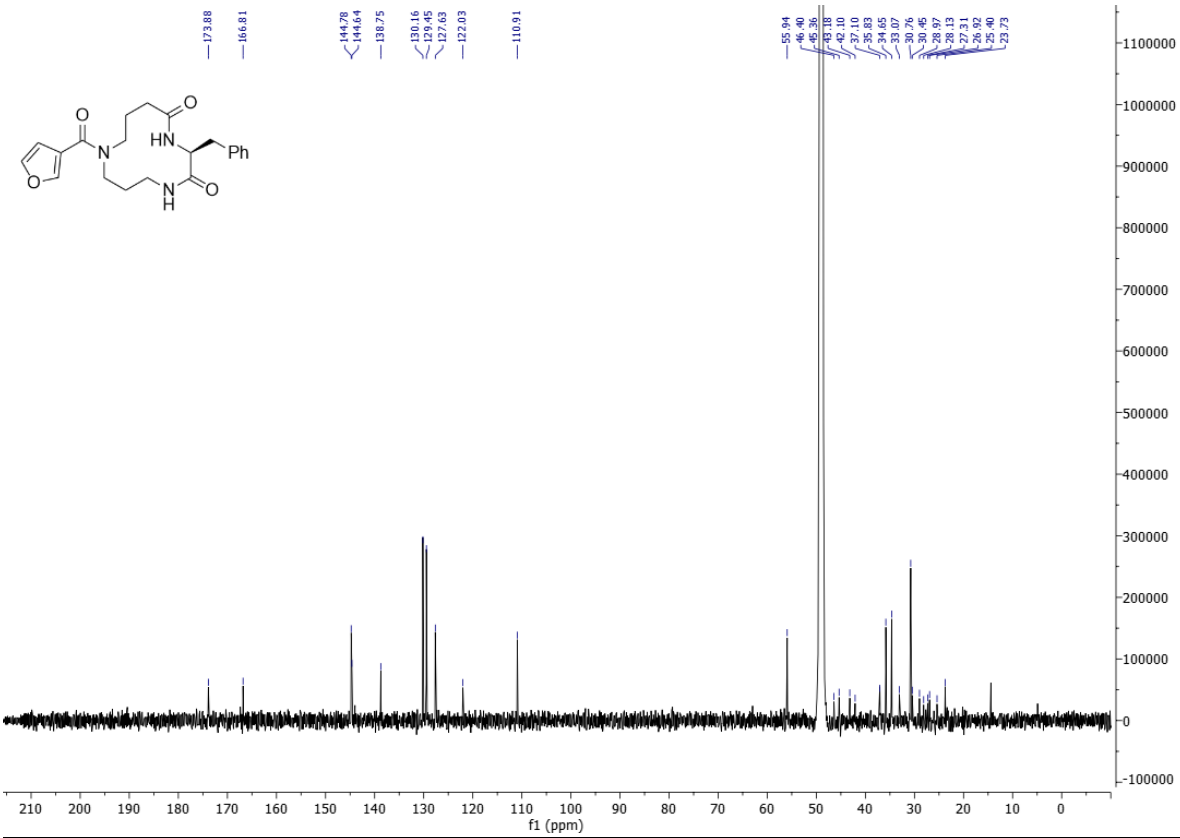

(*S*)-2-Benzyl-8-(furan-3-carbonyl)-1,4,8-triazacyclododecane-3,12-dione (24) – in d_6-DMSO; 400 MHz for ^1H, 101 MHz for ^{13}C

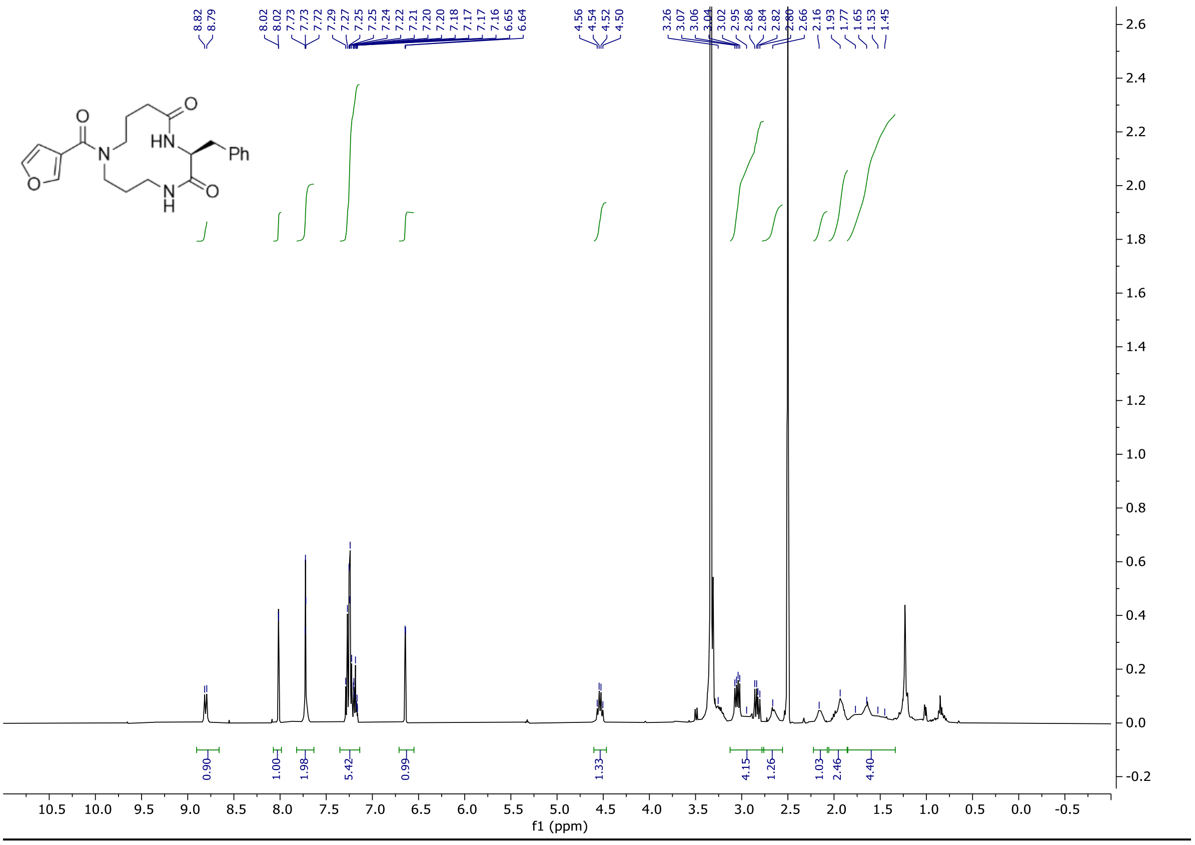

7) References

1) K. Y. Palate, Z. Yang, A. C. Whitwood, W. P. Unsworth. *RSC Chem. Biol.*, 2022, **3**, 334–340.

2) T. C. Stephens, M. Lodi, A. Steer, Y. Lin, M. Gill, W. P. Unsworth. *Chem. Eur. J*. 2017, **23**, 13314–13318.

3) J. Liu, Q. Wu, J. Shu, R. Zhang, L. Liu. *Chem. Nat. Compd.*, 2020, **56**, 496–499.

4) J. Liu, Q. Wu, J. Shu and R. Zhang. Preparation method of anti-inflammatory macrocyclic polyamine alkaloid celacarfurine. CN111484485B, April 5, 2022.

5) P. Kuehne, A. Guggisberg, M. Hesse, M. *Helv. Chim. Acta.*, 1997, **80**, 1802–1808.
